# Total Synthesis of the Antimitotic Marine Macrolide (−)-Leiodermatolide**

**DOI:** 10.1002/anie.201310164

**Published:** 2014-01-30

**Authors:** Ian Paterson, Kenneth K-H Ng, Simon Williams, David C Millican, Stephen M Dalby

**Affiliations:** University Chemical Laboratory, University of CambridgeLensfield Road, Cambridge, CB2 1EW (UK)

**Keywords:** aldol reaction, antitumor agents, cross-coupling, macrolides, total synthesis

## Abstract

Leiodermatolide is an antimitotic macrolide isolated from the marine sponge Leiodermatium sp. whose potentially novel tubulin-targeting mechanism of action makes it an exciting lead for anticancer drug discovery. In pursuit of a sustainable supply, we report a highly stereocontrolled total synthesis (3.2 % yield) based on a convergent sequence of palladium-mediated fragment assembly and macrolactonization. Boron-mediated aldol reactions were used to configure the three key fragments **2**, **5**, and **6** by employing the appropriate enantiomer of the lactate-derived ketone **7**.

Tubulin-targeting compounds are perhaps the most validated subset of clinically important anticancer agents, with natural products and analogues representing the mainstay for current chemotherapy,[1–3] recently supplemented by the approval of the antibody–maytansinoid conjugate Kadcyla (trastuzumab emtansine).[2] Leiodermatolide (**1**; Scheme 1) was isolated (0.001 % wet weight) by the Wright group in 2008 from the lithistid sponge *Leiodermatium* sp. collected by submersible off the Florida coastline.[4a] Leiodermatolide exhibits potent antiproliferative activity against a panel of human cancer cell lines (e.g. IC_50_=3.3 nm for A549 lung adenocarcinoma cells, 5.0 nm for PANC-1 pancreatic carcinoma cells), whilst showing reduced toxicity to normal cells. This activity appears to be mediated through the disruption of tubulin dynamics to induce cell-cycle arrest in the G2/M phase and apoptosis. Although the exact mechanism of action of leiodermatolide is currently unknown, it is clearly distinct from that of other tubulin-targeting drugs. Thus, leiodermatolide could serve as a promising lead compound for the development of new anticancer agents, provided a sustainable supply can be generated by chemical synthesis.[5–7]

**Scheme 1:**
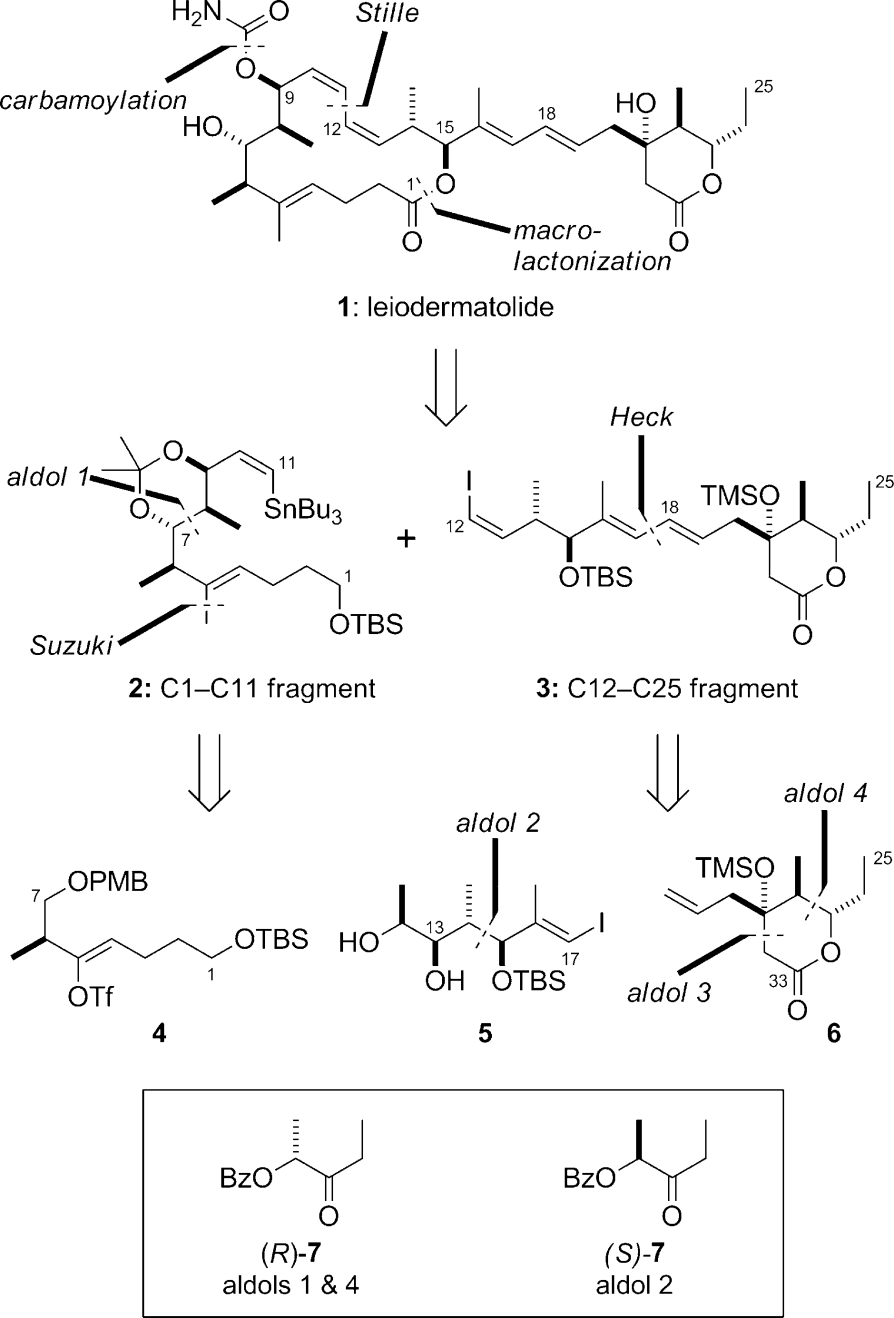
Retrosynthetic analysis and key fragments for the synthesis of leiodermatolide. Bz=benzoyl, PMB=*para*-methoxybenzyl, TBS=*tert*-butyldimethylsilyl, Tf=trifluoromethanesulfonyl, TMS=trimethylsilyl.

From a structural perspective, leiodermatolide features a triply unsaturated 16-membered macrolactone appended at C9 with a carbamate group and at C15 with an *E*,*E*-dienyl side chain terminating in a δ-lactone ring. This unique structure incorporates a total of nine stereocenters. In association with the Wright research group,[4b] we elucidated the relative configuration of leiodermatolide by using a combination of homo- and heteronuclear NMR spectroscopic analysis, molecular modeling, and computational DP4 NMR prediction.[8] The resulting assignment for the C1–C16 region was further supported by our synthesis of a macrocyclic fragment with a truncated side chain,[5] whereas an alternative stereostructure could be ruled out on the basis of synthetic studies reported earlier.[6a] The full configuration of the isolated C1–C16 and C20–C25 stereoclusters was only recently tied down with the first total synthesis of (−)-leiodermatolide (**1**) by the Fürstner research group employing an elegant strategy based on ring-closing alkyne metathesis.[7] We now report a highly convergent total synthesis of (−)-leiodermatolide implementing a complementary macrolactonization strategy that also features the extensive application of our versatile lactate aldol chemistry[9] along with a variety of palladium-mediated coupling reactions.[10]

Building on the lessons learned from earlier synthetic efforts directed towards the macrocyclic core,[5] Scheme 1 depicts the main retrosynthetic disconnections and key fragments **2**–**6** devised for the synthesis of leiodermatolide. The structure was initially simplified by disassembly of the 10*Z*,12*Z*-diene region and opening of the macrolactone ring in **1** to reveal the C1–C11 vinyl stannane **2** and the C12–C25 vinyl iodide **3** containing the entire side chain for a planned late-stage Stille coupling. The former fragment was then envisaged to be available by elaboration of vinyl triflate **4** through a Suzuki-type methylation and an *anti*-selective aldol reaction using (*R*)-**7**. The more elaborate fragment **3** would arise in turn through stereocontrolled installation of the 16*E*,18*E* diene by a Heck coupling between vinyl iodide **5** and the correctly configured allyl-substituted δ-lactone **6**, constructed using (*S*)- and (*R*)-**7**, respectively.

The synthesis of vinyl stannane **2** utilized Roche ester derivative **8** as the source of the C6 methyl-bearing stereocenter (Scheme 2).[11] The required 4*E*-configured trisubstituted alkene was first introduced via the corresponding stereodefined vinyl triflate **4**. In practice, controlled addition of TBSO(CH_2_)_4_MgBr to **8** provided the required ketone (88 %), which was converted into **4** with high selectivity (82 %, >20:1 *Z*/*E*) by treatment of the kinetically generated lithium enolate (LiHMDS) with the Comins reagent.[12] After screening various methods for methylation, Suzuki coupling of **4** with trimethylboroxine[13] (cat. [Pd(PPh_3_)_4_], K_2_CO_3_) was found to proceed well to afford the *E* alkene **9** (96 %, >20:1 *E*/*Z*). Following cleavage of the PMB ether (DDQ) and Dess–Martin oxidation (69 %), the resulting aldehyde **10** was treated with the *E* dicyclohexylboron enolate derived from (*R*)-**7** (*c*-Hex_2_BCl, Et_3_N).[9] This matched aldol addition[14] afforded the *anti* adduct **11** (96 %, d.r.>20:1) with a high level of control over the C7/C8 stereocenters.

**Scheme 2:**
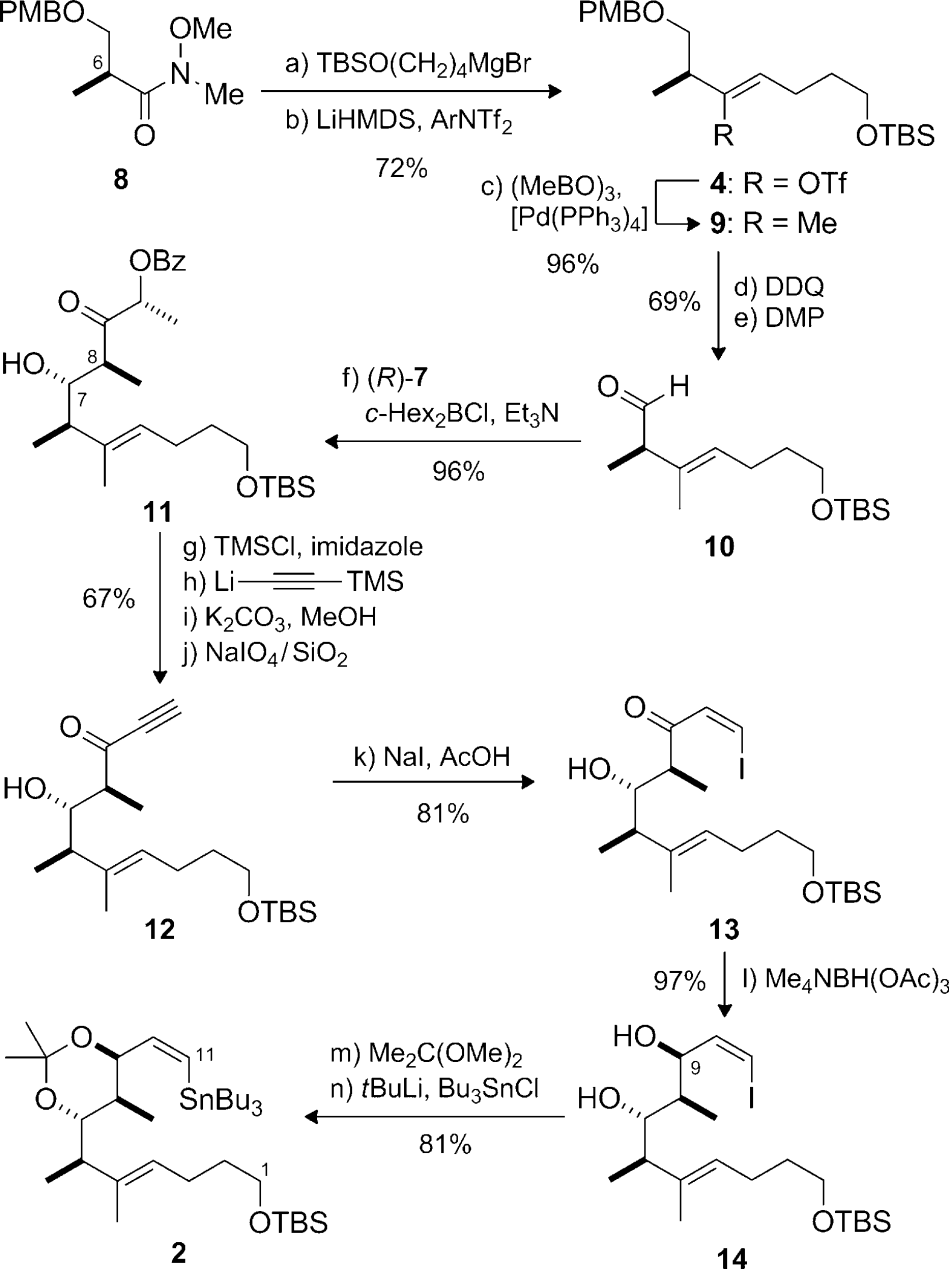
Preparation of vinyl stannane 2. Reagents and conditions: a) TBSO(CH_2_)_4_MgBr, THF, −78 °C, 88 %; b) LiHMDS, THF; Comins reagent, −78→−20 °C, 82 %; c) (MeBO)_3_, [Pd(PPh_3_)_4_] (10 mol %), K_2_CO_3_, dioxane, 50 °C, 96 % (>20:1 *E*/*Z*); d) DDQ, pH 7 buffer, CH_2_Cl_2_, 84 %; e) DMP, NaHCO_3_, CH_2_Cl_2_, 82 %; f) (*R*)-7, *c*-Hex_2_BCl, Et_3_N, Et_2_O, −78→−20 °C, 96 % (d.r.>20:1); g) TMSCl, imid, CH_2_Cl_2_, 96 %; h) LiC=CTMS, THF, −78 °C; i) K_2_CO_3_, MeOH; j) NaIO_4_/SiO_2_, CH_2_Cl_2_, 69 % over 3 steps; k) NaI, AcOH, THF, 81 % (8:1 *Z*/*E*); l) Me_4_NBH(OAc)_3_, MeCN, AcOH (3:1), −30 °C, 97 % (d.r.>20:1); m) Me_2_C(OMe)_2_, PPTS, CH_2_Cl_2_, 99 %; n) *t*BuLi, Bu_3_SnCl, Et_2_O, −78 °C, 81 %. Comins reagent=2-(Tf_2_N)-5-Cl(C_5_H_3_N), DDQ=2,3-dichloro-5,6-dicyano-1,4-benzoquinone, DMP=Dess–Martin periodinane, HMDS=hexamethyldisilazide, PPTS=pyridinium *para*-toluenesulfonate.

Next, **11** was converted into ynone **12** (67 %) by a sequence of silylation, (trimethylsilyl)acetylide addition, basic methanolysis, and oxidative glycol cleavage.[15] The *Z* iodoenone could then be conveniently accessed through conjugate addition of NaI (AcOH, THF)[16] to **12** to afford **13** (8:1 *Z*/*E*, 81 % yield of the isolated *Z* isomer). To set the C9 configuration, Evans–Saksena reduction[17] (Me_4_NBH(OAc)_3_, MeCN, AcOH) of **13** proceeded well to give the 1,3-*anti* diol **14** (97 %, d.r.>20:1). Although preliminary studies were discouraging,[5] a cyclic C7/C9 protecting group was selected; thus, differentiation of the diol for regioselective carbamate formation at C9 would be required in the final stages of the synthesis. Accordingly, diol **14** was first converted into its acetonide (Me_2_C(OMe)_2_, PPTS), and stannylation (*t*BuLi, Bu_3_SnCl, 81 %) then provided the C1–C11 fragment **2** (20 % yield over 14 steps) in readiness for installation of the 10*Z*,12*Z* diene of leiodermatolide.

The requisite Stille coupling partner **3** was prepared from chiral intermediates **5** and **6** (Scheme 3). Vinyl iodide **5** was readily secured by using a second boron-mediated aldol reaction, this time between (*S*)-**7**[9] and aldehyde **15**[18] to give the *anti* adduct **16** (90 %, d.r.>20:1). Silylation (TBSOTf) of **16**, selective reduction with LiAlH_4_ (d.r.>20:1), and methanolysis smoothly afforded diol **5** (91 % over 3 steps). Construction of the δ-lactone fragment **6** required the installation of three contiguous stereocenters, including the axial C21 allyl group. The C22/C23 configuration was set by a third boron-mediated aldol reaction, in this case between (*R*)-**7**[9] again and propanal to generate ketone **17** (94 %, d.r.>20:1). Following silylation (TBSOTf),[19] the addition of H_2_C=CHCH_2_MgBr gave a 1,2-diol, which underwent oxidative cleavage (NaIO_4_/SiO_2_)[15] to afford **18** (83 %). The C21 tertiary alcohol stereocenter could then be configured with good selectivity (d.r. 10:1) through the Mukaiyama aldol addition[20] of silyl ketene acetal **19**, mediated by BF_3_⋅OEt_2_ at −78 °C. Subsequent acid-mediated cyclization then provided δ-lactone **20** (82 %, 2 steps).[21] In this situation, 1,2-induction by Felkin–Anh control and 1,3-induction based on the Evans polar model are mutually reinforcing.[22] The NMR spectroscopic data for **20** matched well with those for the δ-lactone ring of leiodermatolide, and the stereochemical assignment was further confirmed by single-crystal X-ray crystallography.[4b], [23] Silylation (TMSCl) then afforded **6** (97 %), in readiness for the key Heck reaction. After some experimentation, the exposure of **5** and **6** to Pd(OAc)_2_ (10 mol %) and Ag_2_CO_3_ in DMF at 80 °C[24] generated adduct **21** (73 %) with exclusively the required 16*E*,18*E* geometry. Stork–Wittig olefination[25] of the aldehyde arising from oxidative glycol cleavage of **21** (NaIO_4_/SiO_2_)[15] provided the *Z* vinyl iodide **3** (62 %, >20:1 *Z*/*E*), thus completing the synthesis of this fragment in 28 % yield over 10 steps.

**Scheme 3:**
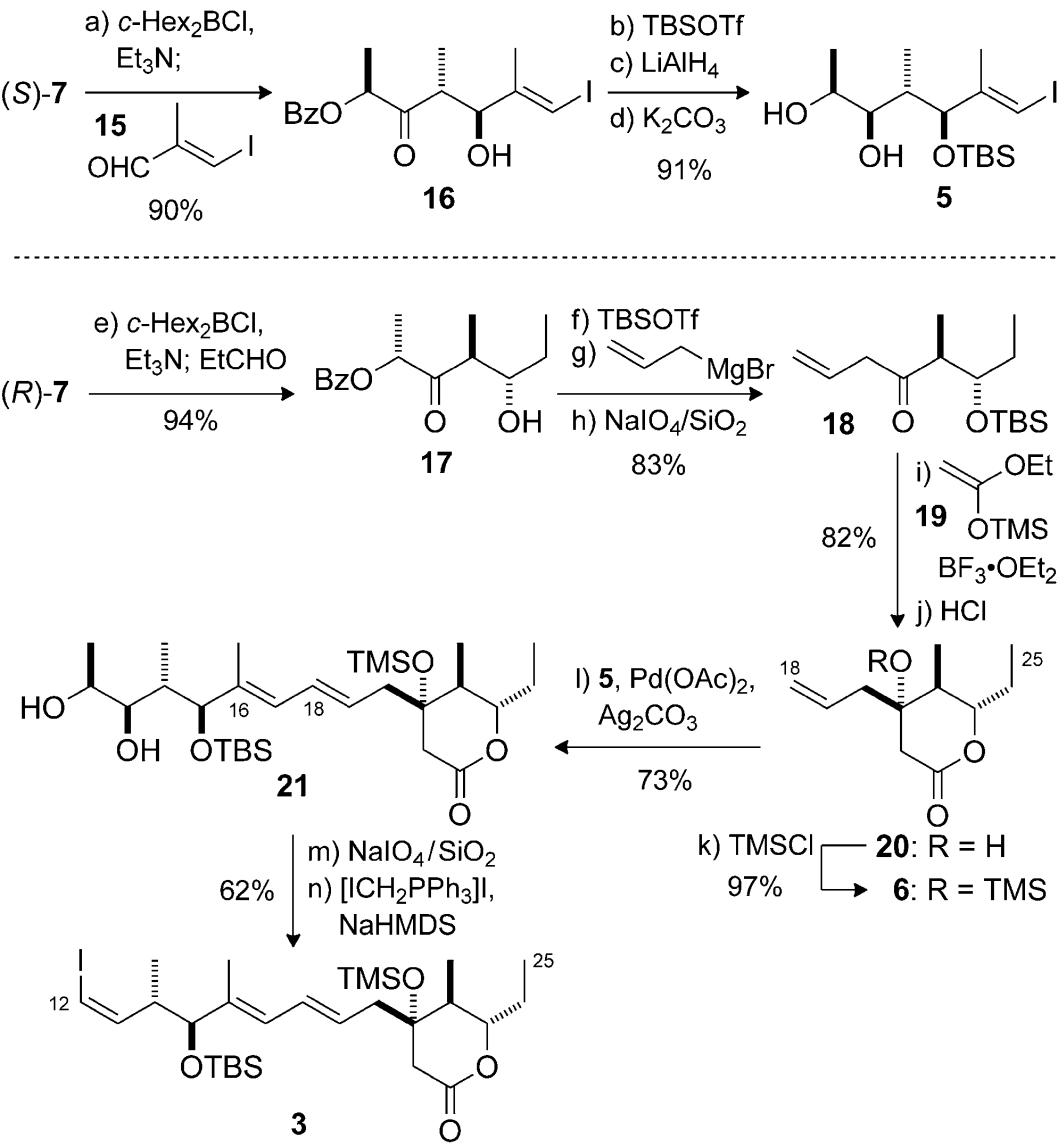
Preparation of vinyl iodide 3. Reagents and conditions: a) (*S*)-7, *c*-Hex_2_BCl, Et_3_N, Et_2_O, −78→−20 °C, 90 % (d.r.>20:1); b) TBSOTf, 2,6-lutidine, CH_2_Cl_2_, −78 °C; c) LiAlH_4_, THF, −78 °C; d) K_2_CO_3_, MeOH, 91 % over 3 steps; e) *c*-Hex_2_BCl, Et_3_N, Et_2_O; EtCHO, −78→−20 °C, 94 % (d.r.>20:1); f) TBSOTf, 2,6-lutidine, CH_2_Cl_2_, −78 °C, 98 %; g) H_2_C=CHCH_2_MgBr, THF, −78 °C; h) NaIO_4_, MeOH, pH 7 buffer, 85 % over 2 steps; i) 19, BF_3_⋅OEt_2_, CH_2_Cl_2_, −78 °C; j) 3 m HCl, THF, H_2_O, 82 % over 2 steps (d.r. 10:1); k) TMSCl, imid, CH_2_Cl_2_, 97 %; l) 5, Pd(OAc)_2_ (10 mol %), Ag_2_CO_3_, DMF, 80 °C, 73 %; m) NaIO_4_/SiO_2_, CH_2_Cl_2_; n) [ICH_2_PPh_3_]I, NaHMDS, THF, −78 °C, 62 % over 2 steps. DMF=*N*,*N*-dimethylformamide.

With an effective means of generating the two key fragments **2** and **3** secured, their controlled linkage to construct the full 25-carbon backbone of leiodermatolide was now executed (Scheme 4). Accordingly, Stille cross-coupling[26] of **2** and **3** under Fürstner conditions[26a] smoothly established the 10*Z*,12*Z* diene of **22** (80 %). A sequence of selective desilylation at C1 and C21 (HF⋅py, pyridine), oxidation at C1, and desilylation at C15 (TBAF) then provided the required seco acid **23** (51 % overall). Gratifyingly, Yamaguchi macrolactonization[27] served to efficiently close the 16-membered macrolactone. Acetonide cleavage then gave **24** (73 %), corresponding to the descarbamoyl derivative of leiodermatolide. Notably, the order of steps could be reversed, whereby acetonide cleavage was carried out first on **23** to give the unprotected tetraol, which was then macrolactonized to afford **24** with complete selectivity at C15.[23]

**Scheme 4:**
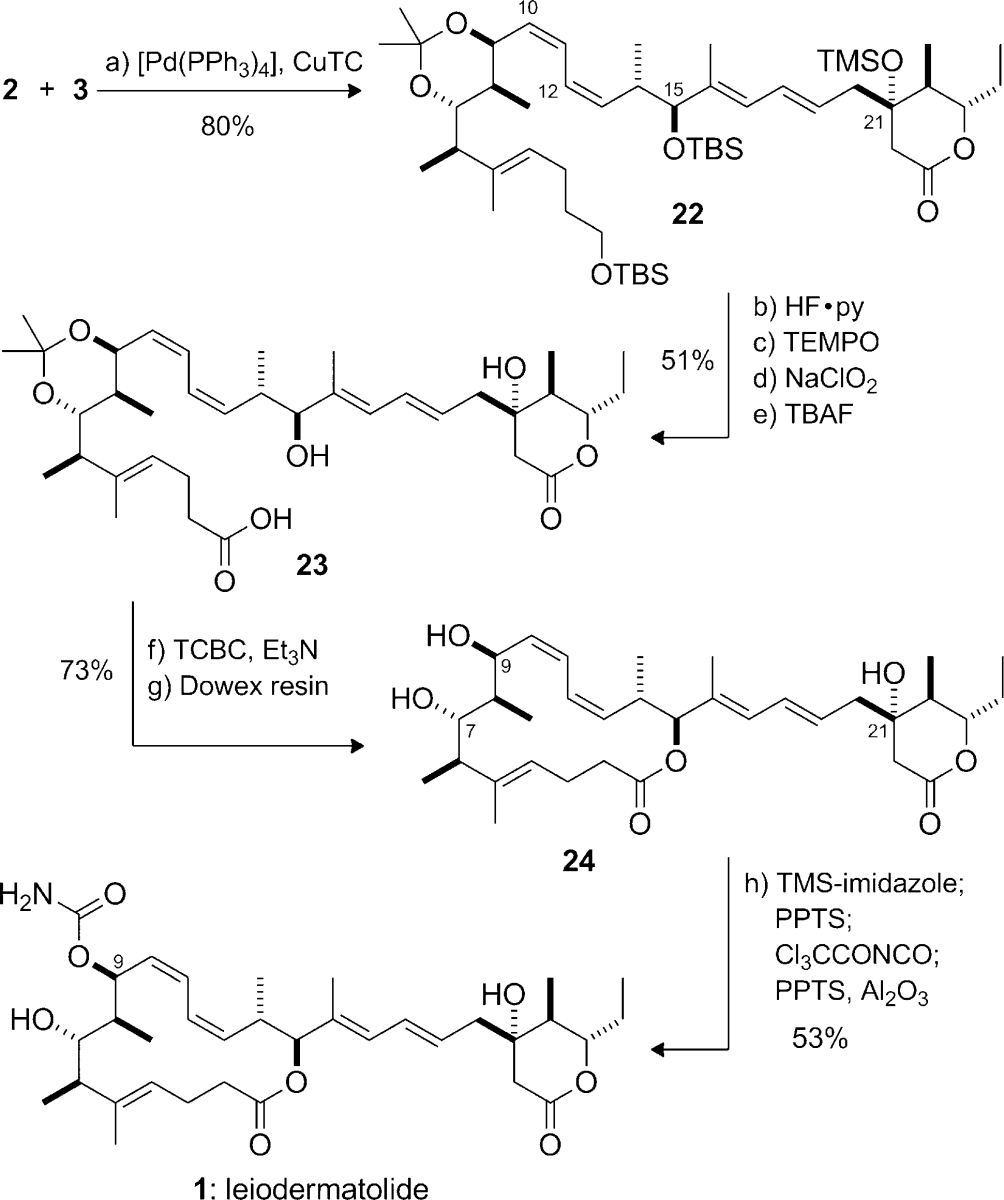
Completion of leiodermatolide (1). Reagents and conditions: a) [Pd(PPh_3_)_4_] (10 mol %), CuTC, Bu_4_NPh_2_PO_2_, DMF, 80 %; b) HF⋅py, pyridine, THF; c) TEMPO, PhI(OAc)_2_, CH_2_Cl_2_; d) NaClO_2_, NaH_2_PO_4_, 2-methyl-2-butene, *t*BuOH, H_2_O, THF; e) TBAF, THF, 50 °C, 51 % over 4 steps; f) TCBC, Et_3_N, THF; DMAP, PhMe, 80 %; g) Dowex 50WX8, MeOH, 91 %; h) TMS-imidazole, CH_2_Cl_2_; PPTS, MeOH; Cl_3_CCONCO, CH_2_Cl_2_, −78 °C; Al_2_O_3_; PPTS, MeOH, 53 %. DMAP=4-dimethylaminopyridine, py=pyridine, TBAF=tetrabutylammonium fluoride, TC=2-thiophenecarboxylate, TCBC=2,4,6-trichlorobenzoyl chloride, TEMPO=2,2,6,6-tetramethylpiperidine 1-oxyl.

Initially, we explored the introduction of the carbamate functionality on triol **24** itself by treatment with Cl_3_CCONCO (CH_2_Cl_2_, −78 °C),[28] but this reaction only afforded a disappointing 4:1 mixture of the C7 and C9 carbamates,[23], [29] a result anticipated from earlier studies with a truncated macrolide core.[5] To solve this problem, an effective sequence of hydroxy-group differentiation was sought to overturn the intrinsic substrate selectivity. Pleasingly, regiocontrolled silylation at C7 (1-(trimethylsilyl)imidazole; PPTS, MeOH) gave the corresponding C9/C21 diol, the treatment of which with Cl_3_CCONCO and acidic workup exclusively afforded (−)-leiodermatolide (**1**, 53 %; 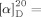
−74.0 (*c*=0.027, MeOH); lit.:[4] 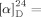
−84.2 (*c*=0.34, MeOH)). To our satisfaction, all ^1^H and ^13^C NMR spectroscopic data for this synthetic material correlated with those recorded for an authentic sample of natural leiodermatolide.

In conclusion, we have achieved a highly convergent total synthesis of the antimitotic marine macrolide (−)-leiodermatolide (**1**) in 23 steps and 3.2 % yield. This route features a uniformly high level of stereocontrol relying on lactate aldol chemistry,[9] combined with expedient fragment assembly based on a variety of palladium-catalyzed coupling reactions and an efficient macrolactonization step. It should be amenable to the synthesis of useful quantities of this otherwise scarce yet highly promising anticancer agent[30] for further biological evaluation and should also enable structure–activity-relationship studies. Indeed, we have already prepared the first novel leiodermatolide analogues in the form of triol **24** and the regioisomeric C7 carbamate.[31]
