# Supplementary material for: Total Synthesis of the Antimitotic Marine Macrolide (−)-Leiodermatolide
Source: Angew Chem Int Ed Engl. 2014 Jan 30;53(10):2692–5. doi: 10.1002/anie.201310164 (PMC4499261; doi:10.1002/anie.201310164)
Supplement: Supplementary file 1 [file anie0053-2692-sd1.pdf]

Angewandte Chemie
Eine Zeitschrift der Gesellschaft Deutscher Chemiker

Supporting Information
© Wiley-VCH 2014
69451 Weinheim, Germany

Total Synthesis of the Antimitotic Marine Macrolide (−)-Leiodermatolide**

Ian Paterson, Kenneth K.-H. Ng, Simon Williams, David C. Millican, and Stephen M. Dalby*

anie_201310164_sm_miscellaneous_information.pdf

Supporting information

Table of contents

I. General Procedures SI-2

II. Detailed experimental procedures

  a. Synthesis of western fragment **2** SI-4

  b. Synthesis of eastern fragment **3** SI-15

  c. Fragment coupling and endgame SI-25

III. Comparison of NMR data for natural and synthetic leiodermatolide SI-39

IV. ^{1}H and ^{13}C NMR spectra SI-40

I. General Procedures

Reactions were carried out under an atmosphere of argon using oven dried glassware and standard techniques for handling air sensitive chemicals, unless the reaction contained aqueous reagents or unless otherwise stated.

Reagents were purified using standard laboratory procedures,[1] toluene, benzene, dichloromethane, and acetonitrile were distilled from CaH_2 and stored under an atmosphere of argon. Tetrahydrofuran (THF) and Et_2O were distilled from potassium or sodium wire / benzophenone ketyl radical and stored under argon. Solvents used for extraction and chromatography were distilled. 2,6-lutidine, diisopropylamine, hexamethyldisilazane (HMDS), and triethylamine were distilled from CaH_2 and stored over CaH_2 under an atmosphere of argon. Acetic acid was distilled from acetic anhydride and CrO_3 and stored over 4Å molecular sieves. *N*,*N*-Dimethylformamide (DMF) was distilled from $MgSO_4$ and stored over 4Å molecular sieves. 2,3-Dichloro-5,6-dicyano-1,4-benzoquinone (DDQ) was recrystallised from C_6H_6 and $CHCl_3$ (1:5). All other chemicals were used as received from the manufacturer unless otherwise stated.

Aqueous solutions of ammonium chloride (NH_4Cl), sodium bicarbonate ($NaHCO_3$), sodium thiosulfate ($Na_2S_2O_3$), brine (NaCl) and sodium / potassium (Na/K) tartrate were saturated. Buffer solutions were prepared as directed from stock tablets.

Purification by flash column chromatography was carried out using Kieselgel 60 (230–400 mesh) and a positive pressure. Preparative thin layer chromatography was carried out using Merck Kieselgel 60 F254 plates.

TLC was carried out using Merck Kieselgel 60 F254 plates which were visualized using UV light (254 nm) and stained using potassium permanganate, anisaldehyde or phosphomolybdic acid / cerium sulfate dips.

NMR spectra were recorded using the following machines: Bruker Avance 500 cryo, Avance BB500, Avance TCI-ATM 500 cryo, and Avance DRX400. ^{1}H NMR spectra

[1] W. L. F. Armarego, D. D. Perrin, *Purification of Laboratory Chemicals*, 4th edition, Butterworth-Heinemann, **1996**.

were recorded at 298 K using the residual undeuterated solvent as a reference compound,[2] $CDCl_3$ (δ_H = 7.26) or CD_2Cl_2 (δ_H = 5.32). ^{1}H NMR data are presented as: chemical shift δ (in ppm, relative to tetramethylsilane, δ_{TMS} = 0), integration, multiplicity (s = singlet, d = doublet, t = triplet, q = quartet, m = multiplet, br = broad, obs = obscured) and coupling constants (J in Hz). Signals are assigned according to the numbering scheme for leiodermatolide Figure 1 unless otherwise indicated. Assignments have been made based on the 1D data presented along with a range of 2D spectra, and comparison with fully assigned spectra for similar compounds. ^{13}C NMR spectra were recorded at 298 K with proton decoupling and an internal deuterium lock for $CDCl_3$ (δ_C = 77.16) or CD_2Cl_2 (δ_C = 53.83).

Fourier transform IR spectroscopy (FT-IR) was carried out using a Perkin-Elmer Spectrum One spectrometer, and spectra were recorded as a thin film. Wavelengths of maximum absorption (ν_{max}) are reported in wavenumbers (cm^{-1}).

Optical rotations were measured using a Perkin-Elmer 241 polarimeter at the sodium D line (589 nm) and are reported as $[\alpha]_D^{20}$, concentration (c in g / 100 mL) and solvent.

High resolution mass spectroscopy (HRMS) was carried out by the EPSRC UK National Mass Spectrometry Facility (Swansea, UK) or the departmental Mass spectrometry service (University Chemical Laboratories, Cambridge) using electrospray ionization (ESI) or atmospheric pressure chemical ionization (APCI). The parent ion $[M+NH_4]^+$, $[M+Na]^+$ or $[M+H]^+$ is quoted.

The numbering system used for leiodermatolide follows that of Paterson and Wright[3] with the exception of methyl groups, which are denoted by the skeletal carbon they are appended to (Figure 1).

[2] G. R. Fulmer, A. J. M. Miller, N. H. Sherden, H. E. Gottlieb, A. Nudelman, B. M. Stoltz, J. E. Bercaw, K. I. Goldberg, *Organometallics*, **2010**, *29*, 2176.

[3] I. Paterson, S. M. Dalby, J. C. Roberts, G. J. Naylor, E. A. Guzmán, R. Isbrucker, T. P. Pitts, P. Linley, D. Divlianska, J. K. Reed, A. E. Wright, *Angew. Chem.* **2011**, *123*, 3277; *Angew. Chem. Int. Ed.* **2011**, *50*, 3219.

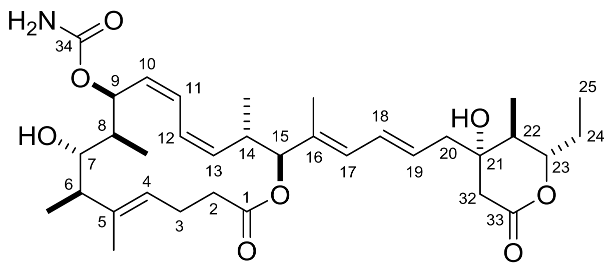

Figure 1. The numbering system for leiodermatolide (**1**)

II. Detailed experimental procedures

a. Synthesis of western fragment 2

Ketone **8a**

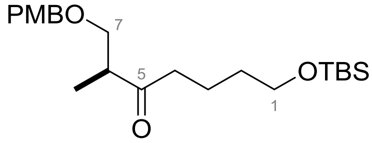

8a

To a suspension of magnesium turnings (1.10 g, 45.0 mmol) in THF (20 mL) was added iodine (2 crystals, approx. 20 mg) and 1,2-dibromoethane (50 μL) and the reaction stirred until it became colourless. A solution of TBSO$(CH_2)_4$Br[4] (11.5 g, 42.9 mmol) in THF (10 mL) was then added dropwise over 2 h and the reaction heated to reflux for 1 h to give a clear grey solution of the Grignard reagent.

Weinreb amide **8**[5] (4.58 g, 17.2 mmol, dried azeotropically with PhH) was dissolved in THF (25 mL) and cooled to −78 °C. The solution of Grignard reagent was added *via* cannula (25 mL THF wash) over 1 h, the reaction warmed to 0 °C and stirred for 1.5 h. NH_4Cl solution (10 mL) and water (30 mL) were added, the layers separated and the aqueous phase extracted with CH_2Cl_2 (4 × 100 mL). The combined organic extracts were dried ($MgSO_4$), concentrated *in vacuo* and purified by flash column chromatography (EtOAc / PE 40–60 1:20 → 1:4) to yield ketone **8a** as a colourless liquid (5.97 g, 15.1 mmol, 88%).

R$_f$ 0.6 (EtOAc / PE 40–60 1:20), **^{1}H NMR** (400 MHz, $CDCl_3$) δ_H 7.19 (2H, d, J = 8.7 Hz, ArH), 6.85 (2H, d, J = 8.7 Hz, ArH), 4.41 (1H, d, J = 11.6 Hz, OC$\underline{H_a}$H$_b$PMP), 4.37 (1H, d, J = 11.6 Hz, OCH$_a$$\underline{H_b}$PMP), 3.78 (3H, s, OMe), 3.58 (2H, t, J = 6.5 Hz, H1), 3.56 (1H, dd, J = 9.1, 7.8 Hz, H7a), 3.40 (1H, dd, J = 9.1, 5.5 Hz, H7b), 2.83

[4] P. Tauh, A. G. Fallis, *J. Org. Chem,* **1999**, *64*, 6960.
[5] I. Paterson, G. J. Florence, K. Gerlach, J. P. Scott, *J. Am. Chem. Soc.* **2001**, *123*, 9535.

(1H, dqd, J = 7.8, 7.1, 5.5 Hz, H6) 2.48 (2H, t, J = 7.3 Hz, H4), 1.60 (2H, tt, J = 7.3, 7.1 Hz, H3), 1.48 (2H, tt, J = 7.1, 6.5 Hz, H2), 1.03 (3H, d, J = 7.1 Hz, Me6), 0.87 (9H, s, Si<u>*t*-Bu</u>Me$_2$), 0.02 (6H, s, Si*t*-Bu<u>Me$_2$</u>); **^{13}C NMR** (125 MHz, CDCl$_3$) δ_C 213.1, 159.2, 130.2, 129.2, 113.8, 72.9, 72.0, 62.9, 55.2, 46.4, 41.8, 32.3, 26.0, 19.9, 18.3, 13.6, −5.3; **$[\alpha]_D^{20}$** +11.4 (*c* 1.1, CHCl$_3$); **IR** (thin film / cm^{-1}) 2931, 2857, 1714, 1613, 1586, 1523, 1463, 1361, 1302, 1248, 1173, 1094, 1037, 1006, 835, 755; **HRMS** (ESI) calc. for $C_{22}H_{39}O_4Si$ [M+H]$^+$ 395.2612, found 395.2613.

Vinyl triflate **4**

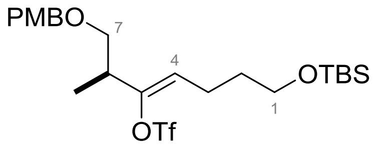

4

To a solution of hexamethyldisilazane (3.29 mL, 15.8 mmol) in THF (40 mL) at 0 °C was added *n*-butyllithium solution (1.50 M in hexanes, 8.42 mL, 12.6 mmol) and the solution stirred at 0 °C for 30 min before cooling to −78 °C. Ketone **8a** (4.16 g, 10.5 mmol) was dissolved in THF (40 mL), cooled to −78 °C and the cooled solution of LiHMDS added *via* cannula. The reaction was stirred at −78 °C for 1 h before addition of *N*-(5-chloro-2-pyridyl)triflimide (6.20 g, 15.8 mmol) in THF (40 mL) and stirred at −78 °C for a further 1 h before warming slowly to −30 °C over 2.5 h. Water (200 mL) was added, the layers separated and the aqueous phase extracted with EtOAc (3 × 100 mL). The combined organic extracts were dried (MgSO$_4$), concentrated *in vacuo* and purified by flash column chromatography (EtOAc / PE 40–60 1:50 → 1:20) to give vinyl triflate **4** as a colourless oil (4.54 g, 8.61 mmol, 82%).

R$_f$ 0.43 (EtOAc / PE 40–60 1:10); **^{1}H NMR** (500 MHz, CDCl$_3$) δ_H 7.25 (2H, d, J = 8.6 Hz, ArH), 6.90 (2H, d, J = 8.6 Hz, ArH), 5.36 (1H, t, J = 7.3 Hz, H4) 4.46 (2H, s, OC<u>H$_2$</u>PMP), 3.83 (3H, s, OMe), 3.63 (2H, t, J = 6.3 Hz, H1), 3.53 (1H, dd, J = 9.4, 5.3 Hz, H7a), 3.38 (1H, dd, J = 9.4, 6.4 Hz, H7b), 2.74 (1H, ddq, J = 6.8, 6.4, 5.3 Hz, H6), 2.27 (2H, dt, J = 7.3, 7.3 Hz, H3), 1.63 (2H, tt, J = 7.3, 6.3 Hz, H2), 1.19 (3H, d, J = 6.8 Hz, Me6), 0.91 (9H, s, Si<u>*t*-Bu</u>Me$_2$), 0.06 (6H, s, Si*t*-Bu<u>Me$_2$</u>); **^{13}C NMR** (125 MHz, CDCl$_3$) δ_C 159.2, 150.6, 130.1, 129.2, 128.3, 121.0, 118.6 (q, J = 319.5 Hz), 113.8, 72.7, 71.5, 62.3, 55.3, 38.4, 31.8, 25.9, 22.5, 18.3, 15.6, −5.4; **$[\alpha]_D^{20}$** +11.3 (*c*

1.2, $CHCl_3$); **IR** (thin film / cm^{-1}) 2933, 2858, 1614, 1514, 1409, 1247, 1208, 1142, 1097, 906, 834, 776; **HRMS** (ESI) calc. for $C_{23}H_{41}O_6NF_3SSi$ $[M+NH_4]^+$ 544.2370, found 544.2362.

Alkene **9**

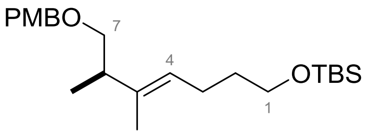

9

To a solution of vinyl triflate **4** (313 mg, 0.611 mmol) in 1,4-dioxane (10 mL) was added potassium carbonate (337 mg, 2.44 mmol), tetrakis(triphenylphosphine)palladium(0) (70.6 mg, 0.0611 mmol) and trimethylboroxine (0.128 mL, 0.920 mmol). The reaction was heated to 50 °C with stirring for 3 h before cooling to rt. The reaction mixture was filtered through a pad of Celite®, concentrated *in vacuo* and purified immediately by column chromatography (EtOAc / PE 40–60 1:20) to give alkene **9** as a colourless oil (224 mg, 0.572 mmol, 96%).

$\mathbf{R_f}$ 0.24 (EtOAc / PE 40–60 1:20); **^{1}H NMR** (500 MHz, $CDCl_3$) δ_H 7.25 (2H, d, *J* = 8.7 Hz, ArH), 6.87 (2H, d, *J* = 8.8 Hz, ArH), 5.20 (1H, t, *J* = 7.0 Hz, H4), 4.44 (2H, s, OC$\underline{H_2}$PMP), 3.80 (3H, s, OMe), 3.60 (2H, t, *J* = 6.5 Hz, H1), 3.41 (1H, dd, *J* =9.4, 6.8 Hz, H7a), 3.25 (1H, dd, *J* = 9.3, 7.3 Hz, H7b), 2.42 (1H, ddq, *J* = 6.9, 6.9, 6.9 Hz, H6), 2.05 (2H, dt, *J* = 7.3, 7.3 Hz, H3), 1.57 (2H, m obs, H2), 1.57 (3H, s, Me5), 1.01 (3H, d, *J* = 7.0 Hz, Me6), 0.90 (9H, s, Si$\underline{t\text{-Bu}}$Me$_2$), 0.05 (6H, s, Si*t*-Bu$\underline{Me_2}$); **^{13}C NMR** (125 MHz, $CDCl_3$) δ_C 159.2, 137.4, 131.0, 129.3, 124.6, 113.8, 74.0, 72.6, 62.8, 55.4, 42.6, 33.0, 26.1, 24.1, 18.5, 16.7, 13.6, −5.1; $[\alpha]_D^{20}$ −10.0 (*c* 1.0, $CHCl_3$); **IR** (thin film / cm^{-1}) 2952, 2931, 2858, 1613, 1514, 1463, 1249, 1097, 1039, 835; **HRMS** (ESI) calc. for $C_{23}H_{44}NO_3Si$ $[M+NH_4]^+$ 410.3085, found 410.3082.

Alcohol **9d**

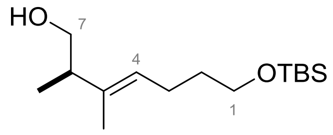

9d

To a rapidly stirred solution of PMB ether **9** (2.59 g, 6.59 mmol) in CH_2Cl_2 (36 mL) and pH 7 buffer (4 mL) at 0 °C was added DDQ (2.99 g, 13.2 mmol). The reaction was stirred at rt for 2 h, then quenched with $NaHCO_3$ solution (100 mL) and water (200 mL) until all the solids had dissolved. The mixture was extracted with CH_2Cl_2 (4 × 70 mL), the combined organic phases dried ($MgSO_4$) and concentrated *in vacuo*. The crude product was purified by column chromatography (CH_2Cl_2 / PhMe 1:10 → 1:2) to yield alcohol **9d** as a yellow oil (1.51 g, 5.54 mmol, 84%).

R$_f$ 0.13 (EtOAc / PE 40–60 1:20); **^{1}H NMR** (500 MHz, $CDCl_3$) δ_H 5.27 (1H, t, J = 7.1 Hz, H4), 3.60 (2H, t, J = 6.4 Hz, H1), 3.44 (2H, dd, J = 6.3, 6.3 Hz, H7), 2.31 (1H, ddq, J = 6.9, 6.9, 6.9 Hz, H6), 2.09 (2H, dt, J = 7.4, 7.4 Hz, H3), 1.56 (2H, m obs, H2), 1.56 (3H, s, Me5), 1.36 (1H, t, J = 6.0 Hz, OH), 0.98 (3H, d, J = 6.9 Hz, Me6), 0.89 (9H, s, Si*t*-BuMe$_2$), 0.04 (6H, s, Si*t*-BuMe$_2$); **^{13}C NMR** (125 MHz, $CDCl_3$) δ_C 136.2, 126.9, 65.3, 62.7, 45.4, 33.0, 26.1, 24.2, 18.5, 15.6, 12.7, −5.1; **$[\alpha]_D^{20}$** +5.0 (*c* 1.0, $CHCl_3$); **IR** (thin film / cm^{-1}) 3352, 2956, 2929, 2859, 1472, 1464, 1388, 1255, 1101, 1036; **HRMS** (ESI) calc. for $C_{15}H_{32}O_2Si$ $[M+H]^+$ 273.2244, found 273.2249.

Aldehyde **10**

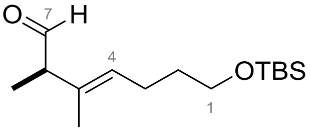

10

Alcohol **9d** (1.80 g, 6.64 mmol) was dissolved in CH_2Cl_2 (175 mL) and cooled to 0 °C. $NaHCO_3$ (5.60 g, 66.4 mmol) and Dess–Martin periodinane (4.20 g, 9.96 mmol) were added and the reaction stirred at rt for 45 min. The reaction was recooled to 0 °C, $Na_2S_2O_3$ (150 mL) was added and the mixture stirred for 30 min at rt. The layers were separated and the aqueous portion extracted with CH_2Cl_2 (4 × 100 mL), the combined organic extracts were dried ($MgSO_4$) and concentrated *in vacuo*. Purification by flash

column chromatography (EtOAc / PE 40–60 1:50) gave aldehyde **10** as a colourless liquid (1.48 g, 5.47 mmol, 83%).

R$_f$ 0.42 (EtOAc / PE 40–60 1:20); **^{1}H NMR** (500 MHz, $CDCl_3$) δ_H 9.49 (1H, s, H7), 5.31 (1H, t, *J* = 7.2 Hz, H4), 3.59 (2H, t, *J* = 6.4 Hz, H1), 2.95 (1H, q, *J* = 6.9 Hz, H6), 2.11 (2H, dt, *J* = 7.3, 7.3 Hz, H3), 1.58 (3H, s, Me5), 1.57 (2H, m obs, H2), 1.15 (3H, d, *J* = 6.9 Hz, Me6), 0.88 (9H, s, Si*t*-BuMe$_2$), 0.03 (6H, s, Si*t*-BuMe$_2$); **^{13}C NMR** (125 MHz, $CDCl_3$) δ_C 202.3, 131.9, 129.5, 62.5, 55.9, 32.7, 26.1, 24.5, 18.4, 14.8, 12.3, −5.2; **$[\alpha]_D^{20}$** −131.3 (*c* 1.3, $CHCl_3$); **IR** (thin film / cm^{-1}) 2952, 2930, 2858, 1727, 1472, 1462, 1387, 1255, 1101, 835; **HRMS** (ESI) calc. for $C_{15}H_{31}O_2Si$ $[M+H]^+$ 271.2088, found 271.2086.

Aldol adduct **11**

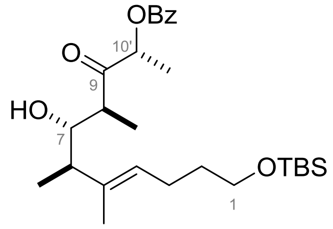

11

Ketone (*R*)-**7**[6] (1.02 g, 4.95 mmol) was dried azeotropically with PhH, dissolved in Et_2O (8 mL) and stirred over CaH_2 for 30 min. Dicyclohexylboron chloride (1.02 mL, 4.26 mmol) was added to a solution of triethylamine (0.646 mL, 4.86 mmol) in Et_2O (5 mL) at 0 °C and the clear solution stirred for 5 min. The solution of ketone was then added *via* cannula and the flask washed with Et_2O (3 mL); the cloudy solution was stirred at 0 °C for 1 h before cooling to −78 °C. Aldehyde **10** (821 mg, 3.04 mmol) was dried azeotropically from PhH, dissolved in Et_2O (5 mL) and stirred over CaH_2 for 30 min. This solution was added *via* cannula to the cooled enolate solution and the flask washed with Et_2O (3 mL). The reaction was stirred at −78 °C for 3 h and −20 °C for 14 h. MeOH (10 mL) and pH 7 buffer solution (10 mL) were added at 0 °C, the layers separated and the aqueous phase extracted with Et_2O (3 × 20 mL). The combined organic extracts were stirred over silica gel for 1 h, the silica was then removed by filtration and the solvent removed *in vacuo*. Purification by flash column chromatography (EtOAc / PE 40–60 1:50 → 1:10) gave aldol adduct **11** as a

[6] I. Paterson, D. J. Wallace, C. J. Cowden, *Synthesis* **1998**, 639.

colourless oil (1.39 g, 2.92 mmol, 96%) along with recovered ketone **7** (362 mg, 1.76 mmol).

R_f 0.48 (EtOAc / PE 40–60 1:4); **^{1}H NMR** (500 MHz, $CDCl_3$) δ_H 8.09 (2H, d, *J* = 7.2 Hz, ArH), 7.59 (1H, t, *J* = 7.4 Hz, ArH), 7.47 (2H, dd, *J* = 7.7, 7.7 Hz, ArH), 5.45 (1H, q, *J* = 6.9 Hz, H10′), 5.20 (1H, t, *J* = 7.1 Hz, H4), 3.59 (2H, t, *J* = 6.3 Hz, H1), 2.97 (1H, dq, *J* = 7.1, 7.1 Hz, H8), 2.29 (1H, d, *J* = 5.5 Hz, OH), 2.27 (1H, m obs, H6), 2.10 (2H, m, H3), 1.60 (3H, s, Me5), 1.55 (3H, d, *J* = 7.4 Hz, Me10′), 1.55 (2H, m, H2), 1.29 (3H, d, *J* = 7.1 Hz, Me8), 1.05 (3H, d, *J* = 6.9 Hz, Me6), 0.90 (9H, s, Si*t*-BuMe$_2$), 0.05 (6H, s, Si*t*-BuMe$_2$); **^{13}C NMR** (125 MHz, $CDCl_3$) δ_C 212.1, 165.7, 137.3, 133.3, 129.8, 129.6, 128.4, 126.3, 74.9, 74.7, 62.6, 44.8, 44.5, 32.8, 26.0, 24.1, 18.3, 15.9, 14.9, 14.5, 12.9, −5.3; $[\alpha]_D^{20}$ −13.7 (*c* 1.1, $CHCl_3$); **IR** (thin film / cm^{-1}) 3519, 2952, 2932, 2856, 1719, 1453, 1381, 1268, 1108, 1100; **HRMS** (ESI) calc. for $C_{27}H_{45}O_5Si$ $[M+H]^+$ 477.3031, found 477.3025.

TMS Ether **11g**

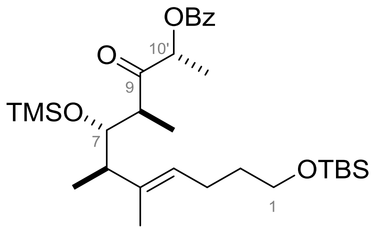

11g

Aldol adduct **11** (1.39 g, 2.92 mmol) was dissolved in CH_2Cl_2 (30 mL) and cooled to 0 °C, imidazole (594 mg, 8.73 mmol) then chlorotrimethylsilane (0.93 mL, 7.30 mmol) were added and the reaction stirred at rt for 30 min. The reaction was quenched with NH_4Cl solution (20 mL), the layers separated and the aqueous phase extracted with CH_2Cl_2 (3 × 20 mL). The combined organic extracts were dried ($MgSO_4$) and concentrated *in vacuo* to give TMS ether **11g** as a colourless oil (1.53 g, 2.79 mmol, 96%) which was used without further purification.

R_f 0.60 (EtOAc / PE 40–60 1:9); **^{1}H NMR** (500 MHz, $CDCl_3$) δ_H 8.07 (2H, dt, *J* = 8.3, 1.2 Hz, ArH), 7.56 (1H, tt, *J* = 7.5, 1.2 Hz, ArH), 7.44 (2H, dd, *J* = 7.8, 7.8 Hz, ArH), 5.43 (1H, q, *J* = 7.0 Hz, H10′), 5.14 (1H, t, *J* = 7.0 Hz, H4), 3.99 (1H, dd, *J* = 8.7, 2.7 Hz, H7), 3.59 (2H, t, *J* = 6.5 Hz, H1), 3.07 (1H, dq, *J* = 8.1, 7.3 Hz, H8), 2.15 (1H, q,

J = 6.3 Hz, H6), 2.08 (1H, ddt, J = 15.0, 7.6, 7.6 Hz, H3a), 1.98 (1H, m, H3b), 1.61 (3H, s, Me5), 1.56 (2H, m, H2), 1.51 (3H, d, J = 6.9 Hz, Me10′), 1.15 (3H, d, J = 7.0 Hz, Me8), 0.95 (3H, d, J = 6.9 Hz, Me6), 0.89 (9H, s, Si*t*-BuMe$_2$), 0.04 (6H, s, Si*t*-BuMe$_2$), −0.03 (9H, s, SiMe$_3$); **^{13}C NMR** (125 MHz, CDCl$_3$) δ_C 209.3, 165.8, 136.5, 133.3, 129.92, 129.90, 128.5, 125.7, 75.6, 75.1, 62.9, 47.0, 43.5, 32.9, 26.1, 24.5, 18.4, 16.7, 15.5, 14.8, 12.1, 0.7, −5.2; $[\alpha]_D^{20}$ +8.9 (*c* 1.3, CHCl$_3$); **IR** (thin film / cm^{-1}) 2594, 2932, 2858, 1723, 1452, 1379, 1264, 1249, 1109, 837; **HRMS** (ESI) calc. for $C_{30}H_{56}O_5NSi$ $[M+NH_4]^+$ 566.3692, found 566.3687.

Triol **11i**

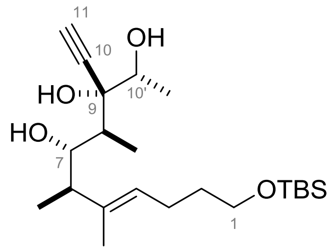

11i

n-Butyllithium solution (1.6 M in hexanes, 11.6 mL, 18.6 mmol) was added dropwise to a solution of ethynyltrimethylsilane (3.29 mL, 23.3 mmol) in THF (22 mL) at 0 °C and stirred for 20 min. The reaction was cooled to −78 °C and a solution of ketone **11g** (1.28 g, 2.33 mmol) in THF (6.0 mL) was added *via* cannula. The reaction was stirred at −78 °C for 1 h then warmed to 0 °C and stirred for a further 4 h. NH_4Cl solution (40 mL) was added, the layers separated and the aqueous phase extracted with Et_2O (2 × 20 mL) and EtOAc (1 × 20 mL). The combined organic extracts were dried (Na_2SO_4) and concentrated *in vacuo*. The residue was dissolved in MeOH (20 mL), K_2CO_3 (4.83 g, 35.0 mmol) was added and the reaction stirred for 16 h at rt. NH_4Cl solution (20 mL) was added and the aquesous phase extracted with EtOAc (3 × 20 mL). The combined organic extracts were dried (Na_2SO_4), concentrated *in vacuo* and purified by column chromatography (EtOAc / PE 40–60 1:4 → 1:1) to give triol **11i** as a colourless oil (714 mg, 1.79 mmol, 77%).[7]

R_f 0.35 (EtOAc / PE 40–60 1:2); **^{1}H NMR** (500 MHz, CDCl$_3$) δ_H 5.47 (1H, s, OH9), 5.28 (1H, t, J = 6.7 Hz, H4), 3.95 (1H, d, J = 9.5 Hz, H7), 3.88 (1H, dq, J = 8.5, 6.2

[7] The (inconsequential) stereochemistry at C9 was confirmed by nOe analysis of an orthoacetate derivative of **11i**.

Hz, H10′), 3.61 (2H, t, *J* = 6.2 Hz, H1), 2.66 (1H, d, *J* = 8.8 Hz, OH10′), 2.48 (1H, s, H11), 2.43 (1H, br s, OH7), 2.35 (1H, m, H6), 2.13 (2H, m, H3), 1.80 (1H, dq, *J* = 9.7, 6.8 Hz, H8), 1.70 (3H, s, Me5), 1.58 (2H, tt, *J* = 6.8, 6.8 Hz, H2), 1.23 (3H, d, *J* = 6.2 Hz, Me10′), 0.97 (3H, d, *J* = 6.9 Hz, Me6), 0.91 (3H, d, *J* = 6.5 Hz, Me8), 0.89 (9H, s, Si<u>*t*-Bu</u>Me$_2$), 0.04 (6H, s, Si*t*-Bu<u>Me$_2$</u>); **^{13}C NMR** (125 MHz, CDCl$_3$) δ_C 137.1, 126.6, 84.8, 77.2, 73.7, 73.3, 72.2, 62.6, 42.8, 40.5, 32.8, 26.0, 24.4, 18.4, 17.1, 16.1, 11.6, 10.1, −5.1, −5.3; **$[\alpha]_D^{20}$** +11.6 (*c* 2.0, CHCl$_3$); **IR** (thin film / cm^{-1}) 3310, 2928, 2859, 1721, 1427, 1256, 1103, 984, 836, 775; **HRMS** (ESI) calc. for $C_{22}H_{43}O_4Si$ $[M+H]^+$ 399.2925, found 399.2927.

Ynone **12**

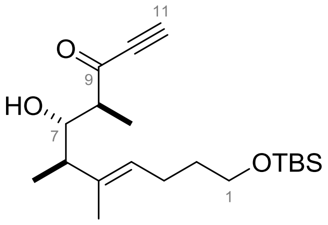

12

Triol **11i** (714 mg, 1.79 mmol) was dissolved in CH_2Cl_2 (25 mL), silica supported sodium periodate (14.6 wt% $NaIO_4$, 10.5 g, 7.16 mmol) was added and the reaction stirred for 1 h. The mixture was filtered, concentrated *in vacuo* to give ynone **12** as a yellow oil (570 mg, 1.62 mmol, 90%) which was used without further purification.

R_f 0.50 (EtOAc / PE 40–60 1:4); **^{1}H NMR** (400 MHz, CDCl$_3$) δ_H 5.22 (1H, t, *J* = 7.1 Hz, H4), 3.63 (1H, ddd, *J* = 6.2, 6.2, 6.2 Hz, H7), 3.59 (2H, t, *J* = 6.4 Hz, H1), 3.26 (1H, s, H11), 2.79 (1H, qd, *J* = 7.2, 6.4 Hz, H8), 2.30 (1H, dq, *J* = 6.7, 6.7 Hz, H6), 2.14 (1H, d, *J* = 6.4 Hz, OH), 2.06 (2H, dt, *J* = 7.4, 7.4 Hz, H3), 1.59 (3H, s, Me5), 1.55 (2H, tt, *J* = 7.0, 7.0 Hz, H2), 1.25 (3H, d, *J* = 7.3 Hz, Me8), 1.04 (3H, d, *J* = 6.9 Hz, Me6), 0.88 (9H, s, Si<u>*t*-Bu</u>Me$_2$), 0.03 (6H, s, Si*t*-Bu<u>Me$_2$</u>); **^{13}C NMR** (100 MHz, CDCl$_3$) δ_C 191.7, 137.2, 127.1, 81.4, 79.6, 75.2, 63.0, 51.1, 45.7, 33.1, 26.4, 24.5, 18.7, 14.8, 14.3, 13.7, −4.9; **$[\alpha]_D^{20}$** −24.9 (*c* 1.0, CHCl$_3$); **IR** (thin film / cm^{-1}) 3464, 3246, 2952, 2930, 2857, 2091, 1674, 1462, 1255, 1098; **HRMS** (ESI) calc. for $C_{20}H_{36}O_3SiNa$ $[M+Na]^+$ 375.2326, found 375.2327.

Iodoenone **13**

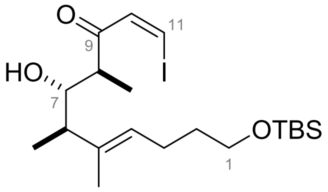

13

Ynone **12** (364 mg, 1.03 mmol) was dissolved in THF (1.72 mL), AcOH (103 μL, 1.80 mmol) and sodium iodide (309 mg, 2.06 mmol) were added and the reaction was stirred in the dark at rt for 40 h. $NaHCO_3$ solution (2.0 mL) was added, the mixture was extracted with CH_2Cl_2 (3 × 5 mL), dried (Na_2SO_4) and concentrated *in vacuo*. Purification by flash column chromatography (EtOAc / PE 40–60 1:50 → 1:18) gave the *Z*-iodoenone **13** as a yellow oil (398 mg, 828 μmol, 81%) along with the *E*- isomer (41.4 mg, 86.2 μmol, 8%).

R_f 0.38 (EtOAc / PE 40–60 1:10); **^{1}H NMR** (500 MHz, $CDCl_3$) δ_H 7.32 (1H, d, *J* = 8.8 Hz, H11), 7.23 (1H, d, *J* = 8.8 Hz, H10), 5.14 (1H, t, *J* = 7.1 Hz, H4), 3.58 (2H, t, *J* = 6.4 Hz, H1), 3.51 (1H, ddd, *J* = 7.5, 7.5, 5.1 Hz, H7), 2.59 (1H, d, *J* = 7.8 Hz, OH), 2.27 (1H, dq, *J* = 7.0, 7.0 Hz, H6), 2.02 (1H, dt, *J* = 7.4, 7.4 Hz, H3), 1.55 (3H, s, Me5), 1.52 (2H, tt, *J* = 7.1, 7.1 Hz, H2), 1.21 (3H, d, *J* = 7.2 Hz, Me8), 1.08 (3H, d, *J* = 7.0 Hz, Me6), 0.89 (9H, s, Si<u>*t*-Bu</u>Me_2), 0.04 (6H, s, Si*t*-Bu<u>Me_2</u>); **^{13}C NMR** (125 MHz, $CDCl_3$) δ_C 204.3, 137.3, 135.2, 126.4, 91.7, 76.5, 62.6, 48.2, 46.7, 32.8, 26.1, 24.1, 18.3, 14.41, 14.36, 13.6, −5.3; **$[\alpha]_D^{20}$** +41.1 (*c* 1.3, $CHCl_3$); **IR** (thin film / cm^{-1}) 3497, 2930, 2857, 1684, 1565, 1460, 1256, 1102, 975, 836 776; **HRMS** (ESI) calc. for $C_{20}H_{38}IO_3Si$ $[M+H]^+$ 481.1629, found 481.1627.

Diol **14**

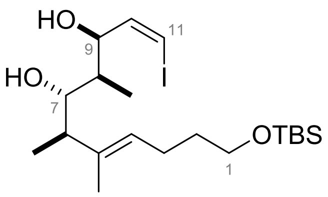

14

$Me_4NBH(OAc)_3$ (1.88 g, 7.08 mmol) was dissolved in AcOH (8.0 mL) and MeCN (8.0 mL) and the solution stirred at 0 °C for 1 h before cooling to −30 °C. A solution of ketone **13** (340 mg, 708 μmol) in MeCN (16 mL) was added *via* cannula and the

reaction stirred at −30 °C in the dark for 40 h. The mixture was pipetted into $NaHCO_3$ and Na/K tartrate solution (1:1, 20 mL) at 0 °C and stirred at rt for 30 min. The layers were separated and the aqueous phase extracted with EtOAc (4 × 20 mL), the combined organic extracts were washed with $NaHCO_3$ solution (10 mL), dried (Na_2SO_4) and concentrated *in vacuo*. Residual boronate was removed by co-evaporation with MeOH (4 × 2 mL) and the crude product was purified by flash column chromatography (EtOAc / PE 40–60 1:9 → 1:4) to give diol **14** as a colourless oil (330 mg, 684 μmol, 97%).

R_f 0.59 (EtOAc / PE 40–60 1:4), **^{1}H NMR** (500 MHz, $CDCl_3$) δ_H 6.39 (2H, m, H10, H11), 5.31 (1H, t, *J* = 7.2 Hz, H4), 4.63 (1H, dt, *J* = 2.0, 6.1 Hz, H9), 3.60 (3H, m, H1, OH), 3.56 (1H, m, H7), 2.36 (1H, dq, *J* = 6.5, 6.5 Hz, H6), 2.09 (1H, dt, *J* = 7.3, 7.3 Hz, H3), 1.99 (1H, dqd, *J* = 7.2, 7.2, 2.5 Hz, H8), 1.62 (3H, s, Me5), 1.57 (2H, tt, *J* = 7.2, 7.2 Hz, H2), 1.03 (3H, d, *J* = 7.0 Hz, Me6), 0.97 (3H, d, *J* = 7.2 Hz, Me8), 0.89 (9H, s, Si*t*-BuMe₂), 0.04 (6H, s, Si*t*-BuMe₂); **^{13}C NMR** (125 MHz, $CDCl_3$) δ_C 141.2, 137.1, 126.5, 82.7, 76.5, 75.0, 62.6, 44.2, 39.3, 32.8, 26.0, 24.3, 18.4, 15.7, 12.6, 12.2, −5.3; **$[\alpha]_D^{20}$** +20.5 (*c* 0.9, CH_2Cl_2); **IR** (thin film / cm^{-1}) 3354, 2929, 2342, 1462, 1255, 1103, 967, 836, 775; **HRMS** (ESI) calc. for $C_{20}H_{39}O_3INaSi$ $[M+Na]^+$ 505.1605, found 505.1600.

Acetonide **14m**

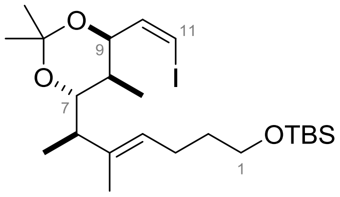

14m

Diol **14** (27 mg, 56.0 μmol) was dissolved in 2,2-dimethoxypropane (1 mL) and CH_2Cl_2 (2 mL) and pyridinium *para*-toluenesulfonate (several crystals) was added. The reaction was stirred at rt for 1.5 h, concentrated *in vacuo* and purified by flash column chromatography (EtOAc / PE 40–60 1:12) to give acetonide **14m** as a colourless oil (29.1 mg, 55.6 μmol, 99%).

R_f 0.55 (EtOAc / PE 40–60 1:10), **^{1}H NMR** (500 MHz, $CDCl_3$) δ_H 6.35 (1H, d, *J* = 7.4 Hz, H11), 6.30 (1H, dd, *J* = 7.4, 7.4 Hz, H10), 5.27 (1H, t, *J* = 7.3 Hz, H4), 4.61

(1H, dd, J = 7.1, 4.9 Hz, H9), 3.60 (3H, t, J = 6.6 Hz, H1), 3.27 (1H, dd, J = 6.8, 6.8 Hz, H7), 2.21 (1H, dq, J = 6.8, 6.8 Hz, H6), 2.03 (3H, m, H8, H3), 1.58 (3H, s, Me5), 1.56 (2H, m obs, H2), 1.40 (3H, s, acetonide), 1.34 (3H, s, acetonide), 1.07 (3H, d, J = 6.9 Hz, Me6), 0.89 (9H, s, Si*t*-BuMe_2), 0.81 (3H, d, J = 6.8 Hz, Me8), 0.04 (6H, s, Si*t*-Bu$\underline{Me_2}$); **^{13}C NMR** (125 MHz, $CDCl_3$) δ_C 139.8, 137.0, 126.0, 100.5, 81.7, 76.9, 73.4, 62.7, 47.2, 37.1, 32.8, 26.0, 25.7, 24.0, 23.8, 18.4, 14.9, 13.7, 13.1, −5.2; $[\alpha]_D^{20}$ +16.6 (*c* 0.30, $CHCl_3$); **IR** (thin film / cm^{-1}) 2930, 2859, 1461, 1378, 1226, 1106, 1017, 836, 774; **HRMS** (ESI) calc. for $C_{23}H_{44}O_3ISi$ $[M+H]^+$ 523.2099, found 523.2094.

Vinyl stannane **2**

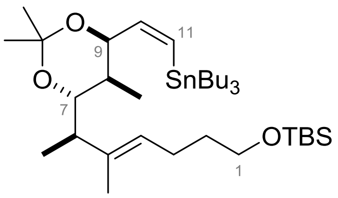

2

Tributyltin chloride (36 μL, 133 μmol) was added to a solution of vinyl iodide **14m** (17.4 mg, 33.0 μmol) in Et_2O (2 mL) and the solution cooled to −78 °C. *t*-Butyllithium solution (1.7 M in pentane, 116 μL, 198 μmol) was added dropwise and the reaction stirred for 30 min before being quenched with NH_4Cl solution (2.5 mL) and immediately warmed to rt. The layers were separated and the aqueous phase extracted with CH_2Cl_2 (3 × 3 mL), the combined organic extracts were dried ($MgSO_4$) and concentrated *in vacuo*. Purification by flash column chromatography (Et_3N washed silica gel, EtOAc / PE 40–60 0:1 → 1:10) gave vinyl stannane **2** as a colourless oil (18.3 mg, 26.7 μmol, 81%).

R_f 0.9 (EtOAc / PE 40–60 1:20); **^{1}H NMR** (500 MHz, $CDCl_3$) δ_H 6.42* (1H, dd, J = 13.4, 6.6 Hz, H10), 5.97* (1H, dd, J = 13.4, 0.9 Hz, H11), 5.23 (1H, t, J = 7.0 Hz, H4), 4.27 (1H, ddd, J = 6.7, 4.2, 0.9 Hz, H9), 3.59 (3H, t, J = 6.5 Hz, H1), 3.20 (1H, dd, J = 7.0, 7.0 Hz, H7), 2.14 (1H, qd, J = 7.0, 7.0 Hz, H6), 2.03 (2H, dt, J = 7.3, 7.3 Hz, H3), 1.79 (1H, dqd, J = 7.1, 7.1, 5.0 Hz, H8), 1.56 (3H, s, Me5), 1.57–1.46 (8H, m, H2, Sn$\underline{Bu_3}$), 1.34 (6H, s, acetonide), 1.31 (6H, m, Sn$\underline{Bu_3}$), 1.04 (3H, d, J = 6.8 Hz, Me6), 0.93–0.87 (24H, m, Sn$\underline{Bu_3}$, Si*t*-BuMe$_2$), 0.77 (3H, d, J = 6.9 Hz, Me8), 0.04

* These peaks show satellites due to coupling to ^{117}Sn and ^{119}Sn.

(6H, s, Si*t*-BuMe$_2$); **^{13}C NMR** (125 MHz, $CDCl_3$) δ_C 145.7, 137.1, 129.1, 125.8, 100.5, 76.9, 73.0, 62.7, 47.5, 39.6, 32.8, 29.2, 27.4, 26.0, 25.7, 24.0, 23.8, 18.4, 14.9, 13.74, 13.70, 13.1, 10.9, −5.3; **$[\alpha]_D^{20}$** +13.8 (*c* 0.18, $CHCl_3$); **IR** (thin film / cm^{-1}) 2958, 2931, 2858, 1464, 1378, 1256, 1225, 1104, 836, 775; **HRMS** (ESI) calc. for $C_{35}H_{74}NO_3SiSn$ $[M+NH_4]^+$ 701.4476, found 701.4124.

b. Synthesis of eastern fragment 3

Aldol adduct **16**

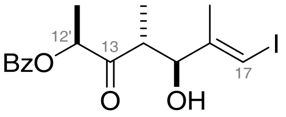

16

A solution of ketone (*S*)-**7**[6] (3.46 g, 16.8 mmol, dried azeotropically with PhH) in Et_2O (5.0 mL) was dried over CaH_2 for 5 min. In a separate flask, dicyclohexylboron chloride (3.43 mL, 15.7 mmol) was added to a solution of triethylamine (2.81 mL, 20.1 mmol) in Et_2O (10 mL) at 0 °C. The solution of ketone (*S*)-**7** was then added *via* cannula (2 × 2.5 mL Et_2O wash) to the clear mixture and the resultant cloudy suspension was stirred at 0 °C for 1 h before cooling to −78 °C. Freshly prepared aldehyde **15**[8] (*ca.* 1.64 g, 8.39 mmol) in Et_2O (*ca.* 20 mL) was added *via* cannula (4 × 5.0 mL Et_2O wash) and the reaction stirred at −78 °C for 6 h before warming to −20 °C for 10 h. MeOH (20 mL) and pH 7 buffer solution (20 mL) were added at 0 °C, the layers separated and the aqueous phase was extracted with Et_2O (4 × 50 mL). The combined organic extracts were stirred over silica gel for 30 min, filtered, dried (Na_2SO_4) and concentrated *in vacuo*. Purification by flash column chromatography (EtOAc / PE 40–60 0:1 → 1:7) gave aldol adduct **16** as a white crystalline solid (3.04 g, 7.55 mmol, 90%).

R_f 0.26 (EtOAc / PE 40–60 1:4); **Melting point** 126–127 °C; **^{1}H NMR** (500 MHz, $CDCl_3$) δ_H 8.08 (2H, d, *J* = 7.2 Hz, ArH), 7.59 (1H, t, *J* = 7.4 Hz, ArH), 7.46 (2H, dd, *J* = 7.7, 7.7 Hz, ArH), 6.34 (1H, s, H17), 5.43 (1H, q, *J* = 7.0 Hz, H12′), 4.41 (1H, dd, *J* = 8.9, 4.4 Hz, H15), 3.05 (1H, dq, *J* = 8.8, 7.2 Hz, H14), 2.30 (1H, d, *J* = 4.4 Hz, OH), 1.82 (3H, s, Me16), 1.57 (3H, d, *J* = 7.0 Hz, Me12′), 1.08 (3H, d, *J* = 7.1 Hz,

[8] a) R. Baker, J. L. Castro, *J. Chem. Soc., Perkin Trans. 1* **1990**, 47; b) J. D. White, P. R. Blakemore, N. J. Green, E. B. Hauser, M. A. Holoboski, L. E. Keown, C. S. Nylund Kolz, B. W. Phillips, *J. Org. Chem.* **2002**, *67*, 7750.

Me14); **^{13}C NMR** (100 MHz, $CDCl_3$) δ_C 210.5, 166.0, 147.5, 133.5, 129.9, 129.5, 128.6, 81.2, 78.8, 75.0, 45.6, 18.9, 15.6, 14.5; **$[\alpha]_D^{20}$** +40.3 (*c* 1.0, $CHCl_3$); **IR** (thin film / cm^{-1}) 3499, 2992, 2939, 1733, 1699, 1451, 1385, 1317, 1286, 1271; **HRMS** (ESI) calc. for $C_{16}H_{20}O_4I$ $[M+H]^+$ 403.0401, found 403.0402.

TBS ether **16b**

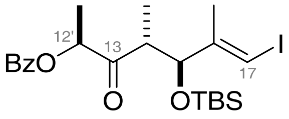

16b

2,6-Lutidine (2.20 mL, 18.9 mmol) and TBSOTf (2.17 mL, 9.43 mmol) were added to a solution of aldol adduct **16** (1.27 g, 3.15 mmol) in CH_2Cl_2 (15 mL) at −78 °C. After stirring at −78 °C for 2 h, further 2,6-lutidine (2.20 mL, 18.9 mmol) and TBSOTf (2.17 mL, 9.43 mmol) were added. After stirring at −78 °C for a further 1h, the reaction was quenched by the addition of MeOH (20 mL) followed by $NaHCO_3$ solution (40 mL). Upon warming up to rt, the layers were separated and the aqueous phase was extracted with CH_2Cl_2 (3 × 50 mL). The combined organic extracts were dried ($MgSO_4$) and concentrated *in vacuo*. Purification by flash column chromatography (EtOAc / PE 40–60 0:1 → 1:7) gave TBS ether **16b** as a colourless liquid (1.62 g, 3.13 mmol, 99%).

R_f 0.53 (EtOAc / PE 40–60 1:4); **^{1}H NMR** (500 MHz, $CDCl_3$) δ_H 8.07 (2H, d, *J* = 7.4 Hz, ArH), 7.58 (1H, t, *J* = 7.4 Hz, ArH), 7.45 (2H, dd, *J* = 7.7, 7.7 Hz, ArH), 6.26 (1H, s, H17), 5.42 (1H, q, *J* = 7.0 Hz, H12′), 4.44 (1H, d, *J* = 9.7 Hz, H15), 3.00 (1H, dq, *J* = 9.4, 7.2 Hz, H14), 1.77 (3H, s, Me16), 1.54 (3H, d, *J* = 7.0 Hz, Me12′), 0.96 (3H, d, *J* = 7.1 Hz, Me14), 0.81 (9H, s, Si$\underline{t\text{-Bu}}$Me$_2$), −0.02 (3H, s, Si*t*-Bu$\underline{\text{Me}_a}$Me$_b$), −0.04 (3H, s, Si*t*-BuMe$_a$$\underline{\text{Me}_b}$); **^{13}C NMR** (125 MHz, $CDCl_3$) δ_C 208.5, 165.8, 147.9, 133.4, 129.9, 129.7, 128.6, 80.7, 79.7, 75.2, 46.6, 25.8, 18.6, 18.2, 15.4, 14.4, −5.0, −5.1; **$[\alpha]_D^{20}$** +6.1 (*c* 1.0, $CHCl_3$); **IR** (thin film / cm^{-1}) 2957, 2931, 2858, 1722, 1604, 1452, 1266, 1116, 1070, 1000, 855, 836, 778, 771; **HRMS** (ESI) calc. for $C_{22}H_{34}O_4ISi$ $[M+H]^+$ 517.1266, found 517.1263.

Benzoate **16c**

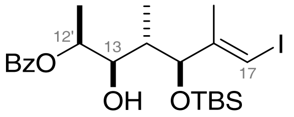

16c

Lithium aluminium hydride (237 mg, 6.25 mmol) was added to a solution of TBS protected aldol adduct **16b** (1.62 g, 3.13 mmol) in Et_2O (5 mL) at −78 °C. After stirring at −78 °C for 30 min, the reaction was quenched by the addition of acetone (5 mL) followed by NH_4Cl solution (5 mL). Upon warming up to rt, the layers were separated and the aqueous portion extracted with Et_2O (3 × 50 mL). The combined organic extracts were dried (Na_2SO_4) and concentrated *in vacuo* to give benzoate **16c** as a colourless liquid (1.60 g, 3.08 mmol, crude 98%, >20:1 dr at C13) to be used without further purification.

A portion was further purified by flash column chromatography (EtOAc/PE 40–60 0:1 → 1:20) to provide a sample for characterisation:
$\mathbf{R_f}$ 0.45 (EtOAc / PE 40–60 1:4); **^{1}H NMR** (400 MHz, $CDCl_3$) δ_H 8.07 (2H, d, J = 8.2 Hz, ArH), 7.55 (1H, t, J = 7.4 Hz, ArH), 7.43 (2H, dd, J = 7.6, 7.6 Hz, ArH), 6.25 (1H, s, H17), 5.28 (1H, qd, J = 6.3, 3.0 Hz, H12′), 4.22 (1H, d, J = 8.2 Hz, H15), 3.83 (1H, td, J = 8.0, 2.4 Hz, H13), 3.80 (1H, s, OH), 1.80 (1H, m obs, H14), 1.78 (3H, s, Me16), 1.37 (3H, d, J = 6.4 Hz, Me12′), 0.90 (9H, s, Si<u>*t*-Bu</u>Me_2), 0.81 (3H, d, J = 7.0 Hz, Me14), 0.10 (3H, s, Si*t*-Bu<u>Me_a</u>Me_b), 0.02 (3H, s, Si*t*-BuMe_a<u>Me_b</u>); **^{13}C NMR** (100 MHz, $CDCl_3$) δ_C 166.3, 148.3, 133.0, 130.7, 129.8, 128.4, 83.4, 81.0, 75.8, 72.8, 40.1, 25.9, 19.7, 18.2, 13.3, 13.0, −4.6, −5.2; **$[\alpha]_D^{20}$** +5.1 (*c* 1.0, $CHCl_3$); **IR** (thin film / cm^{-1}) 3506, 2956, 2931, 2858, 1715, 1452, 1275, 1069, 1027, 835; **HRMS** (ESI) calc. for $C_{22}H_{36}O_4ISi$ $[M+H]^+$ 519.1422, found 519.1412.

Diol **5**

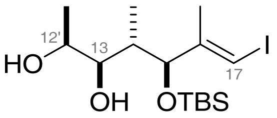

5

K_2CO_3 (869 mg, 6.25 mmol) was added to a solution of ester **16c** (1.60 g, 3.08 mmol) in MeOH (30 mL). The reaction was allowed to stir at rt for 2 h before it was quenched by the addition of NH_4Cl (10 mL) at 0 °C. The layers were separated and

the aqueous phase was extracted with CH_2Cl_2 (3 × 50 mL). The combined organic extracts were dried (Na_2SO_4) and concentrated *in vacuo*. Purification by flash column chromatography (EtOAc / PE 40–60 0:1 → 1:7) gave diol **5** as a colourless oil (1.19 g, 2.89 mmol, 92% over 2 steps).

R_f 0.26 (EtOAc / PE 40–60 1:4); **^{1}H NMR** (500 MHz, $CDCl_3$) δ_H 6.24 (1H, s, H17), 4.14 (1H, d, *J* = 8.6 Hz, H15), 3.91 (1H, dd, *J* = 1.7, 0.6 Hz, OH13), 3.79 (1H, dqdd, *J* = 9.1, 6.3, 3.5, 0.6 Hz, H12′), 3.63 (1H, ddd, *J* = 8.2, 3.5, 1.8 Hz, H13), 2.51 (1H, d, *J* = 9.1 Hz, OH12′), 1.79 (3H, d, *J* = 1.1 Hz, Me16), 1.71 (1H, tq, *J* = 8.4, 7.0 Hz, H14), 1.16 (3H, d, *J* = 6.4 Hz, Me12′), 0.91 (9H, s, Si<u>*t*-Bu</u>Me_2), 0.68 (3H, d, *J* = 6.9 Hz, Me14), 0.12 (3H, s, Si*t*-Bu$\underline{Me_a}Me_b$), 0.03 (3H, s, Si*t*-Bu$Me_a\underline{Me_b}$); **^{13}C NMR** (125 MHz, $CDCl_3$) δ_C 148.4, 84.1, 80.9, 77.6, 68.5, 39.4, 25.9, 19.5, 18.2, 16.3, 12.7, −4.5, −5.2; $[\alpha]_D^{20}$ +36.5 (*c* 1.0, $CHCl_3$); **IR** (thin film / cm^{-1}) 3442, 2954, 2930, 2857, 1615, 1472, 1379, 1258, 1072, 1040, 837, 777; **HRMS** (ESI) calc. for $C_{15}H_{31}O_4INaSi$ $[M+Na]^+$ 437.0979, found 437.0979.

Ketone **18**

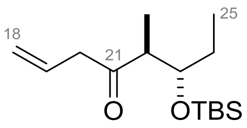

18

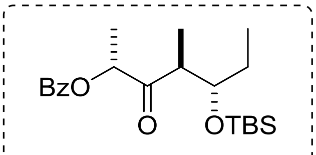

17f

Allylmagnesium bromide solution (1 M in Et_2O, 19.5 mL, 19.5 mmol) was added dropwise to a stirred solution of TBS ether **17f**[9] (2.10 g, 5.56 mmol) in THF (25 mL) at −78 °C over 5 min. The reaction was stirred for 1.5 h before being quenched at −78 °C with NH_4Cl (10 mL) and warmed to rt. The organic phase was separated, the aqueous phase extracted with Et_2O (3 × 30 mL), and the combined organic extracts concentrated *in vacuo*. The crude diol was dissolved in MeOH / pH 7 buffer (3:1, 20 mL), $NaIO_4$ (3.57 g, 16.7 mmol) added, and the reaction mixture stirred at rt for 16 h. H_2O (20 mL) was added and the MeOH removed *in vacuo*. The mixture was extracted with CH_2Cl_2 (3 × 30 mL) and the combined organic extracts were dried ($MgSO_4$) and concentrated *in vacuo*. Purification by flash column chromatography (EtOAc / PE 40–60 1:30) afforded ketone **18** (1.27 g, 4.70 mmol, 85% over 2 steps) as a pale yellow oil.

[9] S. Crossman, M. V. Perkins, *J. Org. Chem.* **2006**, *71*, 117.

R$_f$ 0.56 (EtOAc / PE 40–60 1:10); **^{1}H NMR** (500 MHz, $CDCl_3$) δ_H 5.93 (1H, ddt, *J* = 17.1, 10.2, 6.9 Hz, H19), 5.16 (1H, d, *J* = 10.2 Hz, H18a), 5.10 (1H, d, *J* = 17.1 Hz, H18b), 3.90 (1H, dt, *J* = 7.7, 4.3 Hz, H23), 3.27–3.23 (2H, m, H20), 2.80 (1H, dq, *J* = 7.2, 7.2 Hz, H22), 1.48 (2H, m, H24), 0.97 (3H, d, *J* = 7.0 Hz, Me22), 0.89 (3H, t, *J* = 7.3 Hz, H25), 0.86 (9H, s, Si*t*-BuMe$_2$), 0.04 (3H, s, Si*t*-BuMe$_a$Me$_b$), −0.01 (3H, s, Si*t*-BuMe$_a$Me$_b$); **^{13}C NMR** (125 MHz, $CDCl_3$) δ_C 211.9, 130.9, 118.6, 74.7, 49.9, 48.7, 26.3, 26.0, 18.2, 12.8, 7.8, −4.4, −4.7; **$[\alpha]_D^{20}$** +65.6 (*c* 1.9, $CHCl_3$); **IR** (thin film / cm^{-1}) 2958, 2932, 2858, 1719, 1463, 1256, 1122, 1071, 1040, 1005, 836, 776; **HRMS** (ESI) calc. for $C_{15}H_{30}SiO_2Na$ $[M+Na]^+$ 293.1907, found 293.1893.

Aldol adduct **18i**

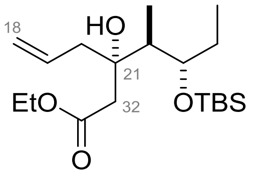

18i

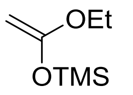

19

To a stirred solution of ketone **18** (500 mg, 1.85 mmol) in CH_2Cl_2 (10 mL) at −78 °C was added silyl ketene acetal **19** (600 μL, 3.70 mmol), followed by $BF_3{\cdot}Et_2O$ (350 μL, 2.78 mmol). The solution was stirred at −78 °C for 1.5 h before being quenched with $NaHCO_3$ (5 mL) and warmed to rt. The organic phase was separated, and the aqueous phase extracted with CH_2Cl_2 (3 × 10 mL). The combined organic extracts were dried ($MgSO_4$) and concentrated *in vacuo*. Purification by flash column chromatography (EtOAc / PE 40–60 1:30 → 1:20) afforded ester **18i** (577 mg, 1.61 mmol, 87%) as a colourless oil.

Alternatively, the reaction was carried out with ketone **18** (881 mg, 3.26 mmol) and silyl ketene acetal **19** (2.61 mL, 16.3 mmol) in CH_2Cl_2 (15 mL) under the same conditions and the crude product was used in the subsequent step without further purification.

R$_f$ 0.39 (EtOAc / PE 40–60 1:15); **^{1}H NMR** (500 MHz, $CDCl_3$) δ_H 5.95 (1H, m, H19), 5.11 (1H, d, *J* = 10.0 Hz, H18a), 5.10 (1H, d, *J* = 17.3 Hz, H18b), 4.55 (1H, s, OH), 4.14 (2H, q, *J* = 7.1 Hz, CH_3CH_2O), 3.94 (1H, ddd, *J* = 5.6, 5.6, 4.7 Hz, H23), 2.61

(1H, d, J = 14.2 Hz, H32a), 2.50 (1H, dd, J = 14.5, 6.3 Hz, H20a), 2.44 (1H, d, J = 14.2 Hz, H32b), 2.38 (1H, dd, J = 14.4, 8.0 Hz, H20b), 1.93 (1H, dq, J = 7.0, 7.0 Hz, H22), 1.65 (1H, m, H24a), 1.55 (1H, m, H24b), 1.26 (3H, t, J = 7.1 Hz, C$\underline{H}_3$CH$_2$O), 0.91 (9H, s, Si$\underline{t\text{-Bu}}$Me$_2$), 0.89 (3H, obs, H25), 0.83 (3H, d, J = 7.1 Hz, Me22), 0.13 (3H, s, Si*t*-Bu$\underline{\text{Me}_a}$Me$_b$), 0.11 (3H, s, Si*t*-BuMe$_a\underline{\text{Me}_b}$); **^{13}C NMR** (125 MHz, CDCl$_3$) δ_C 172.6, 134.3, 117.9, 75.9, 75.4, 60.6, 43.2, 43.0, 41.5, 26.6, 26.1, 18.2, 14.3, 11.1, 8.5, −3.8, −4.5; **$[\alpha]_D^{20}$** −9.0 (*c* 2.3, CHCl$_3$); **IR** (thin film / cm^{-1}) 3486, 2957, 2932, 2858, 1733, 1714, 1464, 1373, 1336, 1255, 1191, 1055, 1005, 836, 774; **HRMS** (ESI) calc. for $C_{19}H_{38}SiO_4Na$ [M+Na]$^+$ 381.2432, found 381.2433.

Lactone **20**

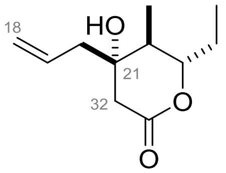

20

To a stirred solution of ester **18i** (274 mg, 765 μmol) in THF / MeOH (3:2, 5.0 mL) at rt was added HCl (3 M aq., 2.0 mL). The mixture was stirred for 1.5 h, then cooled to 0 °C and quenched with $NaHCO_3$ (3 mL). The organic phase was separated, and the aqueous phase extracted with CH_2Cl_2 (3 × 10 mL). The combined organic extracts were dried ($MgSO_4$) and concentrated *in vacuo*. Purification by flash column chromatography (EtOAc / PE 40–60 1:10 → 1:3) afforded lactone **20**[10] (142 mg, 717 μmol, 94%) as a white crystalline solid.

Alternatively, the reaction was carried out with crude aldol adduct **18f** in THF (9.0 mL) and MeOH (6.0 mL) under the same conditions and the crude product was used in the subsequent step without further purification.

R$_f$ 0.21 (EtOAc / PE 40–60 1:2); **Melting point** 56–58 °C; **^{1}H NMR** (500 MHz, CDCl$_3$) δ_H 5.86 (1H, dddd, J = 17.2, 10.1, 7.4, 7.4 Hz, H19), 5.28 (1H, d, J = 10.1 Hz,

[10] Previously reported NMR data were recorded with C_6D_6 as a solvent and our data recorded in C_6D_6 were consistent, see: J. Willwacher, N. Kausch-Busies, A. Fürstner, *Angew. Chem.* **2012**, *124*, 12207; *Angew. Chem. Int. Ed.* **2012**, *51*, 12041.
The enantiomeric lactone *ent*-**20** was also prepared analogously from (*S*)-**7** with identical spectroscopic data with the exception of $[\alpha]_D^{20}$ = −5.4 (*c* 3.09, CHCl$_3$).

H18a), 5.20 (1H, d, J = 17.1 Hz, H18b), 3.94 (1H, ddd, J = 10.0, 7.3, 2.9 Hz, H23), 2.78 (1H, d, J = 16.7 Hz, H26a), 2.38 (1H, d, J = 16.9 Hz, H26b), 2.33 (1H, dd, J = 13.9, 7.5 Hz, H20a), 2.16 (1H, dd, J = 13.8, 7.2 Hz, H20b), 2.14 (1H, obs, OH), 1.92 (1H, dq, J = 10.0, 6.9 Hz, H22), 1.85 (1H, dqd, J = 14.8, 7.3, 3.1 Hz, H24a), 1.62 (1H, ddq, J = 14.6, 7.3, 7.3 Hz, H24b), 1.02 (3H, d, J = 7.0 Hz, Me22), 1.01 (3H, d, J = 7.1 Hz, H25); **^{13}C NMR** (125 MHz, $CDCl_3$) δ_C 170.5, 131.6, 121.5, 83.8, 71.2, 42.8, 42.7, 39.0, 26.9, 11.3, 9.0; **$[\alpha]_D^{20}$** +5.8 (c 1.17, $CHCl_3$); **IR** (thin film / cm^{-1}) 3432, 2976, 2942, 1721, 1641, 1464, 1378, 1248, 1045, 1007, 920; **HRMS** (ESI) calc. for $C_{11}H_{18}O_3Na$ $[M+Na]^+$ 221.1148, found 221.1147.

X-ray crystallographic analysis confirmed the relative configuration of lactone **20** as shown in Figure 2.[11]

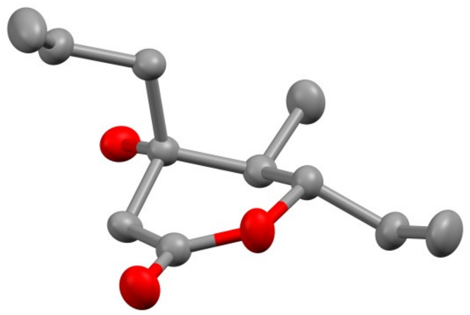

Figure 2 ORTEP drawing of lactone **20** with thermal ellipsoids shown at 50% probability level. Hydrogen atoms are omitted for clarity.

Lactone **6**

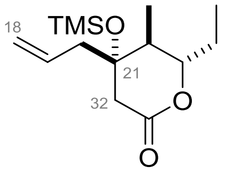

6

Imidazole (90.7 mg, 1.33 mmol) and TMSCl (135 µL, 1.07 mmol) were added to a solution of alcohol **20** (176 mg, 0.888 mmol) in CH_2Cl_2 (9 mL) at 0 °C. The reaction was stirred for 1 h before NH_4Cl solution was added. The layers were separated and the aqueous phase extracted with CH_2Cl_2 (3 × 10 mL). The combined organic extracts

[11] CCDC-782549 containing the supplementary crystallographic data can be obtained free of charge *via* http://www.ccdc.cam.ac.uk/Community/Requestastructure/ or from the Cambridge Crystallographic Data Centre, 12, Union Road, Cambridge, CB2 1EZ, UK; fax: (+44) 1223-336-033; e-mail: deposit@ccdc.cam.ac.uk.

were dried (Na_2SO_4), concentrated *in vacuo* and purified by flash column chromatography (EtOAc / PE 40–60 1:50 → 1:20) to give lactone **6** (233 mg, 0.862 mmol, 97%) as white needle like crystals.

Alternatively, the reaction was carried out with crude lactone **20**, imidazole (655 mg, 9.77 mmol) and TMSCl (827 μL, 6.52 mmol) in CH_2Cl_2 (8 mL) under the same conditions. Purification provided lactone **6** as white needle like crystals (682 mg, 2.52 mmol, 77% over 3 steps).

R_f 0.45 (EtOAc / PE 40–60 1:10); **Melting point** 33–34 °C; **^{1}H NMR** (500 MHz, $CDCl_3$) δ_H 5.82 (1H, dddd, J = 17.0, 10.1, 8.4, 5.8 Hz, H19), 5.15 (1H, d, J = 10.1 Hz, H18a), 5.09 (1H, d, J = 17.2 Hz, H18b), 3.94 (1H, ddd, J = 10.3, 7.4, 3.1 Hz, H23), 2.88 (1H, d, J = 16.3 Hz, H32a), 2.43 (1H, d, J = 16.5 Hz, H32b), 2.38 (1H, dd, J = 14.0, 5.7 Hz, H20a), 2.04 (1H, dd, J = 14.0, 8.1 Hz, H20b), 1.91–1.80 (2H, m, H22, H24a), 1.58 (1H, ddq, J = 14.6, 7.3, 7.3 Hz, H24b), 1.02 (3H, t, J = 7.3 Hz, H25), 1.02 (3H, d, J = 6.8 Hz, Me22), 0.14 (9H, s, $SiMe_3$); **^{13}C NMR** (125 MHz, $CDCl_3$) δ_C 170.2, 133.0, 119.2, 83.9, 74.8, 43.9, 43.5, 39.9, 27.0, 11.1, 9.1, 2.5; **$[\alpha]_D^{20}$** +30.7 (*c* 0.7, $CHCl_3$); **IR** (thin film / cm^{-1}) 2962, 1742, 1251, 1077, 997, 841, 756; **HRMS** (ESI) calc. for $C_{14}H_{27}O_3Si$ $[M+H]^+$ 271.1724, found 271.1753.

Diene **21**

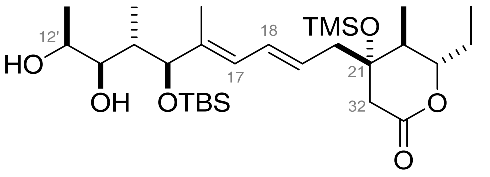

21

$Pd(OAc)_2$ (21.5 mg, 95.8 μmol) and Ag_2CO_3 (264 mg, 0.958 mmol) was added to a degassed solution of vinyl iodide **5** (512 mg, 1.15 mmol) and lactone **6** (259 mg, 0.958 mmol) in DMF (2.5 mL). The mixture was heated to 80 °C for 10 h before cooled to rt, diluted with Et_2O, and filtered through a plug of Celite®. The crude product was concentrated *in vacuo* and purified by column chromatography (EtOAc / PE 40–60 1:50 → 1:20) to give diene **21** as a colourless liquid (389 mg, 0.699 mmol, 73%).

R_f 0.20 (EtOAc / PE 40–60 3:7); **^{1}H NMR** (400 MHz, $CDCl_3$) δ_H 6.23 (1H, dd, *J* = 15.1, 10.8 Hz, H18), 5.90 (1H, d, *J* = 10.7 Hz, H17), 5.65 (1H, ddd, *J* = 15.0, 7.8, 6.9 Hz, H19), 4.25 (1H, s, OH13), 3.94 (1H, d, *J* = 8.7 Hz, H15), 3.92 (1H, ddd, *J* = 10.5, 7.4, 3.0 Hz, H23), 3.76 (1H, m, H12′), 3.64 (1H, dd, *J* = 8.0, 3.0 Hz, H13), 2.89 (1H, d, *J* = 16.4 Hz, H32a), 2.60 (1H, br s, OH12), 2.42 (1H, d, *J* = 16.2 Hz, H32b), 2.40 (1H, m obs, H20a), 2.08 (1H, dd, *J* = 14.1, 8.0 Hz, H20b), 1.80–1.89 (2H, m, H22, H24a), 1.68 (3H, s, Me16), 1.68 (1H, obs, H14), 1.56 (1H, ddq, *J* = 14.6, 7.3, 7.3 Hz, H24b), 1.15 (3H, d, *J* = 6.3 Hz, Me12′), 1.00 (3H, d, *J* = 6.8 Hz, Me23), 1.00 (3H, t, *J* = 7.3 Hz, H25), 0.89 (9H, s, Si<u>*t*-Bu</u>Me$_2$), 0.64 (3H, d, *J* = 6.9 Hz, Me14), 0.11 (9H, s, $SiMe_3$), 0.10 (3H, s, Si*t*-Bu<u>Me$_a$</u>Me$_b$), 0.00 (3H, s, Si*t*-BuMe$_a$<u>Me$_b$</u>); **^{13}C NMR** (125 MHz, $CDCl_3$) δ_C 170.0, 136.6, 129.9, 128.8, 128.2, 85.7, 83.7, 78.0, 75.0, 68.5, 44.0, 43.5, 39.0, 38.6, 26.9, 25.9, 18.1, 16.3, 12.8, 11.8, 11.0, 9.0, 2.5, −4.2, −5.2; **$[\alpha]_D^{20}$** +8.9 (*c* 1.03, $CHCl_3$); **IR** (thin film / cm^{-1}) 3449, 2955, 2932, 2856, 1736, 1463, 1377, 1251, 1075, 1004, 840; **HRMS** (ESI) calc. for $C_{29}H_{57}O_6Si_2$ $[M+H]^+$ 557.3688, found 557.3686.

Aldehyde **21m**

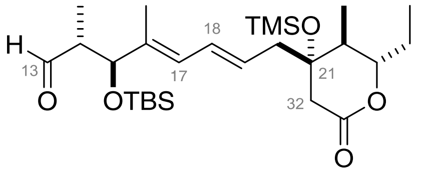

21m

Silica-supported $NaIO_4$ (14.6 wt% $NaIO_4$, 3.47 g, 2.37 mmol) was added to a solution of diol **21** (330 mg, 0.593 mmol) in CH_2Cl_2 (12 mL). The mixture was filtered after 1 h and concentrated in vacuo to give aldehyde 21m as a colourless liquid (300 mg, 0.587 mmol, crude 99%) which was used immediately without further purification.

A portion was further purified by flash column chromatography (EtOAc / PE 40–60 0:1 → 1:20) to provide a sample for characterisation:

R_f 0.45 (EtOAc / PE 40–60 1:4); **^{1}H NMR** (400 MHz, $CDCl_3$) δ_H 9.73 (1H, d, *J* = 2.8 Hz, H13), 6.25 (1H, dd, *J* = 15.1, 10.8 Hz, H18), 5.96 (1H, d, *J* = 10.8 Hz, H17), 5.65 (1H, ddd, *J* = 15.0, 8.1, 6.9 Hz, H19), 4.09 (1H, d, *J* = 8.4 Hz, H15), 3.91 (1H, ddd, *J* = 10.4, 7.4, 3.0 Hz, H23), 2.85 (1H, d, *J* = 16.4 Hz, H32a), 2.56 (1H, dqd, *J* = 8.2, 7.1, 2.9 Hz, H14), 2.42 (1H, d, *J* = 16.4 Hz, H32b), 2.40 (1H, m obs, H20a), 2.09 (1H, dd,

J = 14.1, 8.1 Hz, H20b), 1.77–1.89 (2H, m, H22, H24a), 1.69 (3H, s, Me16), 1.56 (1H, ddq, *J* = 14.6, 7.3, 7.3 Hz, H24b), 1.00 (3H, d, *J* = 6.8 Hz, Me23), 1.00 (3H, t, *J* = 7.3 Hz, H25), 0.85 (3H, m obs, Me14), 0.84 (9H, s, Si*t*-BuMe$_2$), 0.11 (9H, s, SiMe$_3$), 0.01 (3H, s, Si*t*-BuMe$_a$Me$_b$), −0.05 (3H, s, Si*t*-BuMe$_a$Me$_b$); **^{13}C NMR** (125 MHz, CDCl$_3$) δ_C 205.0, 170.0, 136.0, 130.0, 128.9, 127.6, 83.8, 80.3, 75.0, 50.4, 44.1, 43.6, 38.6, 27.0, 25.8, 18.2, 11.7, 11.07, 11.06, 9.0, 2.5, −4.4, −5.2; **$[\alpha]_D^{20}$** +15.3 (*c* 0.99, $CHCl_3$); **IR** (thin film / cm^{-1}) 2958, 2932, 2861, 1732, 1462, 1388, 1251, 1105, 1059, 840; **HRMS** (ESI) calc. for $C_{27}H_{54}O_5NSi_2$ $[M+NH_4]^+$ 528.3535, found 528.3531.

Vinyl iodide **3**

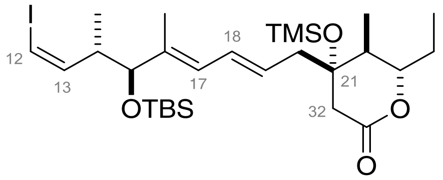

3

NaHMDS (1.0 M in THF, 1.79 mL. 1.79 mmol) was added to a suspension of $[PPh_3CH_2I]^+I^-$ (948 mg, 1.79 mmol) in THF (12 mL). After stirring at rt for 30 min, the mixture was cooled to −78 °C and a solution of crude aldehyde (300 mg, 0.587 mmol) in THF (4 mL) was added to the mixture *via* cannula (2 × 4 mL THF wash). The reaction was stirred at −78 °C for 3 h before quenched with hexanes (5 mL) and NH_4Cl solution (5 mL), and diluted with H_2O (10 mL). The phases were separated and the aqueous phase was extracted with Et_2O (3 × 50 mL). The combined organic extracts were dried ($MgSO_4$) and concentrated *in vacuo*. Purification by flash column chromatography (EtOAc / PE 40–60 0:1 → 1:20) afforded vinyl iodide **3** (232 mg, 0.365 mmol, 62% over 2 steps) as a colourless liquid.

R_f 0.85 (EtOAc / PE 40–60 1:20); **^{1}H NMR** (400 MHz, CDCl$_3$) δ_H 6.26 (1H, dd, *J* = 15.1, 10.9 Hz, H18), 6.14 (1H, d, *J* = 7.4 Hz, H12), 6.03 (1H, dd, 8.8, 7.4 Hz, H13), 5.92 (1H, d, *J* = 10.8 Hz, H17), 5.59 (1H, ddd, *J* = 15.0, 7.5, 7.5 Hz, H19), 3.93 (1H, ddd, *J* = 10.4, 7.4, 3.0 Hz, H23), 3.88 (1H, d, *J* = 5.8 Hz, H15), 2.88 (1H, d, *J* = 16.4 Hz, H32a), 2.71 (1H, m, H14), 2.44 (1H, d, *J* = 16.4 Hz, H32b), 2.38 (1H, m obs, H20a), 2.11 (1H, dd, *J* = 14.1, 8.0 Hz, H20b), 1.91–1.78 (2H, m, H22, H24a), 1.70 (3H, s, Me16), 1.57 (1H, ddq, *J* = 14.6, 7.3, 7.3 Hz, H24b), 1.02 (3H, t, *J* = 7.3 Hz, H25), 1.01 (3H, d, *J* = 6.8 Hz, Me23), 0.90 (3H, d, *J* = 7.1 Hz, Me14), 0.88 (9H, s,

Sit-BuMe$_2$), 0.12 (9H, s, SiMe$_3$), 0.02 (3H, s, Sit-BuMe$_a$Me$_b$), −0.05 (3H, s, Sit-BuMe$_a$Me$_b$); **^{13}C NMR** (100 MHz, CDCl$_3$) δ_C 170.3, 143.9, 138.0, 130.5, 127.4, 125.9, 83.7, 81.9, 81.0, 75.1, 44.4, 44.2, 43.6, 38.8, 27.0, 26.0, 18.3, 16.6, 13.0, 11.1, 9.1, 2.6, −4.5, −4.9; **$[\alpha]_D^{20}$** +51.1 (*c* 1.35, CHCl$_3$); **IR** (thin film / cm^{-1}) 2958, 2856, 1741, 1462, 1375, 1251, 1215, 1075, 839; **HRMS** (ESI) calc. for $C_{28}H_{55}O_4NSi_2$ $[M+NH_4]^+$ 652.2709, found 652.2706.

c. Fragment coupling and endgame

In the main text, the route to compound **22** is *via* acetonide protection of vinyl iodide **14**, stannylation and Stille coupling. It was also found to be possible to prepare **22** by stannylating compound **14** directly followed by Stille coupling and acetonide formation as described below (Scheme 1).

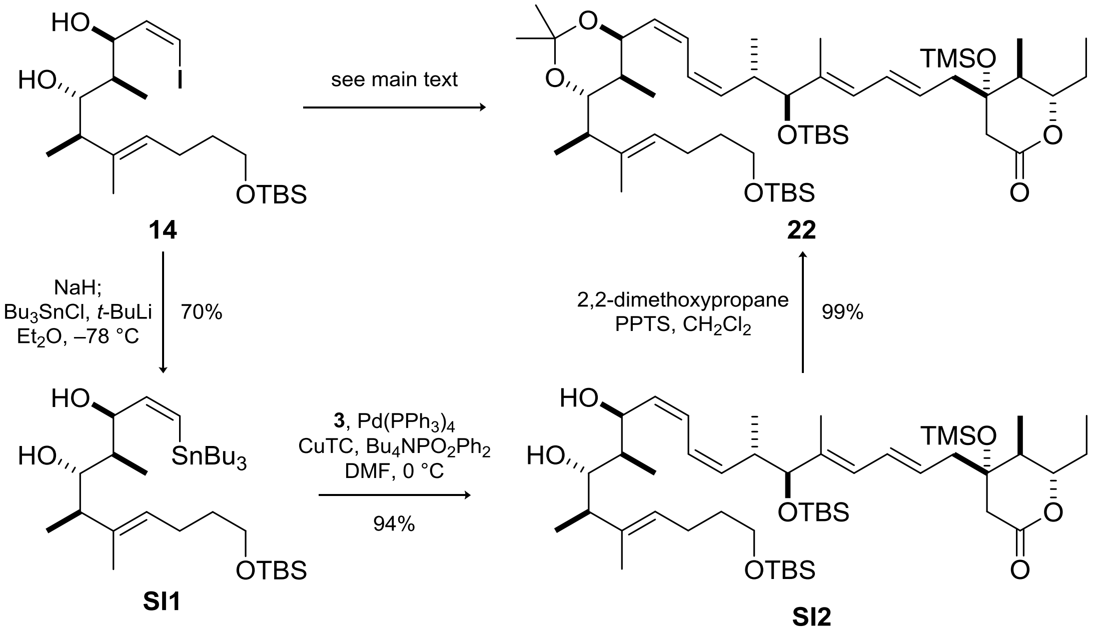

Scheme 1. Alternative route to diene **22**

Vinyl stannane **SI1**

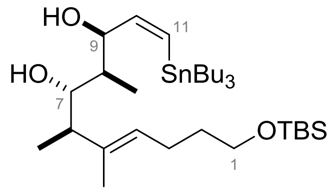

SI1

Sodium hydride (60 wt% suspension in mineral oil, 50.0 mg, 1.25 mmol) was washed with hexane (2 × 1 mL) and suspended in Et_2O (1 mL). The mixture was cooled to 0 °C, a solution of vinyl iodide **14** (60.0 mg, 124 µmol) in Et_2O (2 mL + 4 mL wash) was added *via* cannula and the mixture stirred at rt for 30 min before being cooled to −78 °C. Tributyltin chloride (269 µL, 992 µmol) was added followed by dropwise addition of *t*-butyllithium solution (1.7 M in pentane, 729 µL, 1.24 mmol). A further portion of *t*-butyllithium (200 µL, 340 µmol) was added after 1 h and the reaction stirred for a further 45 min before being quenched with NH_4Cl solution (5 mL) and immediately warmed to rt. The layers were separated and the aqueous phase extracted with CH_2Cl_2 (4 × 5 mL), the combined organic extracts were dried (Na_2SO_4) and concentrated *in vacuo*. Purification by flash column chromatography (Et_3N washed silica gel, (EtOAc / PE 40–60 0:1 →1:10) gave vinyl stannane **SI1** as a colourless oil (56.1 mg, 86.9 µmol, 70%).

R_f 0.85 (MeOH / CH_2Cl_2 1:20); **^{1}H NMR** (400 MHz, $CDCl_3$) δ_H 6.56* (1H, dd, *J* = 13.1, 4.7 Hz, H10), 6.05* (1H, dd, *J* = 13.1, 1.7 Hz, H11), 5.24 (1H, t, *J* = 7.0 Hz, H4), 4.35 (1H, br s, H9), 3.60 (3H, t, *J* = 6.4 Hz, H1), 3.58 (1H, m obs, H7), 3.15 (1H, br s, OH), 2.33 (1H, qd, *J* = 6.8, 4.8 Hz, H6), 2.08 (2H, m, H3), 1.85 (1H, dqd, *J* = 7.2, 7.2, 2.4 Hz, H8), 1.62 (3H, s, Me5), 1.61–1.42 (8H, m, H2, Sn$\underline{Bu_3}$), 1.30 (6H, m, Sn$\underline{Bu_3}$), 1.02 (3H, d, *J* = 6.9 Hz, Me6), 0.92–0.84 (27H, m, Sn$\underline{Bu_3}$, Me8, Si$\underline{t\text{-Bu}}$$Me_2$), 0.05 (6H, s, Si*t*-Bu$\underline{Me_2}$); **^{13}C NMR** (100 MHz, $CDCl_3$) δ_C 147.0, 137.4, 130.5, 126.1, 75.5, 74.6, 62.6, 44.2, 39.2, 32.8, 29.3, 26.0, 24.2, 18.3, 15.6, 13.8, 12.1, 12.0, 11.4, −5.3; **$[\alpha]_D^{20}$** −13.6 (*c* 0.88, $CHCl_3$); **IR** (thin film / cm^{-1}) 3464, 2956, 2927, 2857, 1463, 1255, 1102, 970, 1102, 834, 776, 665; **HRMS** (ESI) calc. for $C_{32}H_{67}O_3Si^{116}Sn$ $[M+H]^+$ 643.3871, found 643.3871.

* These peaks show satellites due to coupling to ^{117}Sn and ^{119}Sn

Diene **SI2**

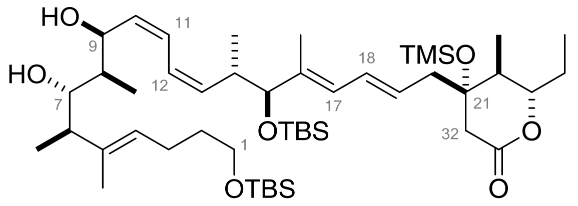

SI2

A degassed solution of vinyl stannane **SI1** (46.3 mg, 71.7 µmol), and vinyl iodide **3** (48.6 mg, 76.6 µmol) in DMF (3.2 mL) was added *via* cannula to a flask containing tetrakistriphenylphosphinepalladium(0) (16.0 mg, 13.8 µmol) and $[Ph_2PO_2][NBu_4]$ (83.0 mg, 180 µmol). The mixture was cooled to 0 °C, copper thiophenecarboxylate (36.0 mg, 188 µmol) was added and the reaction stirred at 0 °C in the dark for 30 min. Water (5 mL) and Et_2O (10 mL) were added, the layers were separated and the aqueous portion extracted with Et_2O (3 × 20 mL). The combined organic extracts were washed with water (2 × 20 mL), dried (Na_2SO_4), concentrated *in vacuo* and purified by flash column chromatography (EtOAc / PE 40–60 1:4) to give diene **SI2** as a yellowish oil (58.0 mg, 67.2 µmol, 94%).

$\mathbf{R_f}$ 0.33 (EtOAc / PE 40–60 1:4); ^{1}**H NMR** (400 MHz, $CDCl_3$) δ_H 6.35 (1H, dd, *J* = 11.5, 11.5 Hz, H11), 6.26 (1H, dd, *J* = 15.0, 10.8 Hz, H18), 6.21 (1H, dd, *J* = 11.3, 11.3 Hz, H12), 5.88 (1H, d, *J* = 10.7 Hz, H17), 5.60 (1H, ddd, *J* = 15.0, 7.5, 7.5 Hz, H19), 5.51 (1H, dd, *J* = 9.8, 9.7 Hz, H10) 5.31 (2H, m, H13, H4), 4.89 (1H, d, *J* = 8.0 Hz, H9), 4.93 (1H, ddd, *J* = 10.3, 7.4, 2.9 Hz, H23), 3.68 (1H, d, *J* = 7.7 Hz, H15), 3.61 (2H, t, *J* = 6.4 Hz, H1), 3.57 (1H, dd, *J* = 6.3, 6.3 Hz, H7), 3.16 (1H, br s, OH), 2.88 (1H, d, *J* = 16.4 Hz, H32a), 2.80 (1H, dq, *J* = 9.3, 7.2 Hz, H14), 2.44 (1H, d, *J* = 16.2 Hz, H32b), 2.42–2.30 (2H, m, H6, H20a), 2.16–2.02 (3H, m, H3, H20b), 1.90–1.80 (3H, m, H8, H22, H24a), 1.69 (3H, s, Me16), 1.60 (3H, s, Me5), 1.62–1.52 (3H, m obs, H2, H24b), 1.07–0.99 (9H, m, H25, Me6, Me22), 0.90 (12H, s + obs, Si*t*-BuMe$_2$, Me8), 0.82 (9H, s, Si*t*-BuMe$_2$), 0.78 (3H, d, *J* = 6.8 Hz, Me14), 0.12 (9H, s, $SiMe_3$), 0.05 (6H, s, C1OSi*t*-BuMe$_2$), −0.03 (3H, s, C15OSi*t*-BuMe_aMe_b), −0.08 (3H, s, C15OSi*t*-BuMe_aMe_b); ^{13}**C NMR** (125 MHz, $CDCl_3$) δ_C 170.1, 138.2, 137.6, 137.5, 131.5, 130.4, 127.3, 126.4, 126.2, 125.3, 123.4, 83.7, 82.8, 77.3, 77.0, 76.8, 75.3, 75.0, 70.1, 62.6, 44.9, 44.0, 43.5, 39.9, 38.6, 36.7, 32.8, 26.9, 26.0, 25.8, 24.2, 18.4, 18.2, 17.6, 15.0, 13.0, 12.1, 11.8, 11.0, 8.9, 2.4, −4.6, −5.0, −5.3; $[\alpha]_D^{20}$ +15.3 (*c* 1.17,

$CHCl_3$); **IR** (thin film / cm^{-1}) 3295, 2957, 2930, 1741, 1462, 1252, 1072, 1006, 971, 837, 774; **HRMS** (ESI) calc. for $C_{48}H_{90}O_7Si_3Na$ $[M+Na]^+$ 885.5887, found 885.5882.

Diene **22**

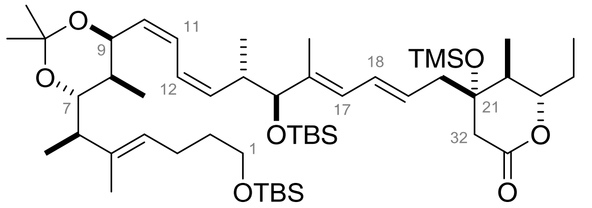

22

via Stille coupling of **2** and **3**

A solution of tetrakistriphenylphosphinepalladium(0) (2.3 mg, 2.0 μmol), copper thiophenecarboxylate (7.8 mg, 41 μmol) and $[Ph_2PO_2][NBu_4]$ (19.0 mg, 41.3 μmol) was prepared in degassed DMF (0.3 mL) and cooled to 0 °C. A degassed solution of vinyl stannane **2** (14.0 mg, 20.4 μmol), and vinyl iodide **3** (13.0 mg, 20.4 μmol) in DMF (0.3 mL) and THF (0.2 mL) was added *via* cannula and the reaction stirred at 0 °C in the dark for 1.5 h and rt for 15 min. Water (2 mL) and Et_2O (2 mL) were added, the layers were separated and the aqueous portion extracted with Et_2O (3 × 5 mL). The combined organic extracts were washed with water (2 × 4 mL) dried ($MgSO_4$), concentrated *in vacuo* and purified by flash column chromatography (EtOAc / PE 40–60 1:20) to give diene **22** as a colourless oil (14.6 mg, 16.1 μmol, 80%).

via protection of **SI2**

Diol **SI2** (32.0 mg, 37.1 μmol) was dissolved in 2,2-dimethoxypropane (1 mL) and CH_2Cl_2 (1 mL) and pyridinium *para*-toluenesulfonate (approx. 5 mg) was added. The reaction was stirred under an argon atmosphere for 1.5 h, $NaHCO_3$ solution (2 mL) was added, the layers were separated and the aqueous phase extracted with CH_2Cl_2 (3 × 5 mL), the combined organic extracts were dried ($MgSO_4$) and concentrated *in vacuo* to yield diene **22** as a colourless oil (33.3 mg, 36.9 μmol, 99%) that was used without further purification.

Alternatively upon completion the reaction mixture could be concentrated *in vacuo* and submitted directly to the subsequent deprotection.

R$_f$ 0.85 (EtOAc / PE 40–60 1:3); **^{1}H NMR** (500 MHz, $CDCl_3$) δ_H 6.35 (1H, dd, *J* = 11.5, 11.5 Hz, H11), 6.26 (1H, dd, *J* = 15.0, 10.9 Hz, H18), 6.19 (1H, dd, *J* = 11.3, 11.3 Hz, H12), 5.89 (1H, d, *J* = 10.7 Hz, H17), 5.60 (1H, ddd, *J* = 15.0, 7.5, 7.5 Hz, H19), 5.40–5.31 (2H, m, H10, H13), 5.23 (1H, t, *J* = 6.9 Hz, H4), 4.83 (1H, dd, *J* = 8.0, 4.6 Hz, H9), 3.93 (1H, ddd, *J* = 10.2, 7.5, 2.8 Hz, H23), 3.68 (1H, d, *J* = 7.8 Hz, H15), 3.59 (2H, t, *J* = 6.4 Hz, H1), 3.25 (1H, dd, *J* = 6.8, 6.8 Hz, H7), 2.88 (1H, d, *J* = 16.4 Hz, H32a), 2.80 (1H, dq, *J* = 9.3, 7.1 Hz, H14), 2.44 (1H, d, *J* = 16.4 Hz, H32b), 2.40 (1H, dd, *J* = 14.2, 6.5 Hz, H20a), 2.18 (1H, dq, *J* = 6.4, 6.4 Hz, H6), 2.10 (1H, dd, *J* = 14.1, 7.9 Hz, H20b), 2.07–1.97 (2H, m, H3), 1.90–1.80 (2H, m, H22, H24a), 1.79–1.73 (1H, m, H8), 1.68 (3H, s, Me16), 1.62–1.50 (3H, m obs, H2, H24b), 1.57 (3H, s, Me5), 1.36 (3H, s, acetonide), 1.34 (3H, s, acetonide), 1.04 (3H, d, *J* = 6.9 Hz, Me6), 1.02 (3H, t, *J* = 7.1 Hz, H25), 0.89 (9H, s, Si*t*-BuMe$_2$), 0.81 (9H, s, Si*t*-BuMe$_2$), 0.80 (3H, d, *J* = 6.8 Hz, Me8), 0.79 (3H, d, *J* = 6.9 Hz, Me14), 0.12 (9H, s, SiMe$_3$), 0.04 (6H, s, C1OSi*t*-BuMe$_2$), −0.03 (3H, s, C15OSi*t*-BuMe$_a$Me$_b$), −0.08 (3H, s, C15OSi*t*-BuMe$_a$Me$_b$); **^{13}C NMR** (125 MHz, $CDCl_3$) δ_C 170.1, 138.3, 137.5, 137.2, 130.4, 128.5, 127.3, 126.4, 125.7, 125.3, 123.4, 100.2, 83.7, 82.8, 75.0, 66.4, 62.7, 47.2, 44.1, 43.5, 39.2, 38.6, 36.8, 32.8, 29.7, 26.9, 26.0, 25.8, 25.6, 24.0, 23.9, 18.3, 18.2, 17.6, 14.9, 13.8, 12.9, 11.8, 11.0, 8.9, 2.4, −4.6, −5.0, −5.3; **$[\alpha]_D^{20}$** +26.3 (*c* 1.37, $CHCl_3$); **IR** (thin film / cm^{-1}) 2929, 1745, 1462, 1278, 1252, 1104; **HRMS** (ESI) calc. for $C_{51}H_{98}O_7Si_3N$ $[M+NH_4]^+$ 920.6646, found 920.6646.

Alcohol **22b**

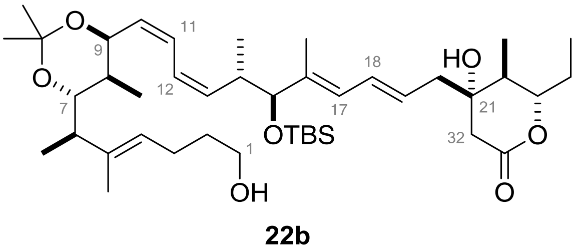

22b

A stock solution of HF·Py and pyridine was prepared by adding HF·Py (approx. 70% HF, 100 μL) to a solution of pyridine (300 μL) in THF (1 mL) at 0 °C and the mixture stirred at rt for 30 min. TBS ether **22** (33.3 mg, 36.9 μmol) was dissolved in THF (2 mL) cooled to 0 °C and an aliquot of the stock solution (800 μL) was added. The reaction was stirred at rt for 3.5 h before cooling to 0 °C and quenched by careful addition of $NaHCO_3$ solution. The layers were separated and the aqueous portion

extracted with EtOAc (3 × 3 mL). The combined organic extracts were dried ($MgSO_4$), concentrated *in vacuo* and the product used without further purification (26.2 mg, 36.5 μmol, 99%).

R_f 0.40 (EtOAc / PE 40–60 1:1); **^{1}H NMR** (500 MHz, $CDCl_3$) δ_H 6.37 (1H, dd, *J* = 15.0, 10.9 Hz, H18), 6.32 (1H, dd, *J* = 11.8, 11.8 Hz, H11), 6.18 (1H, dd, *J* = 11.3, 11.3 Hz, H12), 5.92 (1H, d, *J* = 10.7 Hz, H17), 5.62 (1H, ddd, *J* = 15.1, 7.6, 7.6 Hz, H19), 5.41–5.33 (2H, m, H10, H13), 5.26 (1H, t, *J* = 6.9 Hz, H4), 4.83 (1H, dd, *J* = 8.0, 4.8 Hz, H9), 3.95 (1H, ddd, *J* = 10.1, 7.3, 3.0 Hz, H23), 3.71 (1H, d, *J* = 7.0 Hz, H15), 3.63 (2H, t, *J* = 6.5 Hz, H1), 3.27 (1H, dd, *J* = 6.8, 6.8 Hz, H7), 2.80 (1H, d, *J* = 16.7 Hz, H32a), 2.80 (1H, m obs, H14), 2.41 (1H, d, *J* = 16.8 Hz, H32b), 2.38 (1H, dd, *J* = 13.3, 8.2 Hz, H20a), 2.23 (1H, dd, *J* = 14.2, 7.1 Hz, H20b), 2.20 (1H, dq, *J* = 6.8, 6.8 Hz, H6), 2.12–2.05 (2H, m, H3), 1.95 (1H, dq, *J* = 10.1, 6.9 Hz, H22), 1.87 (1H, dqd, *J* = 14.9, 7.3, 3.1 Hz, H24a), 1.81–1.74 (1H, m, H8), 1.70 (3H, s, Me16), 1.67–1.59 (3H, m obs, H2, H24b), 1.60 (3H, s, Me5), 1.36 (3H, s, acetonide), 1.34 (3H, s, acetonide), 1.08–1.01 (9H, m, H25, Me6, Me22), 0.84 (3H, obs, Me14), 0.83 (9H, s, Si<u>*t*-Bu</u>Me_2), 0.80 (3H, d, *J* = 7.0 Hz, Me8), −0.01 (3H, s, C15OSi*t*-Bu<u>Me_a</u>Me_b), −0.08 (3H, s, C15OSi*t*-BuMe_a<u>Me_b</u>); **^{13}C NMR** (125 MHz, $CDCl_3$) δ_C 170.2, 139.9, 137.6, 137.0, 132.5, 128.7, 125.5, 125.4, 125.1, 124.8, 123.4, 100.3, 83.6, 82.3, 76.9, 71.4, 66.4, 62.8, 47.0, 42.9, 42.5, 39.0, 38.0, 36.9, 32.6, 26.8, 25.8, 25.6, 24.2, 24.0, 18.2, 17.8, 14.8, 14.0, 12.9, 12.3, 11.2, 8.9, −4.6, −5.0; **$[\alpha]_D^{20}$** +32.2 (*c* 0.9, $CHCl_3$); **IR** (thin film / cm^{-1}) 3390, 2929, 2856, 1722, 1458, 1378, 1248, 1226, 1173, 1066, 1005, 890, 836, 775; **HRMS** (ESI) calc. for $C_{42}H_{72}O_7SiNa$ $[M+Na]^+$ 739.4940, found 739.4933.

Acid **22d**

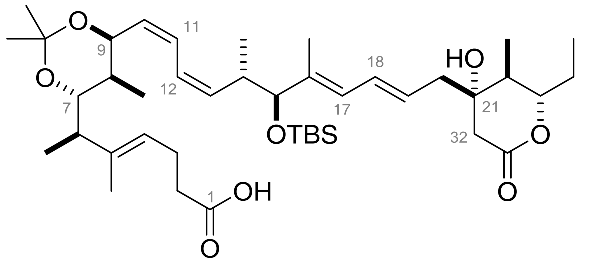

22d

Alcohol **22b** (35.4 mg, 49.4 μmol) was dissolved in CH_2Cl_2 (3.5 mL) and pH 7 buffer solution (0.7 mL). Bisacetoxyiodobenzene (31.8 mg, 98.8 μmol) and 2,2,6,6-tetramethylpiperidine 1-oxyl (0.7 mg, 4.3 μmol) were added and the reaction stirred at

rt for 1.5 h. Sodium thiosulfate solution (3 mL) was added, the layers were separated and the aqueous portion extracted with EtOAc (5 × 3 mL). The combined organic extracts were dried ($MgSO_4$), concentrated *in vacuo* and used directly in the next step. The crude aldehyde was dissolved in a mixture of water (1 mL), *t*-BuOH (1 mL) and THF (1 mL), 2-methyl-2-butene (100 μL), sodium chlorite (13.5 mg, 147 μmol) and sodium dihydrogenphosphate (46.9 mg, 294 μmol) were added and the reaction stirred at rt for 1 h. EtOAc (5 mL) and water (2 mL) were added, the layers were separated and the aqueous portion extracted with EtOAc (5 × 3 mL). The combined organic extracts were dried ($MgSO_4$), concentrated *in vacuo* and purified by preparative thin layer chromatography (EtOAc / PE 40–60 / AcOH 50:50:1) to give acid **22d** as a colourless oil (28.6 mg, 39.2 μmol, 80%).

R_f 0.55 (EtOAc / PE 40–60 / AcOH 50:50:1); **^{1}H NMR** (500 MHz, $CDCl_3$) δ_H 6.38 (1H, dd, *J* = 15.2, 11.2 Hz, H18), 6.33 (1H, dd, *J* = 11.4, 11.4 Hz, H11), 6.18 (1H, dd, *J* = 11.3, 11.3 Hz, H12), 5.92 (1H, d, *J* = 11.0 Hz, H17), 5.62 (1H, ddd, *J* = 14.9, 7.5, 7.5 Hz, H19), 5.41–5.34 (2H, m, H10, H13), 5.23 (1H, t, *J* = 6.2 Hz, H4) 4.83 (1H, dd, *J* = 7.9, 5.0 Hz, H9), 3.95 (1H, ddd, *J* = 10.1, 7.3, 2.9 Hz, H23), 3.73 (1H, d, *J* = 6.9 Hz, H15), 3.26 (1H, dd, *J* = 6.7, 6.7 Hz, H7), 2.80 (1H, d, *J* = 16.6 Hz, H32a), 2.80 (1H, m obs, H14), 2.42 (1H, d, *J* = 16.5 Hz, H32b), 2.39–2.29 (5H, m, H2, H3, H20a), 2.24 (1H, dd, *J* = 14.1, 7.2 Hz, H20b), 2.19 (1H, dq, *J* = 6.5, 6.5 Hz, H6), 1.95 (1H, dq, *J* = 10.1, 6.8 Hz, H22), 1.87 (1H, dqd, *J* = 14.6, 7.4, 3.1 Hz, H24a), 1.78–1.72 (1H, m, H8), 1.70 (3H, s, Me16), 1.68–1.62 (1H, m obs, H24b), 1.61 (3H, s, Me5), 1.36 (3H, s, acetonide), 1.33 (3H, s, acetonide), 1.07–1.01 (9H, m, H25, Me6, Me22), 0.84 (3H, obs, Me14), 0.83 (9H, s, Si<u>*t*-Bu</u>Me_2), 0.79 (3H, d, *J* = 7.0 Hz, Me8), −0.01 (3H, s, C15OSi*t*-Bu<u>Me_a</u>Me_b), −0.07 (3H, s, C15OSi*t*-BuMe_a<u>Me_b</u>); **^{13}C NMR** (125 MHz, $CDCl_3$) δ_C 177.1, 170.5, 139.9, 138.8, 136.9, 132.5, 128.3, 125.3, 125.1, 124.7, 123.7, 123.4, 100.3, 83.6, 76.8, 82.1, 71.5, 66.4, 47.1, 42.8, 42.4, 39.0, 38.0, 36.9, 33.8, 26.8, 25.8, 25.6, 23.9, 23.2, 18.2, 17.8, 14.8, 13.8, 12.9, 12.4, 11.2, 8.9, −4.6, −5.0; **$[\alpha]_D^{20}$** +20.0 (*c* 0.60, $CHCl_3$); **IR** (thin film / cm^{-1}) 3435, 2927, 1714 br, 1459, 1378, 1248, 1225, 1064, 1006, 836, 775; **HRMS** (ESI) calc. for $C_{42}H_{74}O_8SiN$ $[M+NH_4]^+$ 748.5178, found 748.5177.

Acid **23**

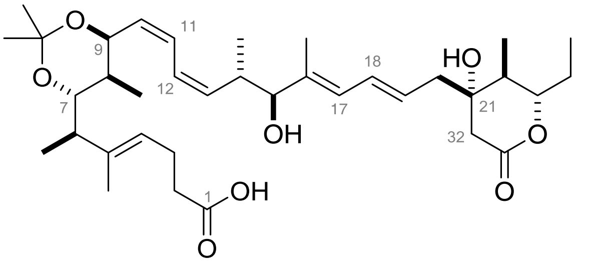

23

To a solution of TBS ether **22d** (10.3 mg, 14.1 μmol) in THF (1 mL) was added a solution of tetrabutylammonim fluoride (1 M in THF, 141 μL, 141 μmol) and the reaction was heated to 50 °C for 16 h. The reaction was cooled to rt and quenched with NH_4Cl solution (1 mL), the layers were separated and the aqueous portion extracted with EtOAc (5 × 3 mL). The combined organic extracts were dried ($MgSO_4$), concentrated *in vacuo* and purified by flash column chromatography (EtOAc / PE 40–60 / AcOH 50:50:1) to give seco acid **23** as a colourless oil (5.7 mg, 9.0 μmol, 64%).

R_f 0.55 (EtOAc / PE 40–60 / AcOH 60:40:1); **^{1}H NMR** (500 MHz, $CDCl_3$) δ_H 6.40 (1H, dd, *J* = 15.1, 10.8 Hz, H18), 6.34–6.25 (2H, m, H11, H12), 6.03 (1H, d, *J* = 10.8 Hz, H17), 5.70 (1H, ddd, *J* = 15.1, 7.5, 7.5 Hz, H19), 5.49 (1H, dd, *J* = 9.1, 9.1 Hz, H10), 5.39 (1H, dd, *J* = 9.6, 9.6 Hz, H13), 5.34 (1H, t, *J* = 6.2 Hz, H4), 4.88 (1H, dd, *J* = 7.0, 4.8 Hz, H9), 3.95 (1H, ddd, *J* = 10.1, 7.3, 3.0 Hz, H23), 3.75 (1H, d, *J* = 9.3 Hz, H15), 3.18 (1H, dd, *J* = 8.7, 6.8 Hz, H7), 2.85 (1H, m, H14), 2.08 (1H, d, *J* = 16.7 Hz, H32a), 2.41 (1H, d, *J* = 16.7 Hz, H32b), 2.41–2.28 (4H, m, H3, H6, H20a), 2.27–2.19 (3H, m, H2, H20b), 1.95 (1H, dq, *J* = 10.1, 6.9 Hz, H22), 1.87 (1H, dqd, *J* = 14.6, 7.3, 3.1 Hz, H24a), 1.84–1.79 (1H, m, H8), 1.77 (3H, s, Me16), 1.69–1.59 (1H, m, H24b), 1.55 (3H, s, Me5), 1.37 (3H, s, acetonide), 1.36, (3H, s, acetonide), 1.07–1.01 (9H, m, H25, Me6, Me22), 0.85 (3H, d, *J* = 6.7 Hz, Me14), 0.74 (3H, d, *J* = 7.8 Hz, Me8); **^{13}C NMR** (125 MHz, $CDCl_3$) δ_C 175.8, 170.3, 137.3, 136.7, 135.2, 131.9, 131.6, 127.7, 126.6, 125.8, 125.5, 123.1, 100.3, 83.7, 82.4, 71.6, 66.8, 65.9, 48.6, 42.8, 42.6, 39.2, 38.0, 36.0, 34.0, 26.8, 25.9, 24.1, 23.1, 17.3, 15.7, 13.1, 13.0, 11.7, 11.3, 9.0; **$[\alpha]_D^{20}$** −83.3 (*c* 0.12, $CHCl_3$); **IR** (thin film / cm^{-1}) 3441, 2933, 1725, 1457, 1378, 1224, 1005; **HRMS** (ESI) calc. for $C_{36}H_{60}O_8N$ $[M+NH_4]^+$ 634.4313, found 634.4311.

It was found to be possible to reverse the order of acetonide deprotection and macrolactonisation albeit in lower yield for the deprotection step. These compounds are described below.

Acid **SI3**

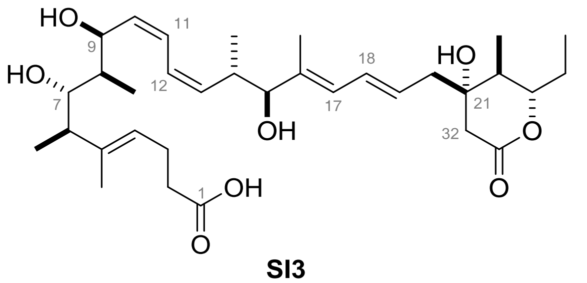

SI3

To a solution of TBS ether **22d** (12.7 mg, 17.3 μmol) in THF (1.2 mL) was added a solution of tetrabutylammonim fluoride (1 M in THF, 174 μL, 174 μmol) and the reaction was heated to 50 °C for 16 h. The reaction was cooled to rt and quenched with NH_4Cl solution (1 mL), the layers were separated and the aqueous portion extracted with EtOAc (5 × 3 mL). The combined organic extracts were dried ($MgSO_4$), concentrated *in vacuo* and redissolved in MeOH (1 mL) and cooled to 5 °C. Dowex® 50WX8 (55 mg) was added and the reaction stirred at 5 °C for 2.5 h before being filtered and concentrated *in vacuo*. Purification by preparative thin layer chromatography (EtOAc / PE 40–60 / AcOH 80:20:1) gave acid **SI3** as a colourless oil (4.2 mg, 7.1 μmol, 41%).

$\mathbf{R_f}$ 0.37 (EtOAc / PE 40–60 / AcOH 80:20:1); **^{1}H NMR** (500 MHz, $CDCl_3$) δ_H 6.48–6.33 (3H, m, H11, H12, H18), 6.03 (1H, d, J = 10.7 Hz, H17), 5.70 (1H, ddd, J = 15.1, 7.6, 7.6 Hz, H19), 5.62 (1H, dd, J = 9.5, 9.5 Hz, H10), 5.42 (1H, dd, J = 10.0, 10.0 Hz, H13), 5.26 (1H, t, J = 5.9 Hz, H4), 4.96 (1H, d, J = 8.6 Hz, H9), 3.95 (1H, ddd, J = 10.2, 7.2, 3.0 Hz, H23), 3.76 (1H, d, J = 8.9 Hz, H15), 3.56 (1H, dd, J = 6.7, 5.6 Hz, H7), 2.93–2.84 (1H, m, H14), 2.80 (1H, d, J = 16.7 Hz, H32a), 2.45–2.26 (7H, m, H2, H3, H6, H20a, H32b), 2.22 (1H, dd, J = 14.0, 7.4 Hz, H20b), 1.94 (1H, dq, J = 10.1, 6.9 Hz, H22), 1.87 (1H, dqd, J = 14.6, 7.3, 3.1 Hz, H24a), 1.82–1.78 (1H, m obs, H8), 1.77 (3H, s, Me16), 1.64 (1H, ddq, J = 7.2, 7.2, 7.2 Hz, H24b), 1.58 (3H, s, Me5), 1.08 (3H, d, J = 6.8 Hz, Me6), 1.05–1.01 (6H, m, H25, Me22), 1.00 (3H, d, J = 7.2 Hz, Me8), 0.86 (3H, d, J = 6.7 Hz, Me14); **^{13}C NMR** (125 MHz, $CDCl_3$) δ_C 175.8, 170.4, 138.7, 137.5, 135.8, 132.4, 132.0, 127.4, 126.4, 125.3, 124.8, 124.5, 83.7, 82.3, 76.8

obs, 71.6, 68.5, 45.6, 42.8, 42.6, 39.0, 38.0, 36.0, 33.6, 29.7, 26.8, 23.2, 17.4, 14.2, 14.1, 12.0, 11.9, 11.3, 9.0; $[\alpha]_D^{20}$ −10.0 (*c* 0.20, $CHCl_3$); **IR** (thin film / cm^{-1}) 3425, 2935, 1722, 1712, 1455, 1378, 1260, 1070, 1006, 969, 795; **HRMS** (ESI) calc. for $C_{33}H_{56}O_8N$ $[M+NH_4]^+$ 594.4000, found 594.3995.

Acetonide protected macrocycle **23f**

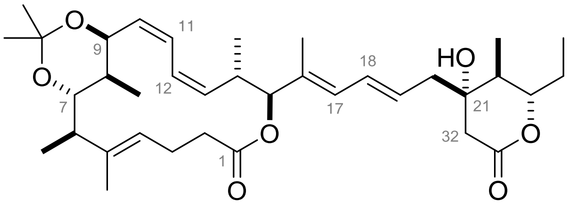

23f

To a solution of acid **23** (5.0 mg, 8.1 μmol) in THF (1.5 mL) was a added triethylamine (22.6 μL, 162 μmol) and 2,4,6-trichlorobenzoyl chloride (12.7 μL, 81.1 μmol) and the mixture stirred for 1 h at rt. Toluene (4.5 mL) was added and the resulting solution added *via* syringe pump to a solution of DMAP (19.8 mg, 162 μmol) in toluene (6.0 mL) over 3 h. The reaction was stirred for a further hour before being concentrated *in vacuo* and purified by flash column chromatography (EtOAc / PE 40–60 1:2) to give macrocycle **23f** as a colourless oil (3.9 mg, 6.49 μmol, 80%).

$\mathbf{R_f}$ 0.56 (EtOAc / PE 40–60 1:1); ^{1}**H NMR** (500 MHz, $CDCl_3$) δ_H 6.35 (1H, dd, *J* = 15.0, 10.9 Hz, H18), 6.18 (1H, dd, *J* = 10.5, 10.5 Hz, H12), 6.08 (1H, dd, *J* = 10.6, 10.6 Hz, H11), 6.06 (1H, d, *J* = 10.9 Hz, H17), 5.72 (1H, ddd, *J* = 15.0, 7.5, 7.5 Hz, H19), 5.58 (1H, dd, *J* = 10.8, 7.0 Hz, H10), 5.36–5.28 (2H, m, H4, H13), 5.14 (1H, d, *J* = 10.3 Hz, H15), 4.91 (1H, br s, H9), 3.95 (1H, ddd, *J* = 10.0, 7.4, 2.8 Hz, H23), 3.17 (1H, dd, *J* = 10.0, 4.9 Hz, H7), 2.88–2.80 (1H, m obs, H14), 2.80 (1H, d, *J* = 16.8 Hz, H32a), 2.47–2.15 (8H, m, H2, H3, H6, H20a, H20b, H32b), 1.97–1.83 (2H, m, H22, H24a), 1.82–1.76 (1H, m obs, H8), 1.75 (3H, s, Me16), 1.63 (1H, ddq, *J* = 14.4, 7.2, 7.2 Hz, H24b), 1.49 (3H, s, Me5), 1.08 (3H, d, *J* = 6.7 Hz, Me6), 1.06–1.01 (6H, m, H25, Me22), 0.85 (3H, d, *J* = 6.8 Hz, Me14), 0.76 (3H, d, *J* = 6.9 Hz, Me8); ^{13}**C NMR** (125 MHz, $CDCl_3$) δ_C 172.4, 170.1, 135.1, 134.8, 134.3, 132.6, 131.7, 129.2, 127.3, 127.1, 124.2, 123.1, 100.4, 83.7, 83.0, 77.0 obs, 71.7, 66.5, 49.1, 42.8, 42.6, 38.0, 37.9, 35.4, 34.5, 26.9, 26.7, 24.9, 22.0, 16.9, 15.4, 14.1, 13.1, 12.0, 11.3, 9.0; $[\alpha]_D^{20}$ −50.0 (*c* 0.17, $CHCl_3$); **IR** (thin film / cm^{-1}); 2956, 2925, 1730, 1661, 1456,

1376, 1260, 1096, 1091, 800; **HRMS** (ESI) calc. for $C_{36}H_{55}O_7$ $[M+H]^+$ 599.3973, found 599.3948.

Macrocycle **24**

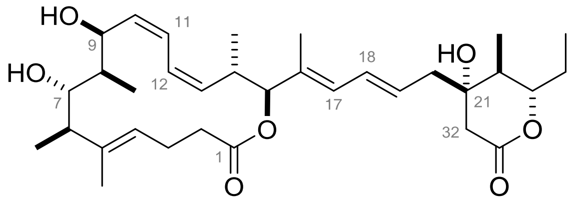

24

via deprotection of acetonide **23f**

Acetonide **23f** (3.9 mg, 6.49 μmol) was dissolved in MeOH (1 mL) and Dowex® 50WX8 (4 mg) was added. The mixture was stirred at rt for 30 min before being filtered through glass wool and concentrated *in vacuo* to give macrocycle **24** as an amorphous solid (3.5 mg, 5.9 μmol, 91%) that was used without further purification.

via macrolactonisation of acid **SI3**

To a solution of acid **SI3** (2.5 mg, 4.20 μmol) in THF (0.5 mL) was a added triethylamine (5.9 μL, 42 μmol) and 2,4,6-trichlorobenzoyl chloride (4.5 μL, 28.7 μmol) and the mixture stirred for 1 h at rt. Toluene (2 mL) was added and the resulting solution added *via* syringe pump to a solution of DMAP (11.0 mg, 42.1 μmol) in toluene (2 mL) over 1.5 h. The reaction was stirred for a further 16 h before being concentrated *in vacuo* and purified by flash column chromatography (EtOAc / PE 40–60 1:1) to give macrocycle **24** as an amorphous solid (2.0 mg, 3.48 μmol, 83%).

R$_f$ 0.42 (EtOAc / PE 40–60 1:1); **^{1}H NMR** (500 MHz, CDCl$_3$) δ_H 6.47 (1H, dd, J = 11.3, 11.3 Hz, H12), 6.36 (1H, dd, J = 15.2, 10.9 Hz, H18), 6.31 (1H, dd, J = 11.2, 11.2 Hz, H11), 6.08 (1H, d, J = 10.8 Hz, H17), 5.73 (1H, ddd, J = 15.1, 7.6, 7.6 Hz, H19), 5.61 (1H, dd, J = 10.3, 10.3 Hz, H10), 5.32 (1H, dd, J = 10.6, 10.6 Hz, H13), 5.17 (1H, dd, J = 10.7, 3.1 Hz, H4), 5.10 (2H, app d, J = 10.3 Hz, H9, H15), 3.95 (1H, ddd, J = 10.3, 7.4, 3.1 Hz, H23), 3.47 (1H, dd, J = 10.3, 4.6 Hz, H7), 2.97 (1H, ddq, J = 10.1, 10.1, 6.6 Hz, H14), 2.80 (1H, d, J = 16.7 Hz, H32a), 2.50 (1H, dq, J = 10.6, 6.7 Hz, H6) 2.43–2.34 (3H, m, H3a, H20a, H32b), 2.28 (1H, br t, J = 12.9 Hz, H2a), 2.25–2.16 (2H, m, H2b, H20b), 1.97–1.91 (2H, m, H3b, H22), 1.86 (1H, ddq, J = 14.6,

7.3, 3.1 Hz, H24a), 1.79 (3H, s, Me16), 1.68–1.60 (1H, m, H8), H24b), 1.58 (3H, s, Me5), 1.14 (3H, d, J = 6.7 Hz, Me6), 1.13 (3H, d, J = 7.3 Hz, Me8), 1.06–1.02 (6H, m, H25, Me22), 0.88 (3H, d, J = 6.7 Hz, Me14); **^{13}C NMR** (125 MHz, $CDCl_3$) δ_C 172.2, 170.0, 136.6, 136.5, 133.8, 131.7, 131.1, 129.3, 127.4, 125.9, 125.0, 123.9, 83.6, 82.5, 80.1, 71.6, 65.0, 47.9, 42.8, 42.6, 38.0, 37.9, 34.9, 33.5, 26.8, 21.8, 16.5, 16.0, 12.1, 11.7, 11.2 (overlapping), 8.9; **$[\alpha]_D^{20}$** −44.2 (c 0.35, $CHCl_3$); **IR** (thin film / cm^{-1}) 3434, 2963, 2925, 2853, 1726, 1457, 1376, 1260, 1214, 1147, 1079, 1018, 969, 801, 753; **HRMS** (ESI) calc. for $C_{33}H_{50}O_7Na$ $[M+Na]^+$ 581.3467, found 581.3454.

Leiodermatolide (1)

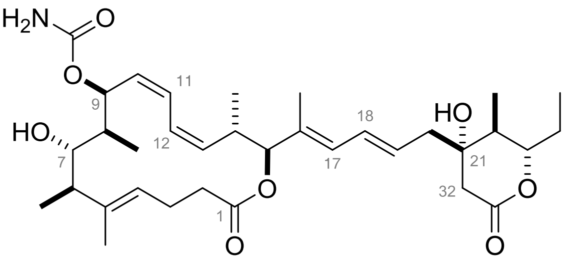

1

Macrocycle **24** (0.49 mg, 0.86 μmol), was dissolved in CH_2Cl_2 (100 μL) and 1-trimethylsilyl imidazole (6.7 μL, 45.4 μmol) was added. The reaction was stirred at rt for 2 h before judged complete by TLC. MeOH (7 μL) was added along with pyridinium *para*-toluenesulfonate (1 crystal) and the reaction stirred for 4 hours. The mixture was applied directly to a short column of silica washed with triethylamine and eluted with EtOAc / PE 40–60 2:3. The product was concentrated *in vacuo*, dissolved in CH_2Cl_2 (300 μL), cooled to −78 °C and trichloroacetylisocyanate (2 μL, 16.8 μmol) was added. The reaction was stirred at −78 °C for 30 min, quenched with MeOH (200 μL) and concentrated *in vacuo*. The residue was taken up in CH_2Cl_2 (1 mL) and loaded onto a short column of alumina. After 1 h the column was flushed with MeOH and CH_2Cl_2 (1:3, 2 mL), the solvents were removed and the residue redissolved in MeOH (250 μL) and CH_2Cl_2 (500 μL). Pyridinium *para*-toluenesulfonate (2 crystals) was added and the reaction stirred for 30 min at rt before being concentrated *in vacuo* and purified by preparative thin layer chromatography (PE 40–60 / EtOAc / MeOH 60:40:5) to yield leiodermatolide (**1**) as an amorphous white solid (280 μg, 0.45 μmol, 53%).

via direct carbamoylation of macrocycle **24**

Macrocycle **24** (3.0 mg, 5.2 μmol) was dissolved in CH_2Cl_2 (1 mL), cooled to −78 °C and trichloroacetylisocyanate (50 μL of a 0.105 M solution in CH_2Cl_2, 5.25 μmol) was added. The reaction was stirred at −78 °C for 40 min, quenched with MeOH (200 μL) and concentrated *in vacuo*. The residue was taken up in CH_2Cl_2 (1 mL) and loaded onto a short column of alumina. After 1 h the column was flushed with MeOH and CH_2Cl_2 (1:3, 2 mL), the solvents were removed to give a 4:1 mixture of regioisomers. These were separated by preparative thin layer chromatography (PE 40–60 / EtOAc / MeOH 60:40:5) to yield leiodermatolide (**1**) as an amorphous white solid (300 μg, 0.49 μmol, 9%) along with regioisomeric carbamate **SI4** (2.1 mg, 3.4 μmol, 66%).

R_f 0.32 (PE 40–60 / EtOAc / MeOH 60:40:5); **^{1}H NMR** (500 MHz, CD_2Cl_2) δ_H 6.54 (1H, dd, *J* = 11.4, 11.4 Hz, H12), 6.39 (2H, m, H11, H18), 6.10 (1H, d, *J* = 10.7 Hz, H17), 5.90 (1H, d, *J* = 10.2 Hz, H9), 5.76 (1H, ddd, *J* = 15.0, 7.5, 7.5 Hz, H19), 5.53 (1H, dd, *J* = 10.5, 10.5 Hz, H10), 5.35 (1H, dd, *J* = 9.9, 9.9 Hz, H13), 5.10 (1H, m, H4), 5.07 (1H, d, *J* = 10.4 Hz, H15), 4.66 (2H, br s, NH_2), 3.91 (1H, ddd, *J* = 10.0, 7.4, 2.9 Hz, H23), 3.27 (1H, d, *J* = 10.4 Hz, H7), 2.98 (1H, ddq, *J* = 10.1, 10.1, 6.7 Hz, H14), 2.73 (1H, d, *J* = 16.8 Hz, H32a), 2.47 (1H, dq, *J* = 10.1, 6.6 Hz, H6) 2.42 (1H, dd, *J* = 14.0, 7.4 Hz, H20a), 2.35 (1H, d, *J* = 16.5 Hz, H32b), 2.31 (1H, ddd, *J* = 16.7, 6.3, 2.5 Hz, H2a), 2.23 (1H, m, H20b), 2.21 (2H, m, H3a, H3b), 2.00 (1H, m, H2b), 1.90 (1H, dq, *J* = 10.1, 7.0 Hz, H22), 1.86 (1H, ddq, *J* = 14.6, 7.4, 3.3 Hz, H24a), 1.80 (3H, d, *J* = 0.9 Hz, Me16), 1.74 (1H, br q, *J* = 7.6 Hz, H8), 1.63 (1H, ddq, *J* = 14.6, 7.3, 7.3 Hz, H24b), 1.43 (3H, s, Me5), 1.13 (3H, d, *J* = 6.7 Hz, Me6), 1.09 (3H, d, *J* = 7.3 Hz, Me8), 1.02 (3H, d, *J* = 6.9 Hz, Me22), 1.02 (3H, t, *J* = 7.3 Hz, H25), 0.87 (3H, d, *J* = 6.7 Hz, Me14); **^{13}C NMR** (125 MHz, CD_2Cl_2) δ_C 172.2, 170.1, 157.3, 137.7, 137.5, 134.1, 131.7, 129.7, 128.6, 128.2, 126.2, 125.7, 124.5, 83.9, 82.6, 78.2, 72.2, 67.8, 48.6, 43.2, 43.0, 39.4, 38.7, 35.1, 33.8, 27.2, 22.3, 16.6, 16.5, 12.5, 12.1, 11.7, 11.3, 9.2; $[\alpha]_D^{20}$ −74.0 (*c* 0.027, MeOH); **HRMS** (ESI) calc. for $C_{34}H_{52}NO_8$ $[M+H]^+$ 602.3693, found 602.3713.

Carbamate **SI4**

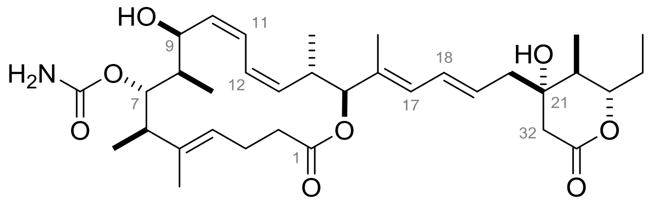

SI4

$\mathbf{R_f}$ 0.29 (PE 40–60 / EtOAc / MeOH 60:40:5); **^{1}H NMR** (500 MHz, $CDCl_3$) δ_H 6.47 (1H, dd, *J* = 11.3, 11.3 Hz, H12), 6.37 (1H, dd, *J* = 14.9, 10.9 Hz, H18), 6.33 (1H, dd, *J* = 11.2, 11.2 Hz, H11), 6.09 (1H, d, *J* = 10.9 Hz, H17), 5.73 (1H, ddd, *J* = 15.2, 7.6, 7.6 Hz, H19), 5.58 (1H, dd, *J* = 10.3, 10.3 Hz, H10), 5.34 (1H, dd, *J* = 10.6, 10.6 Hz, H13), 5.17 (1H, dd, *J* = 10.6, 3.7 Hz, H4), 5.10 (1H, d, *J* = 10.1 Hz, H15), 5.08 (1H, m, H9), 4.85 (1H, d, *J* = 10.3 Hz, H7), 4.66 (2H, br s, NH_2), 3.95 (1H, ddd, *J* = 10.1, 7.3, 3.0 Hz, H23), 2.98 (1H, ddq, *J* = 10.2, 10.2, 6.6 Hz, H14), 2.80 (1H, d, *J* = 16.7 Hz, H32a), 2.62 (1H, m, H6), 2.44–2.34 (3H, m, H3a, H20a, H32b), 2.30 (1H, br t, *J* = 13.1 Hz, H2a), 2.26–2.17 (2H, m, H3b, H20b), 2.03–1.91 (2H, m, H2b, H22), 1.88 (1H, ddq, *J* = 14.6, 7.3, 3.1 Hz, H24a), 1.80 (3H, d, *J* = 0.9 Hz, Me16), 1.76 (1H, br q, *J* = 7.5 Hz, H8), 1.65 (1H, ddq, *J* = 14.6, 7.3, 7.3 Hz, H24b), 1.50 (3H, s, Me5), 1.07–1.02 (12H, m, Me6, Me8, Me22, H25), 0.88 (3H, d, *J* = 6.7 Hz, Me14); **^{13}C NMR** (125 MHz, $CDCl_3$) δ_C 172.2, 170.0, 156.5, 136.6, 135.9, 133.9, 131.7, 131.1, 129.3, 127.3, 126.5, 124.8, 123.9, 83.6, 82.4, 81.7, 71.2, 64.4, 46.1, 42.8, 42.6, 38.5, 37.9, 26.8, 21.9, 16.6, 15.6, 12.0, 11.31, 11.25, 11.24, 8.9; $[\alpha]_D^{20}$ −62.5 (*c* 0.80, MeOH); **IR** (thin film / cm^{-1}) 3378, 2928, 1730, 1375, 1332, 1212, 1149, 1057, 750; **HRMS** (ESI) calc. for $C_{34}H_{55}N_2O_8$ $[M+NH_4]^+$ 619.3953, found 619.3950.

III. Comparison of NMR data for natural and synthetic leiodermatolide

atom	δ_C (125.75 MHz, CD_2Cl_2)		δ_H (500.05 MHz, CD_2Cl_2)					
	natural	synthetic	natural	mult	J (Hz)	synthetic	mult	J (Hz)
1	172.2	172.2						
2a	33.8	33.8	2.31	ddd	16.5, 6.4, 2.4	2.31	ddd	16.7, 6.3, 2.5
2b			2.00	ddd	16.5, 11.0, 3.1	2.00	m	n.d.
3a	22.3	22.3	2.21	m	n.d.	2.21	m	n.d.
3b			2.21	m	n.d.	2.21	m	n.d.
4	125.7	125.7	5.10	dd	8.5, 5.5	5.10	m	n.d.
5	137.3	137.5						
6	48.6	48.6	2.46	dq	10.5, 6.6	2.47	dq	10.1, 6.6
7	78.2	78.2	3.26	br d	9.7	3.27	br d	10.4
8	39.4	39.4	1.74	br q	7.2	1.74	br q	7.6
9	67.7	67.8	5.90	d	10.2	5.90	d	10.2
10	128.6	128.6	5.53	dd	10.3, 10.3	5.53	dd	10.5, 10.5
11	126.2	126.2	6.38	dd	11.5, 11.5	6.38	dd	11.2, 11.2
12	124.5	124.5	6.54	dd	11.4, 11.4	6.54	dd	11.4, 11.4
13	137.7	137.7	5.36	dd	10.4, 10.4	5.35	dd	9.9, 9.9
14	35.1	35.1	2.98	ddq	10.2, 10.2, 6.6	2.98	ddq	10.1, 10.1, 6.7
15	82.6	82.6	5.07	d	10.4	5.07	d	10.4
16	134.0	134.1						
17	129.7	129.7	6.10	d	10.9	6.10	d	10.7
18	131.7	131.7	6.40	dd	14.8, 10.9	6.40	dd	15.1, 10.7
19	128.2	128.2	5.76	ddd	15.1, 7.6, 7.6	5.76	ddd	15.0, 7.5, 7.5
20a	38.7	38.7	2.42	dd	14.0, 7.4	2.42	dd	14.0. 7.4
20b			2.23	m	n.d.	2.23	m	n.d.
21	72.2	72.2						
22	43.2	43.2	1.89	dq	9.9, 6.8	1.90	dd	10.1, 7.0
23	83.9	83.9	3.91	ddd	10.1, 7.4, 2.9	3.91	ddd	10.0, 7.4, 2.9
24a	27.2	27.2	1.84	ddq	14.8, 7.4, 3.2	1.86	ddq	14.6, 7.4, 3.3
24b			1.63	ddq	14.6, 7.3, 7.3	1.63	ddq	14.6, 7.3, 7.3
25	9.2	9.2	1.02	t	7.3	1.02	t	7.3
Me5	11.3	11.3	1.43	s		1.43	s	
Me6	16.5	16.5	1.13	d	6.7	1.13	d	6.7
Me8	12.5	12.5	1.09	d	7.4	1.09	d	7.3
Me14	16.6	16.6	0.87	d	6.7	0.87	d	6.7
Me16	12.1	12.1	1.80	d	0.8	1.80	d	0.9
Me22	11.6	11.7	1.02	d	6.9	1.02	d	6.9
32a	42.9	43.0	2.73	d	16.5	2.73	d	16.8
32b			2.35	d	16.3	2.35	d	16.5
33	170.1	170.1						
34	157.3	157.3						
NH_2			4.65	br s		4.66	br s	

The published data for leiodermatolide was recorded at 600 MHz (^{1}H) and 150 MHz (^{13}C). We are in possession of an authentic sample of leiodermatolide and so were able to do a direct comparison at 500 / 125 MHz. We thank Dr Amy Wright for the kind donation of an authentic sample.

IV. ^{1}H and ^{13}C NMR spectra

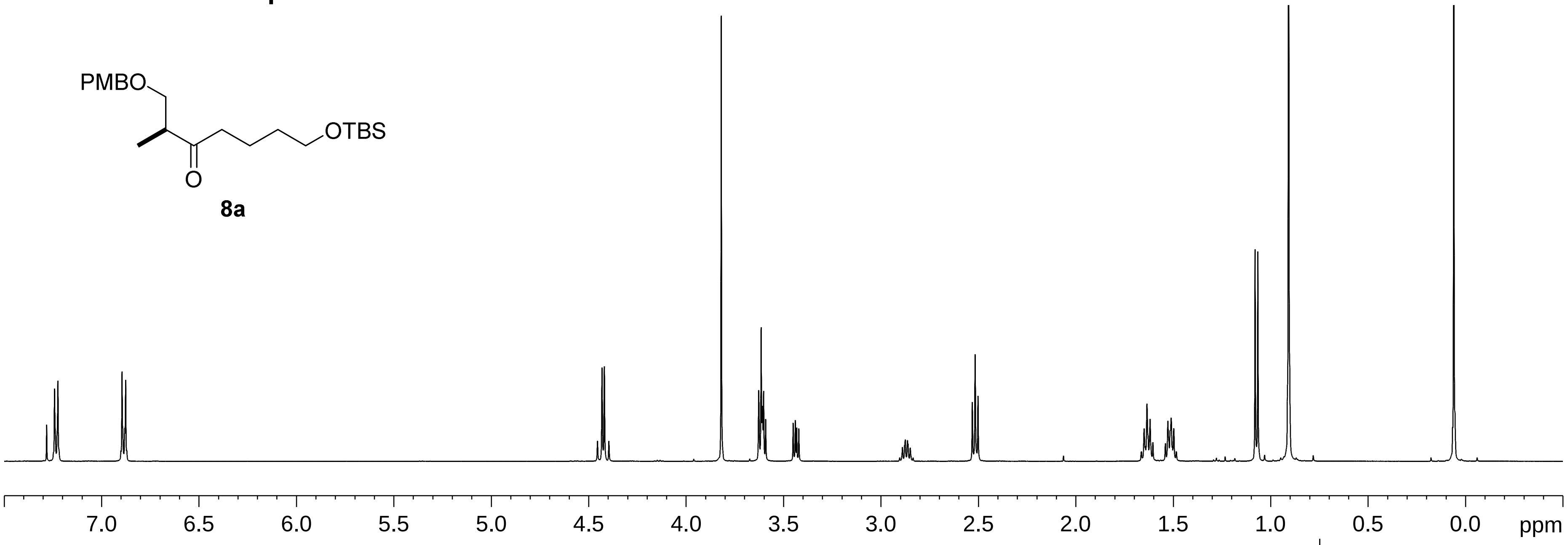

8a

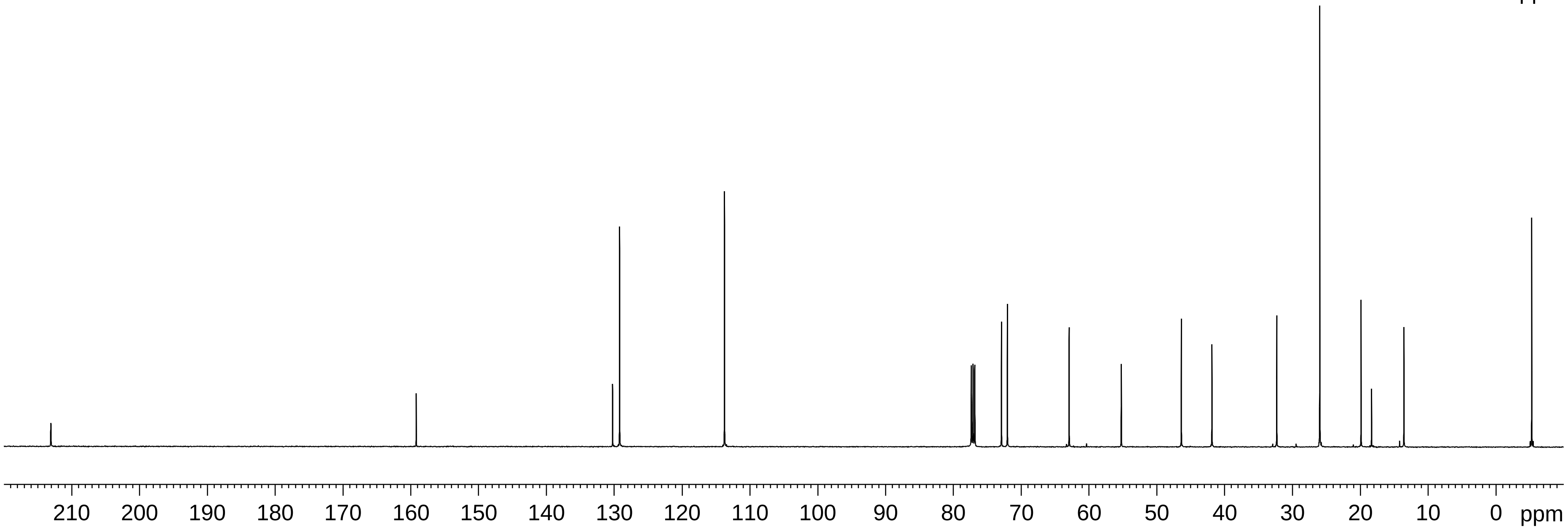

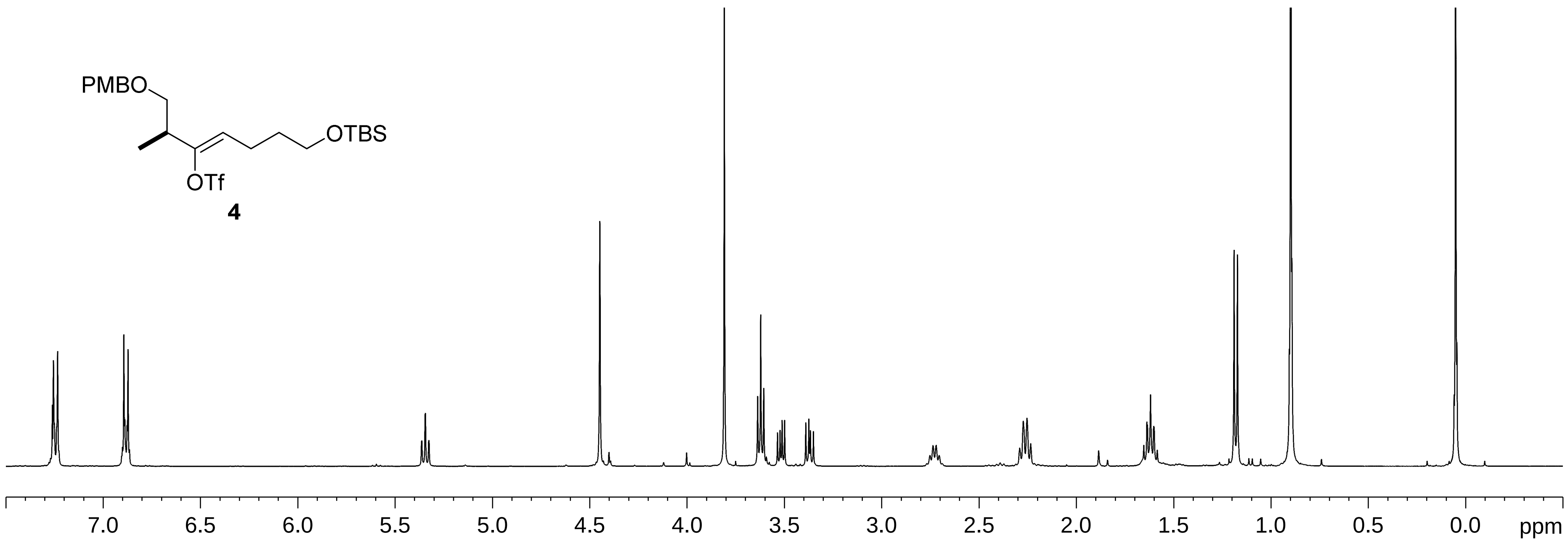
PMBO
OTBS
OTf
4
7.0 6.5 6.0 5.5 5.0 4.5 4.0 3.5 3.0 2.5 2.0 1.5 1.0 0.5 0.0 ppm

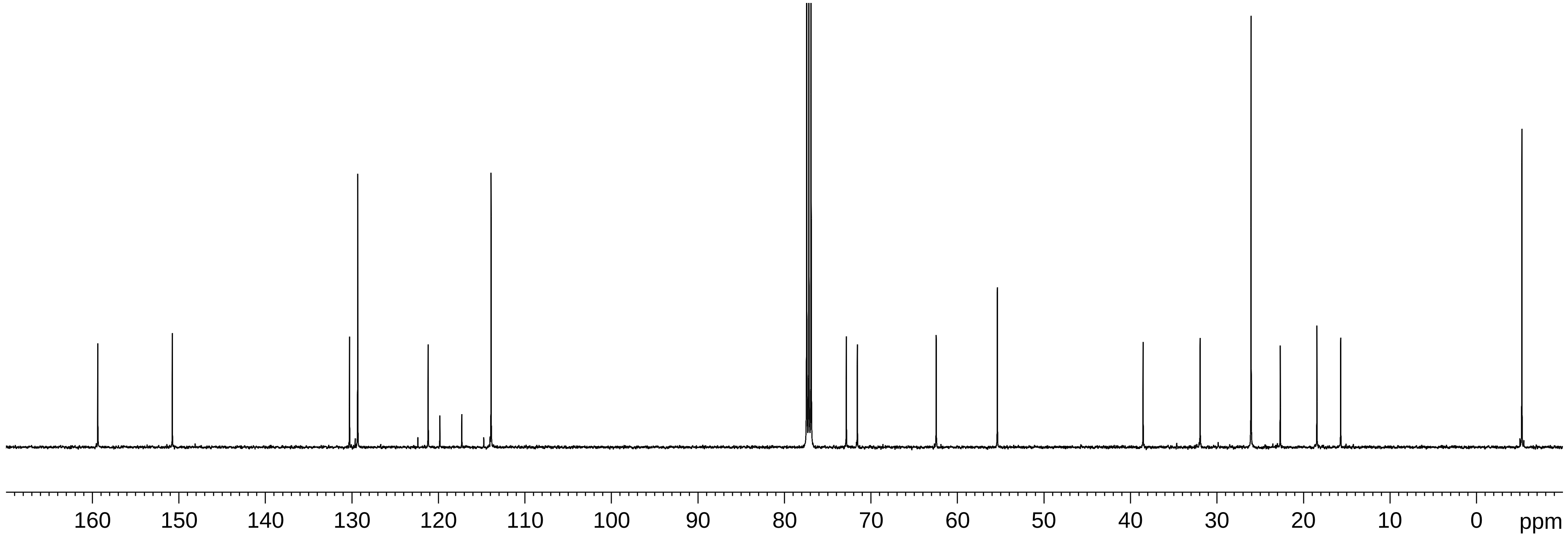
160 150 140 130 120 110 100 90 80 70 60 50 40 30 20 10 0 ppm

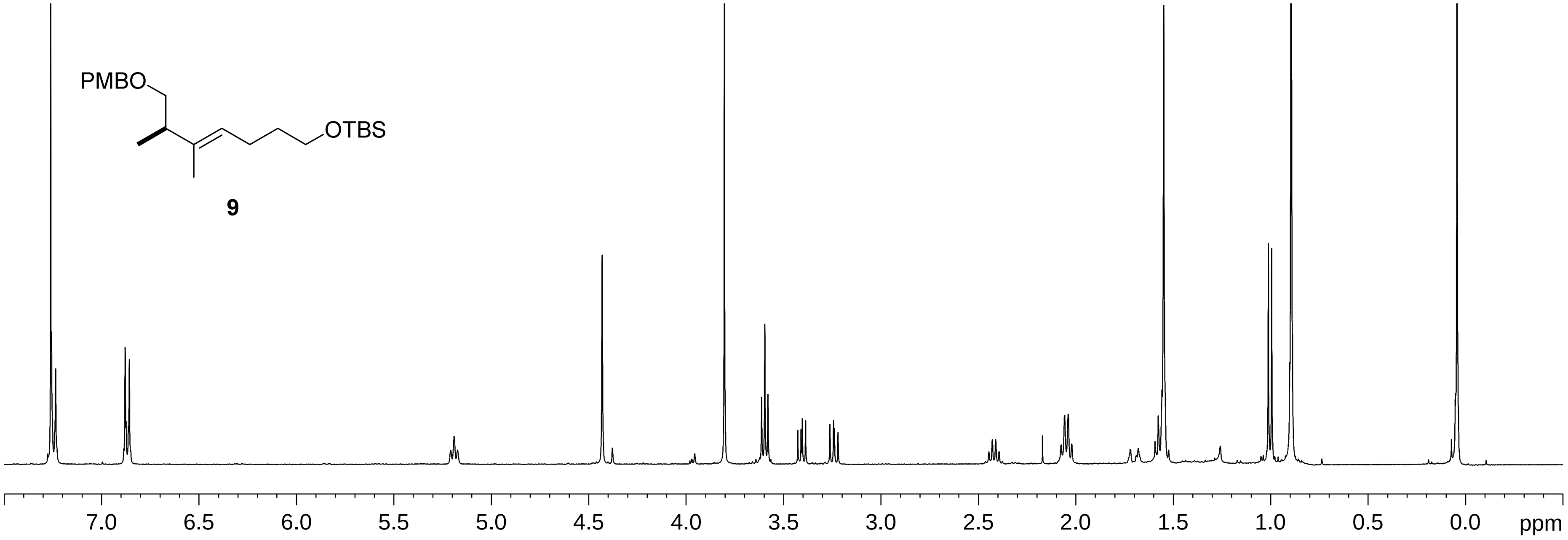
PMBO
OTBS
9
7.0 6.5 6.0 5.5 5.0 4.5 4.0 3.5 3.0 2.5 2.0 1.5 1.0 0.5 0.0 ppm

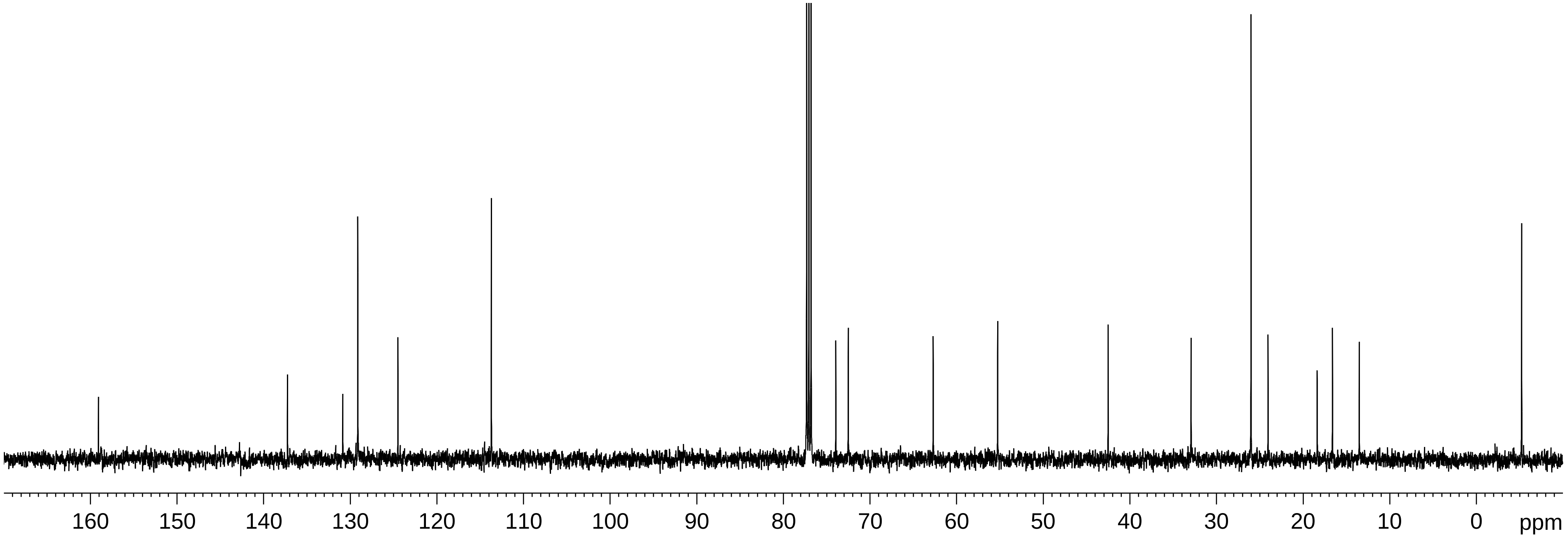
160 150 140 130 120 110 100 90 80 70 60 50 40 30 20 10 0 ppm

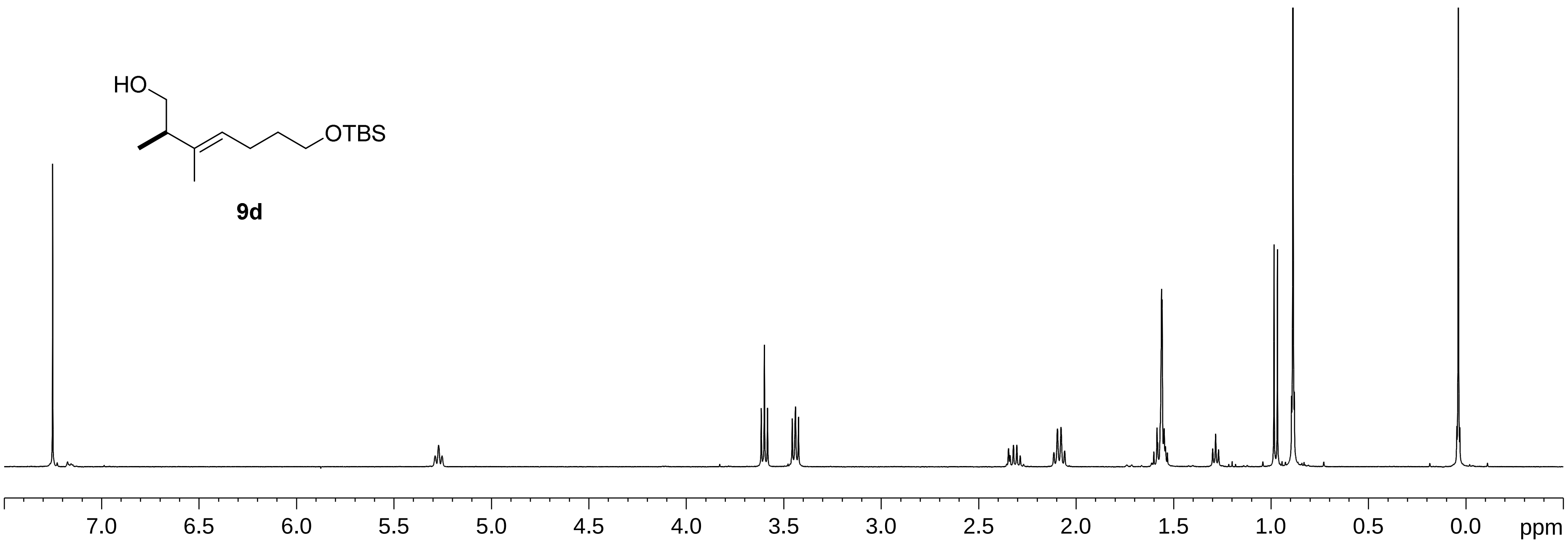
HO
OTBS
9d
7.0 6.5 6.0 5.5 5.0 4.5 4.0 3.5 3.0 2.5 2.0 1.5 1.0 0.5 0.0 ppm

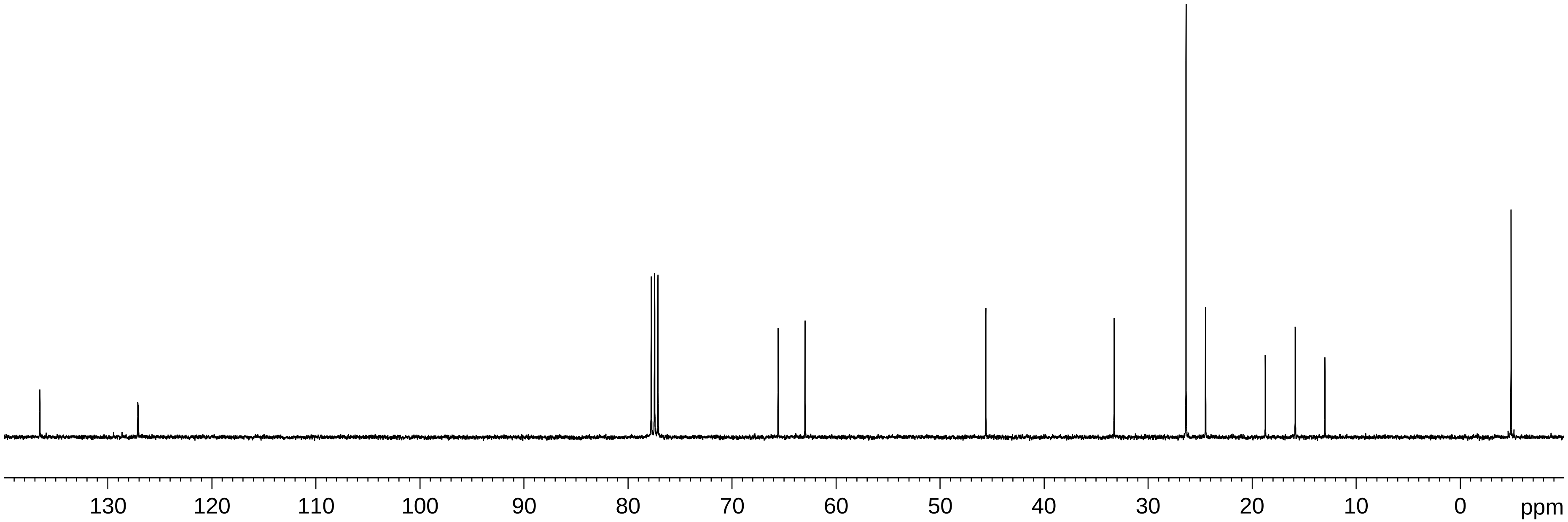
130 120 110 100 90 80 70 60 50 40 30 20 10 0 ppm

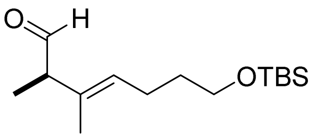
O
H
OTBS

10

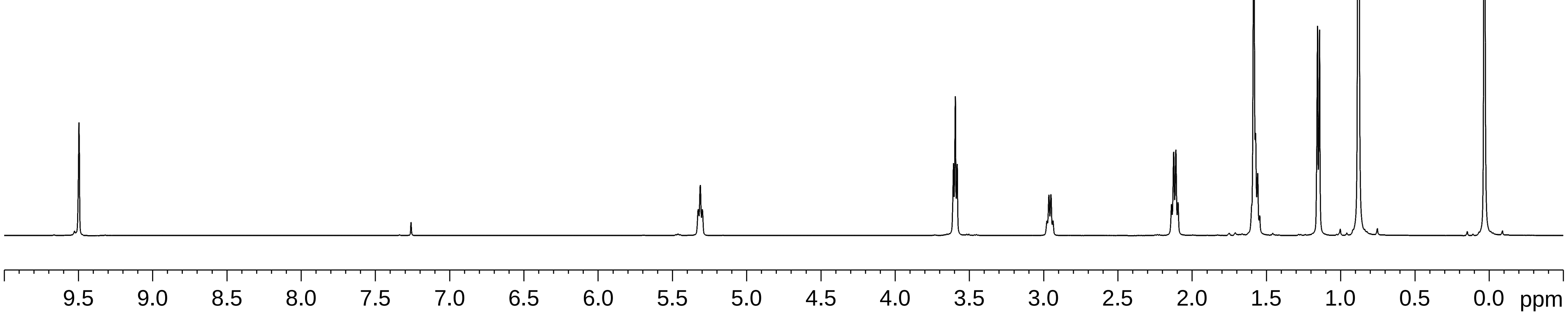
9.5 9.0 8.5 8.0 7.5 7.0 6.5 6.0 5.5 5.0 4.5 4.0 3.5 3.0 2.5 2.0 1.5 1.0 0.5 0.0 ppm

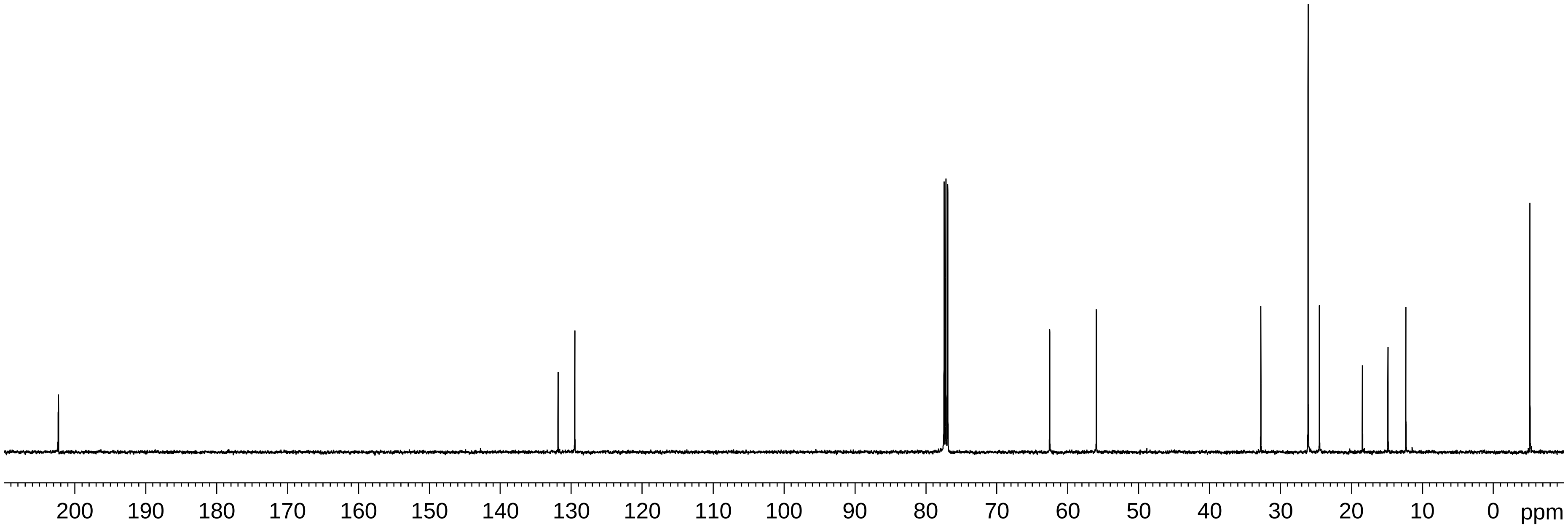
200 190 180 170 160 150 140 130 120 110 100 90 80 70 60 50 40 30 20 10 0 ppm

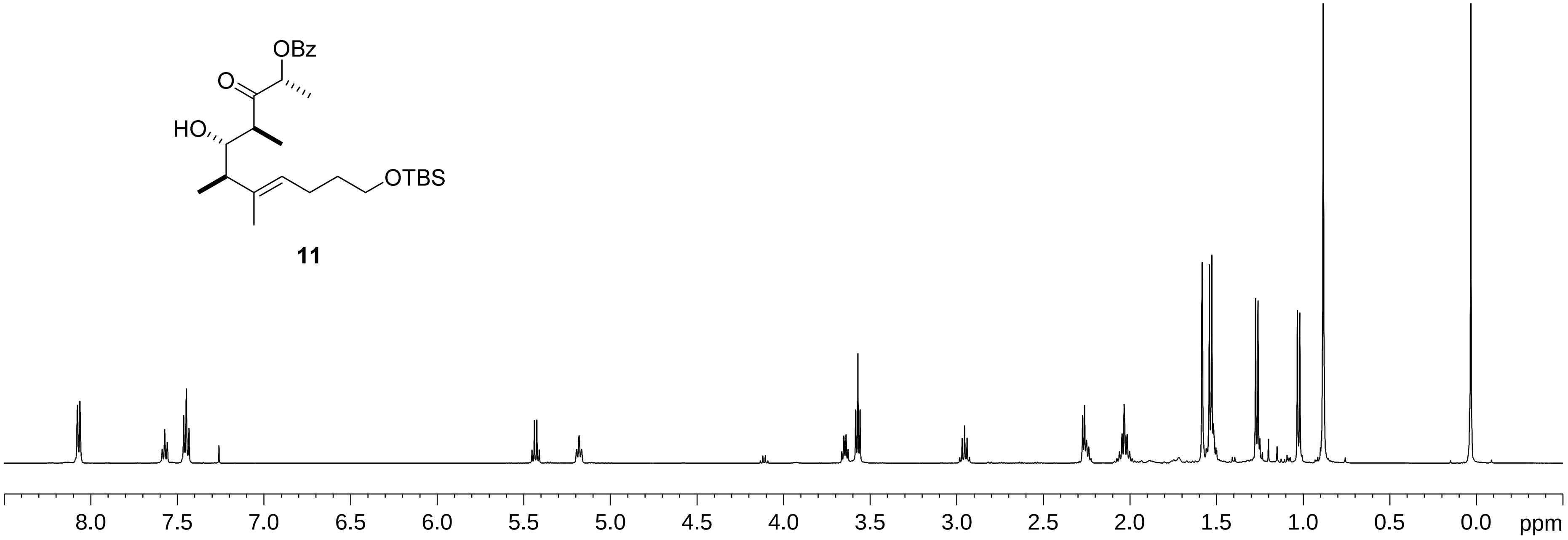
OBz
O
HO
OTBS
8.0
7.5
7.0
6.5
6.0
5.5
5.0
4.5
4.0
3.5
3.0
2.5
2.0
1.5
1.0
0.5
0.0
ppm

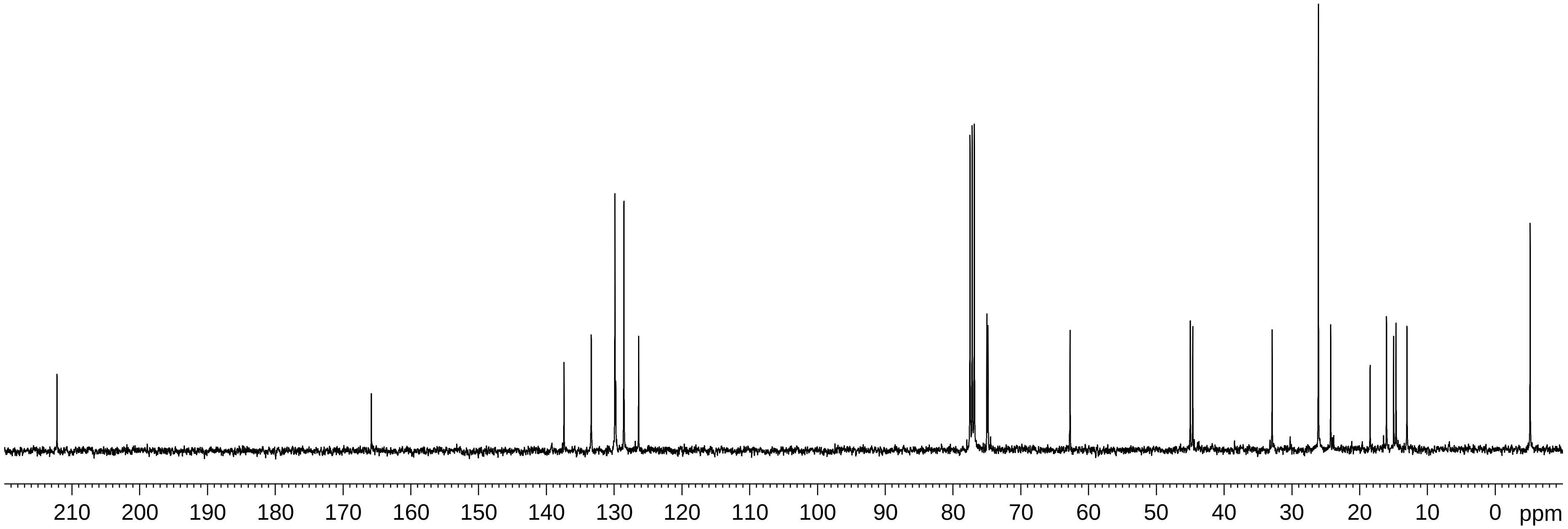
210
200
190
180
170
160
150
140
130
120
110
100
90
80
70
60
50
40
30
20
10
0
ppm

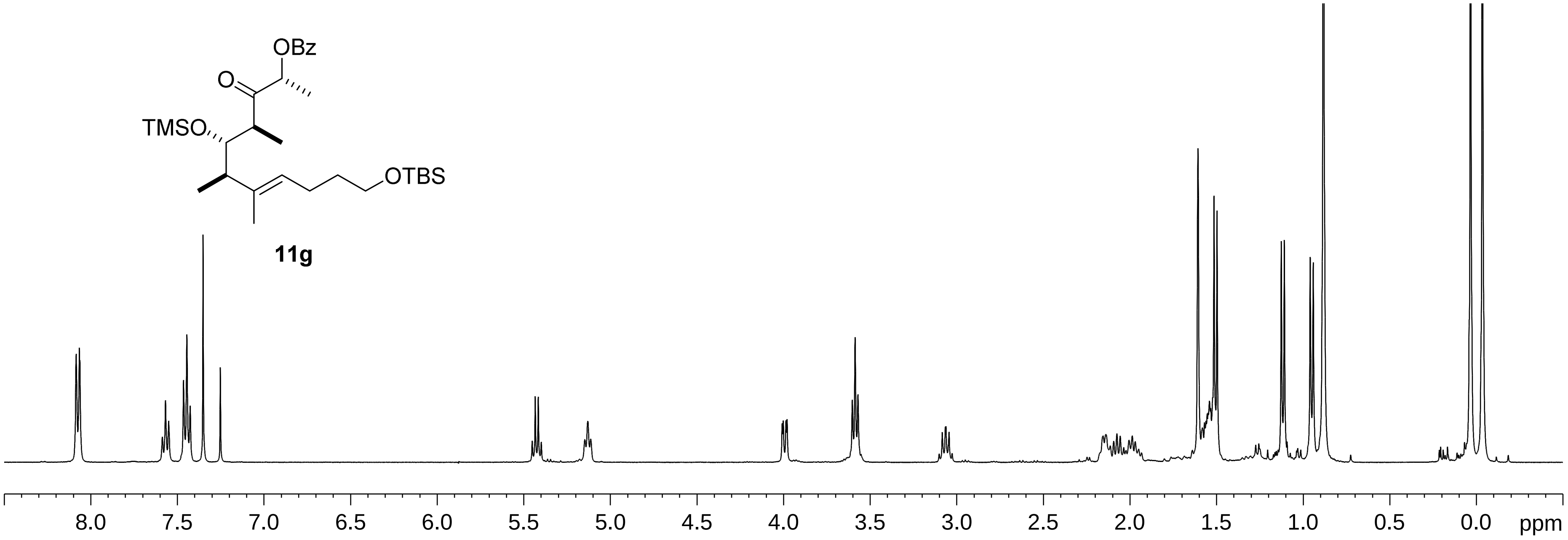

11g

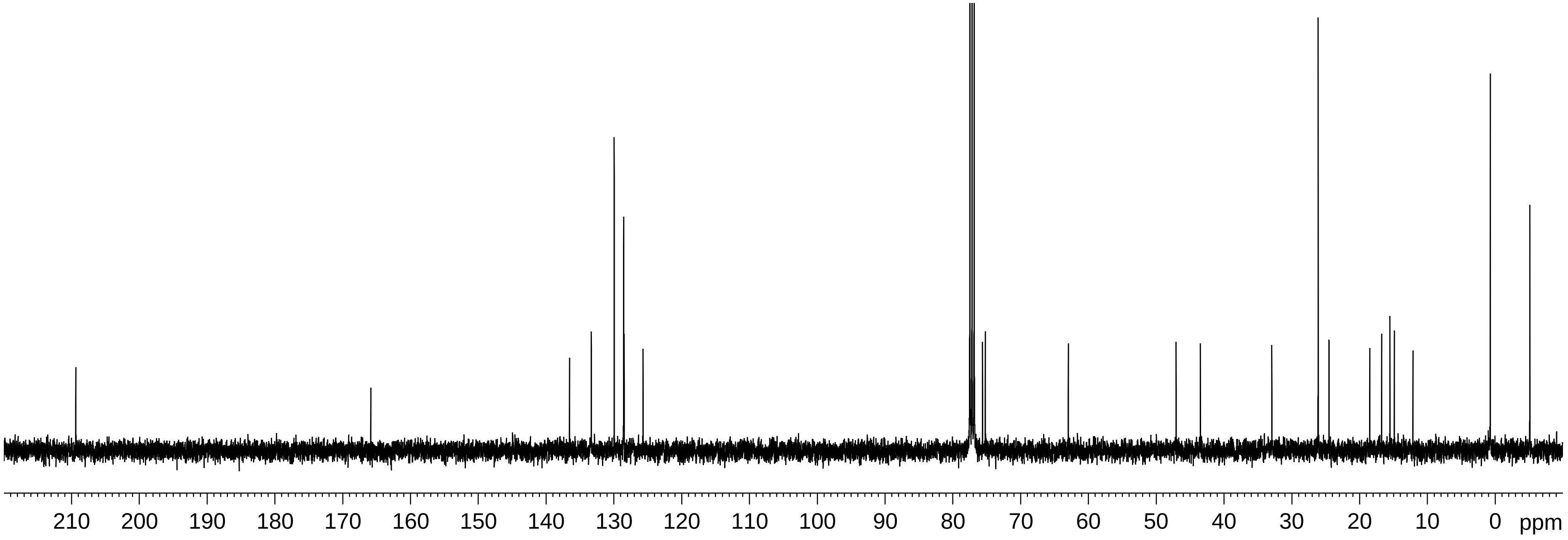

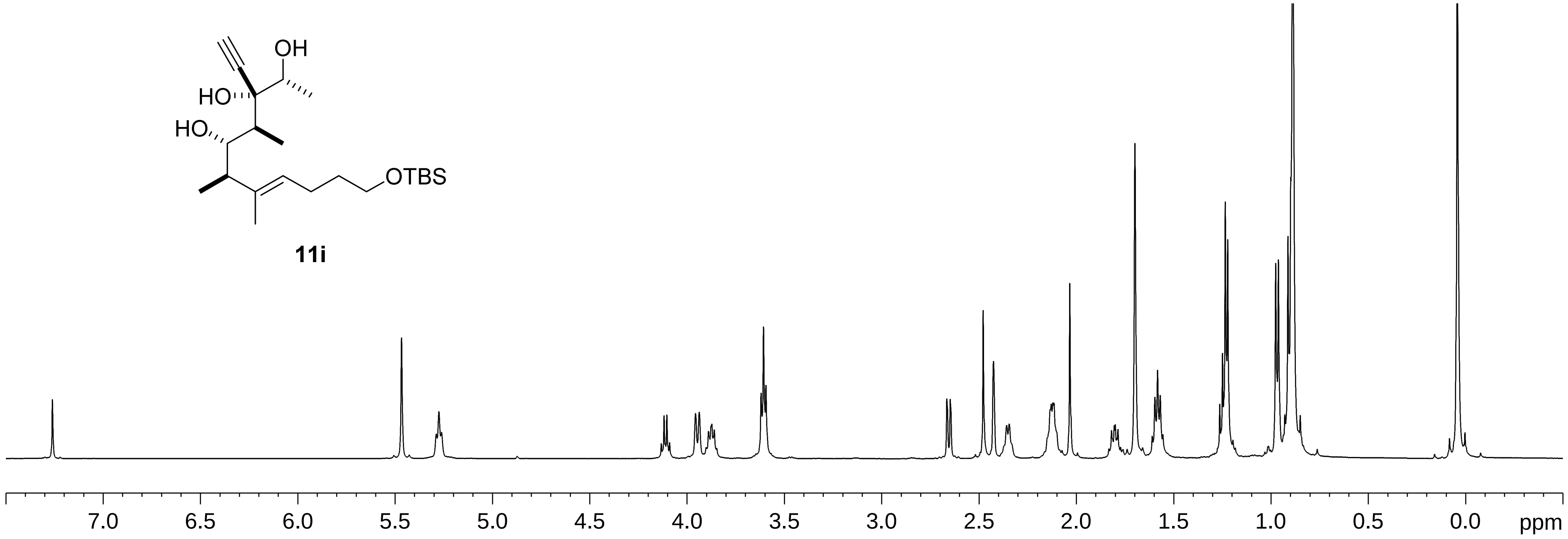
OH
HO
HO
OTBS
11i
7.0 6.5 6.0 5.5 5.0 4.5 4.0 3.5 3.0 2.5 2.0 1.5 1.0 0.5 0.0 ppm

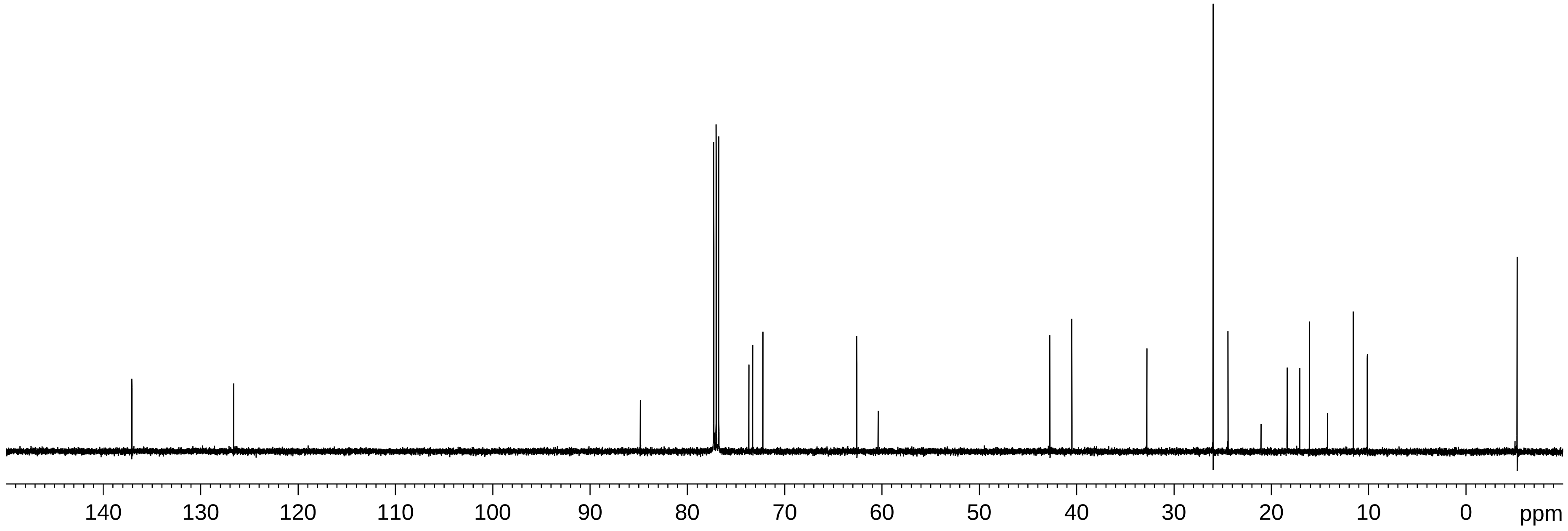
140 130 120 110 100 90 80 70 60 50 40 30 20 10 0 ppm

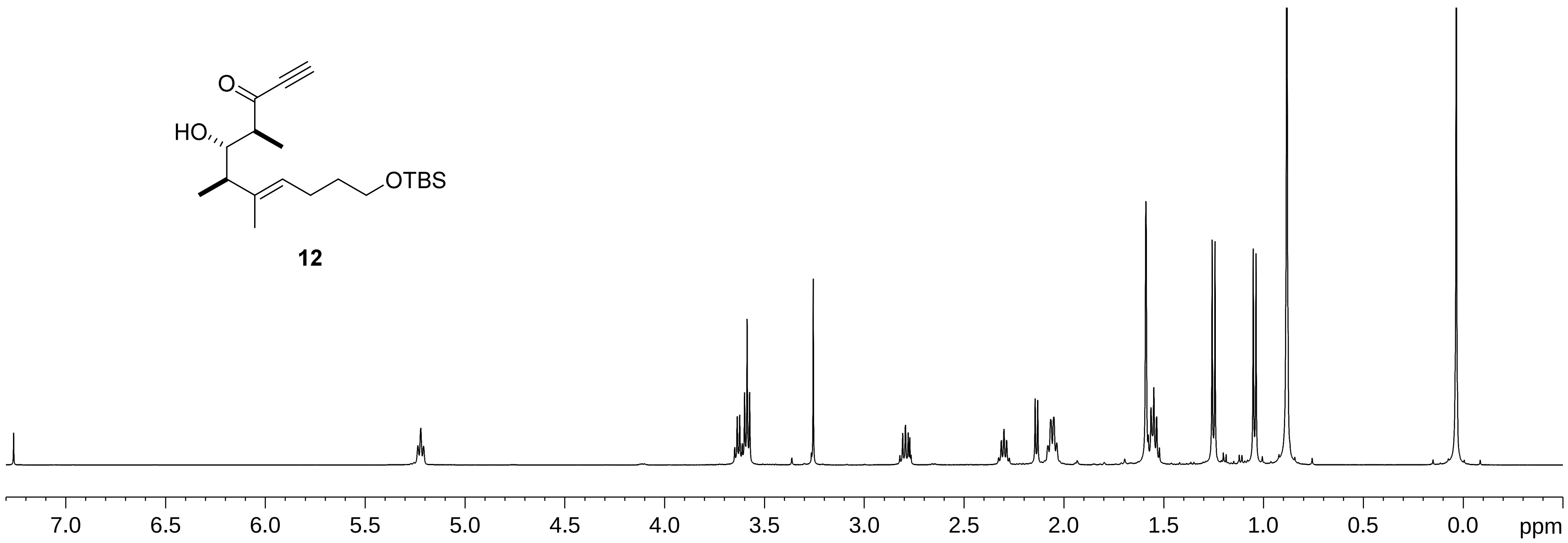
O
HO
OTBS
12
7.0
6.5
6.0
5.5
5.0
4.5
4.0
3.5
3.0
2.5
2.0
1.5
1.0
0.5
0.0
ppm

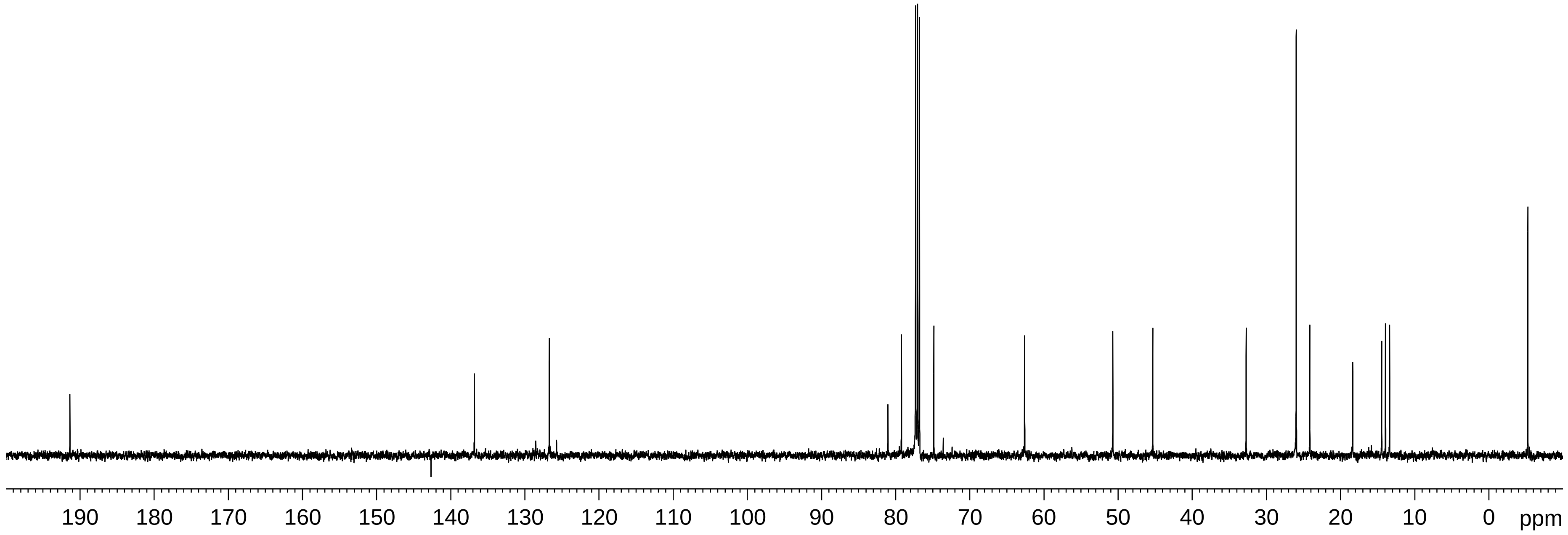
190
180
170
160
150
140
130
120
110
100
90
80
70
60
50
40
30
20
10
0
ppm

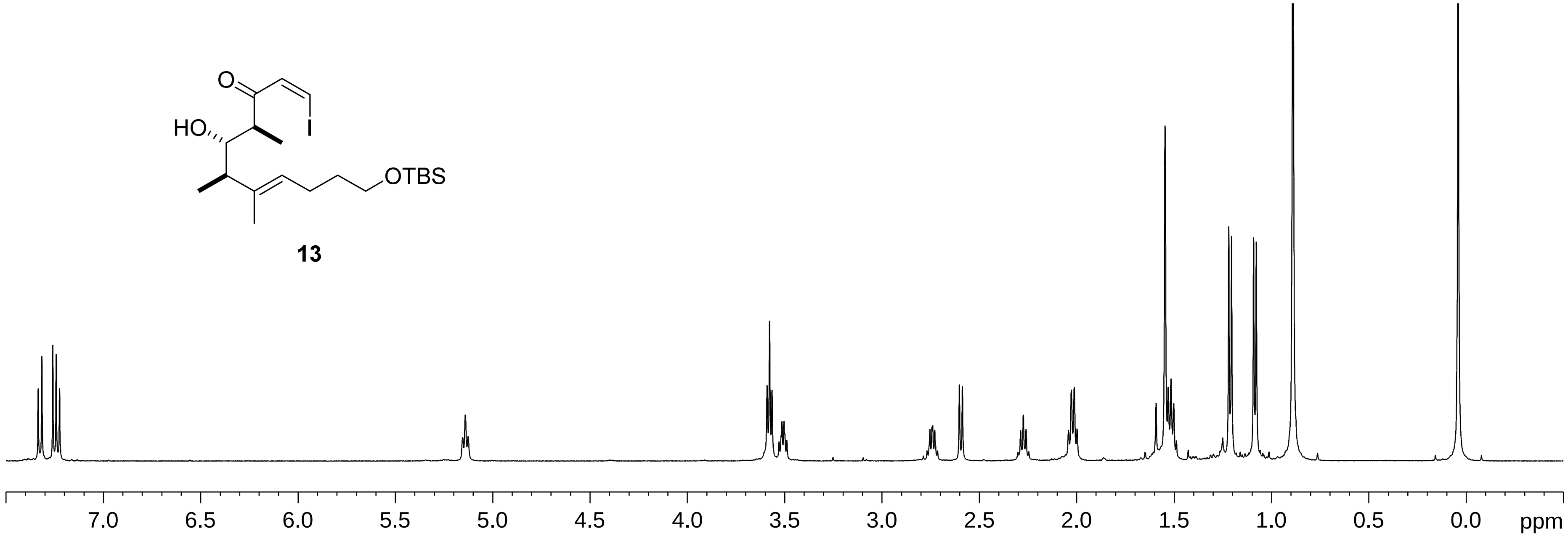
O
HO
I
OTBS
7.0 6.5 6.0 5.5 5.0 4.5 4.0 3.5 3.0 2.5 2.0 1.5 1.0 0.5 0.0 ppm

13

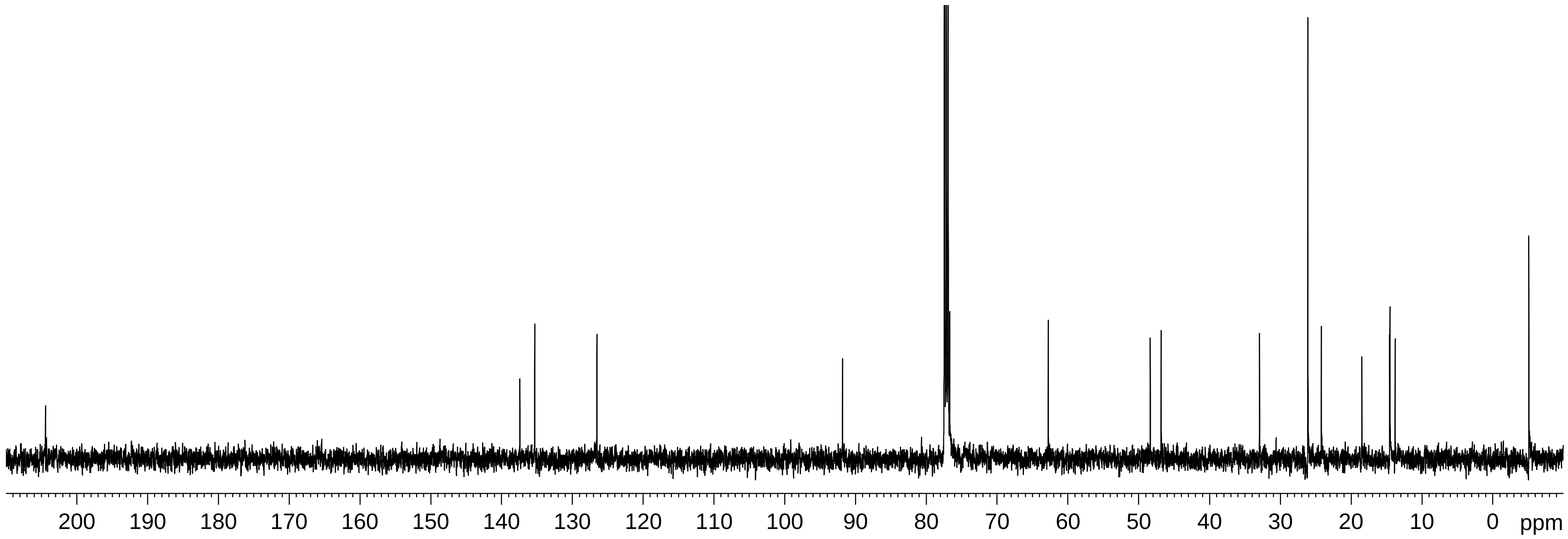
200 190 180 170 160 150 140 130 120 110 100 90 80 70 60 50 40 30 20 10 0 ppm

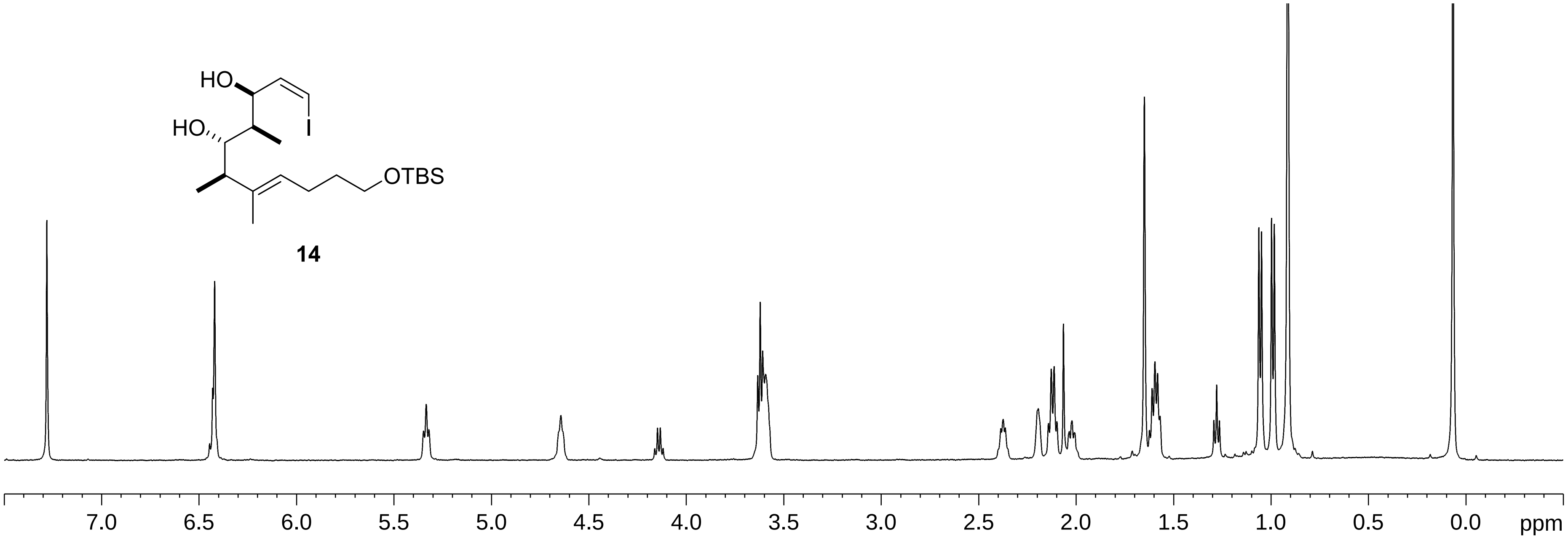
HO
HO
I
OTBS
14
7.0 6.5 6.0 5.5 5.0 4.5 4.0 3.5 3.0 2.5 2.0 1.5 1.0 0.5 0.0 ppm

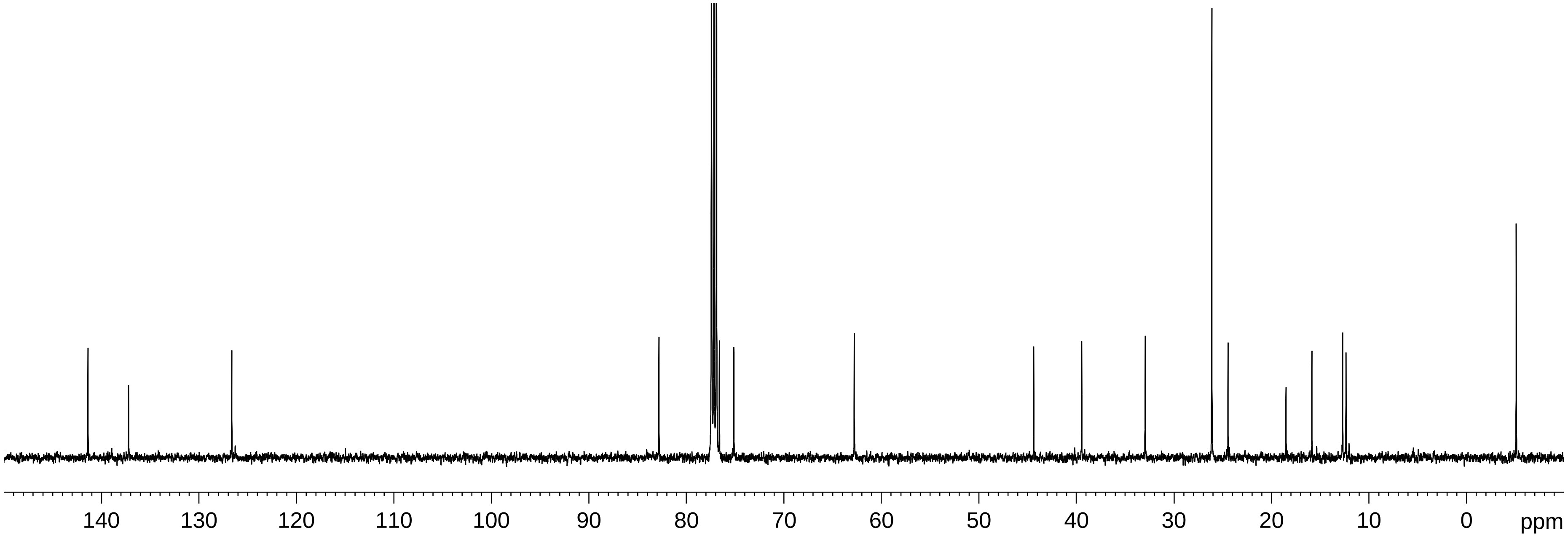
140 130 120 110 100 90 80 70 60 50 40 30 20 10 0 ppm

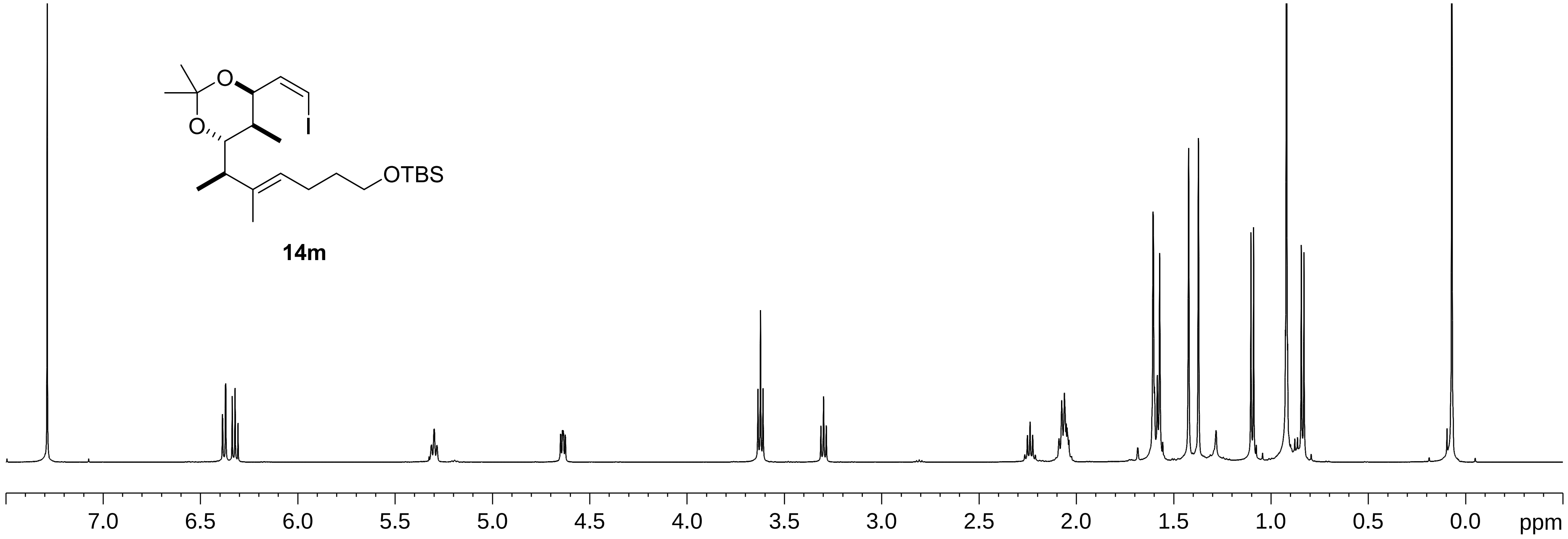
OTBS
I
O
O
14m
7.0 6.5 6.0 5.5 5.0 4.5 4.0 3.5 3.0 2.5 2.0 1.5 1.0 0.5 0.0 ppm

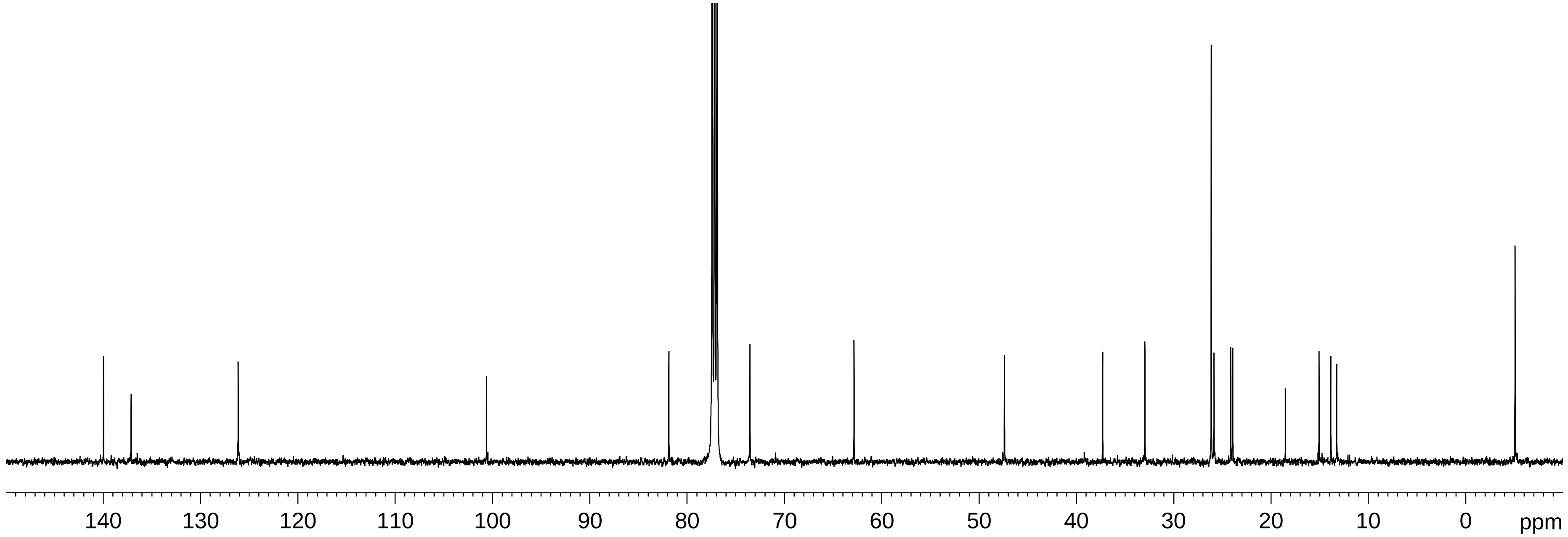
140 130 120 110 100 90 80 70 60 50 40 30 20 10 0 ppm

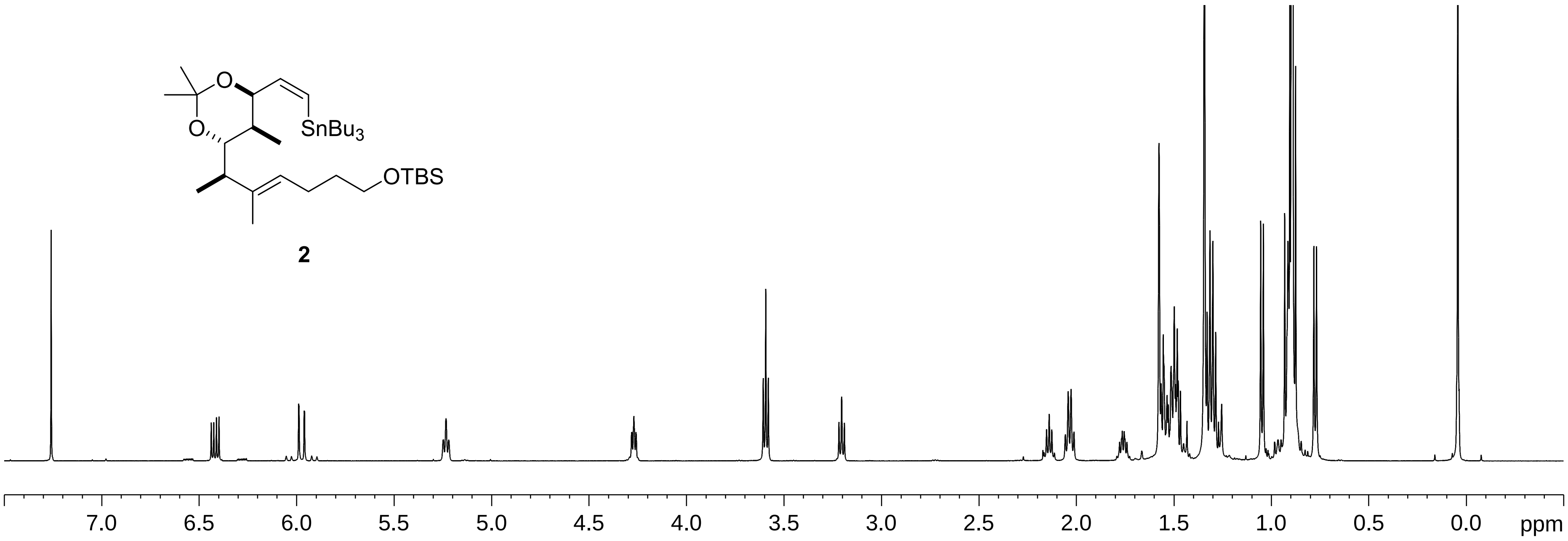
O
O
SnBu3
OTBS
2
7.0 6.5 6.0 5.5 5.0 4.5 4.0 3.5 3.0 2.5 2.0 1.5 1.0 0.5 0.0 ppm

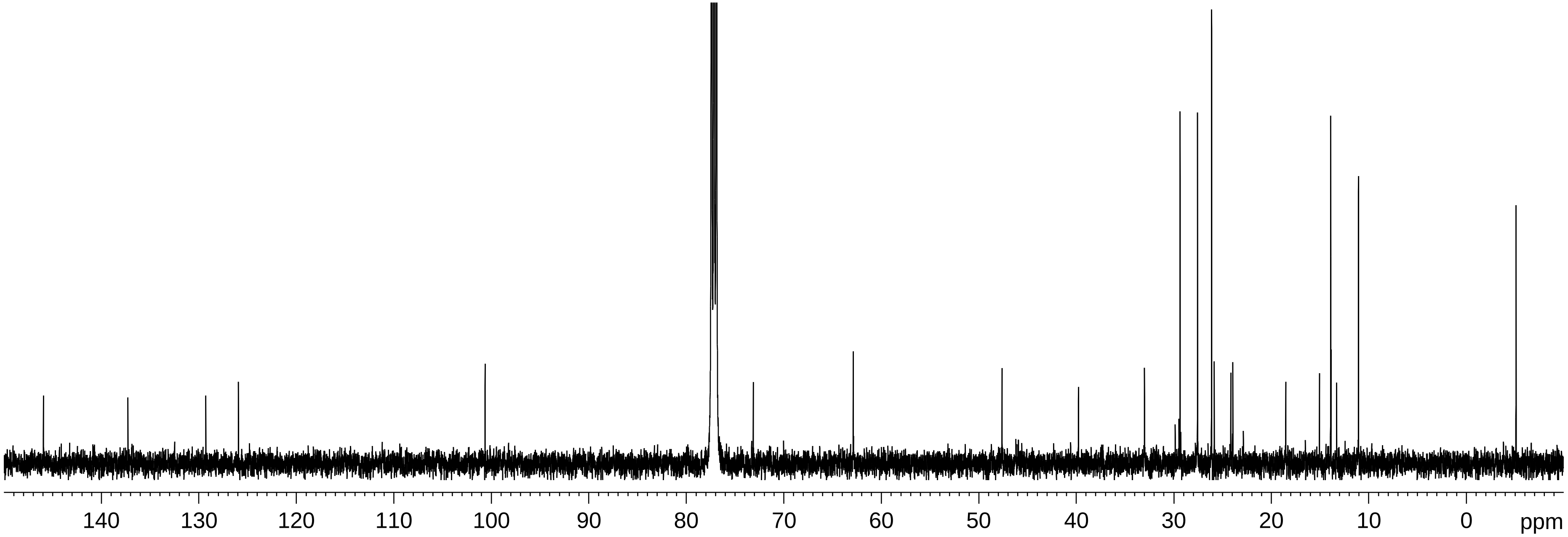
140 130 120 110 100 90 80 70 60 50 40 30 20 10 0 ppm

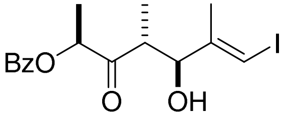
BzO
O
OH
I

16

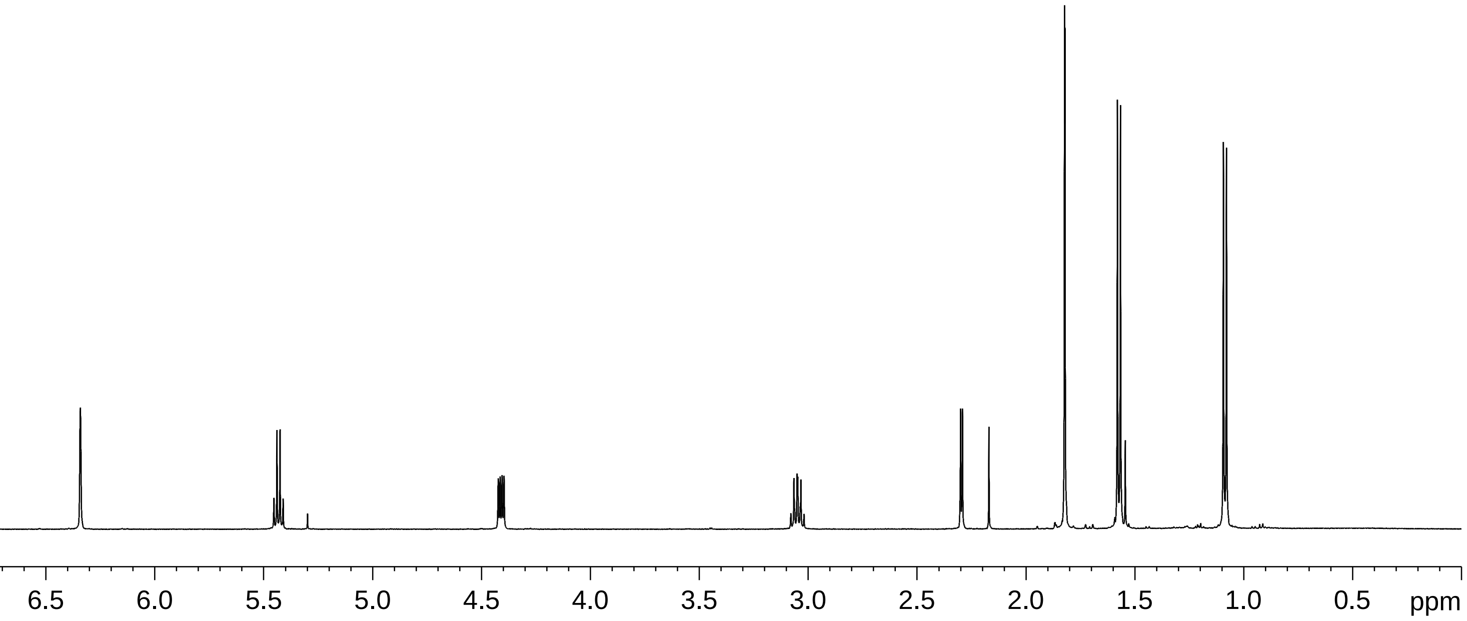
8.0 7.5 7.0 6.5 6.0 5.5 5.0 4.5 4.0 3.5 3.0 2.5 2.0 1.5 1.0 0.5 ppm

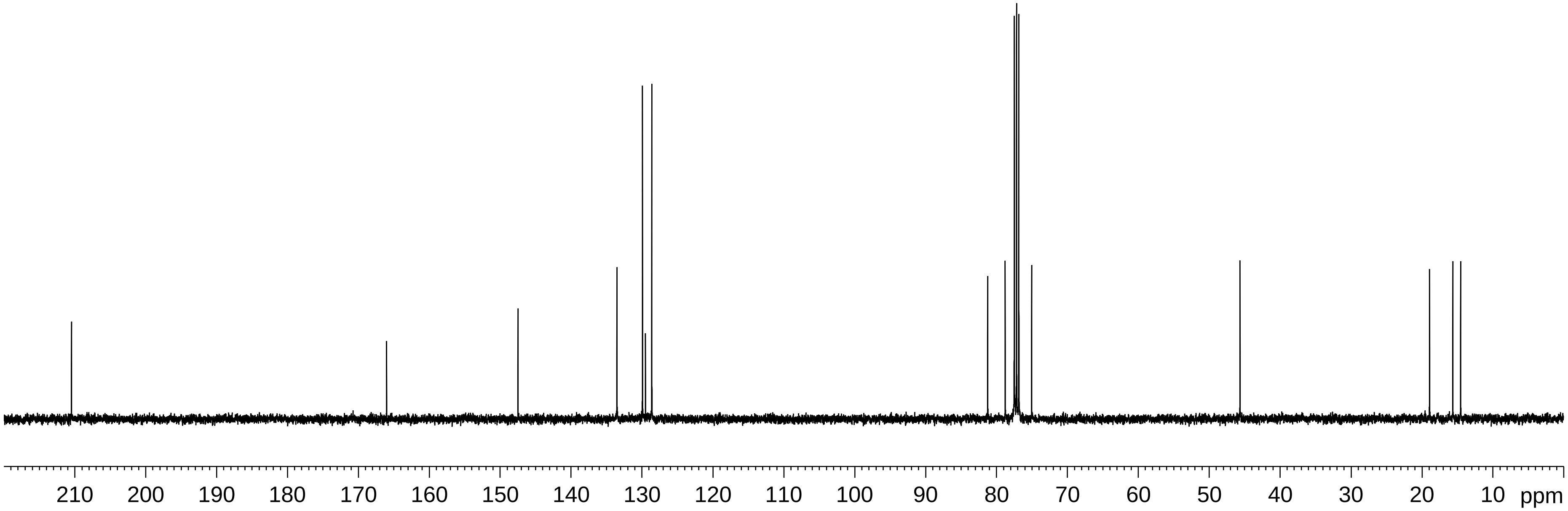
210 200 190 180 170 160 150 140 130 120 110 100 90 80 70 60 50 40 30 20 10 ppm

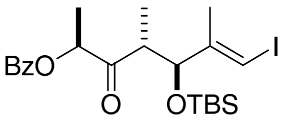
BzO
O
OTBS
I

16b

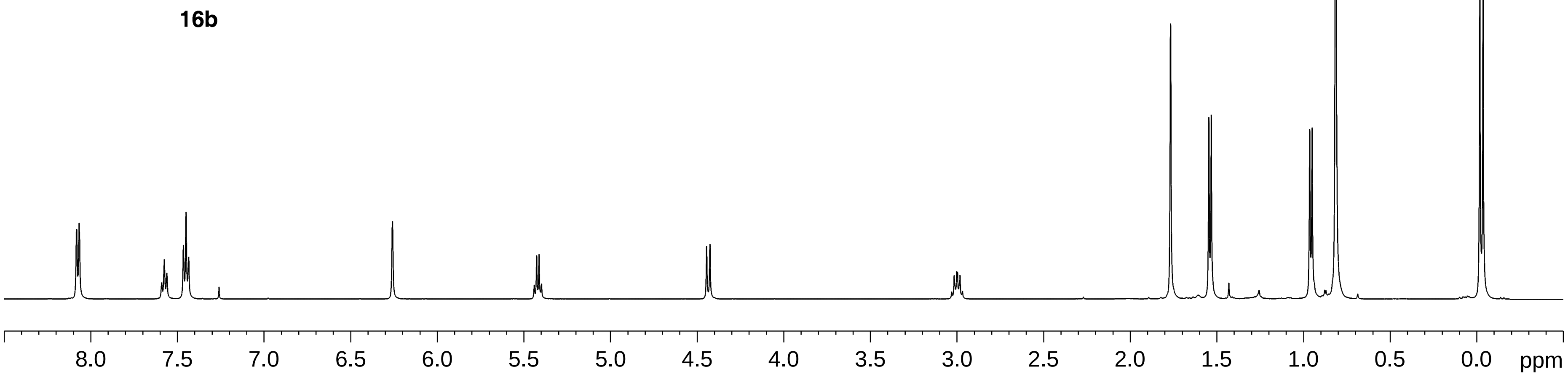
8.0 7.5 7.0 6.5 6.0 5.5 5.0 4.5 4.0 3.5 3.0 2.5 2.0 1.5 1.0 0.5 0.0 ppm

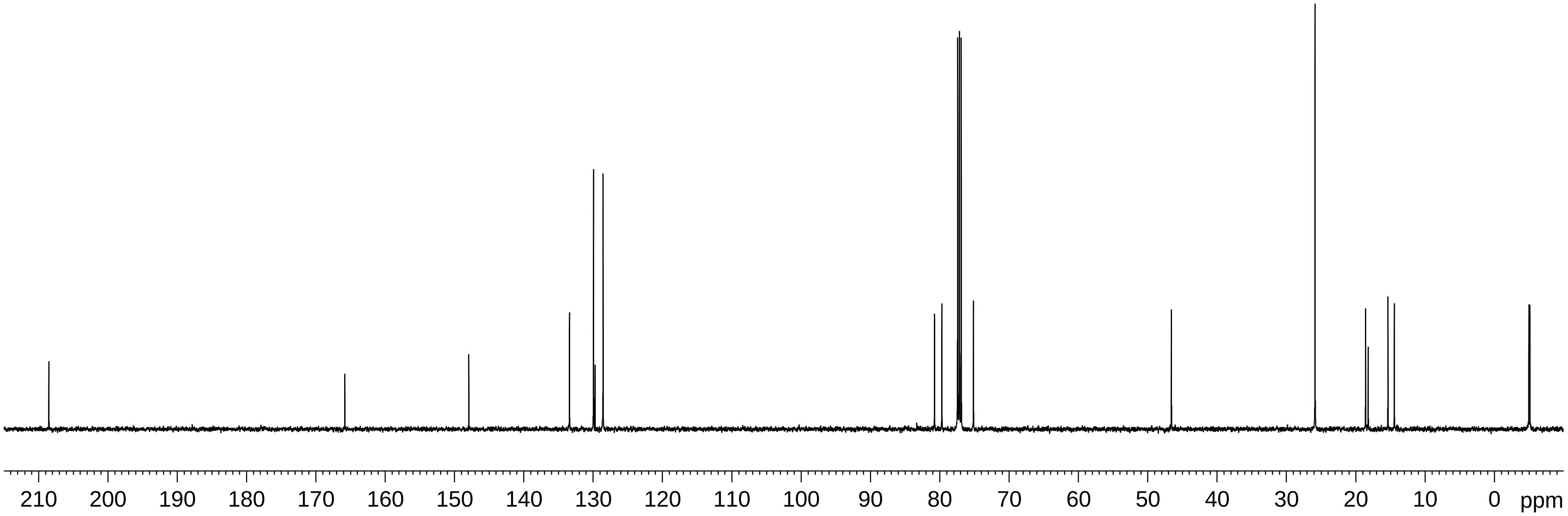
210 200 190 180 170 160 150 140 130 120 110 100 90 80 70 60 50 40 30 20 10 0 ppm

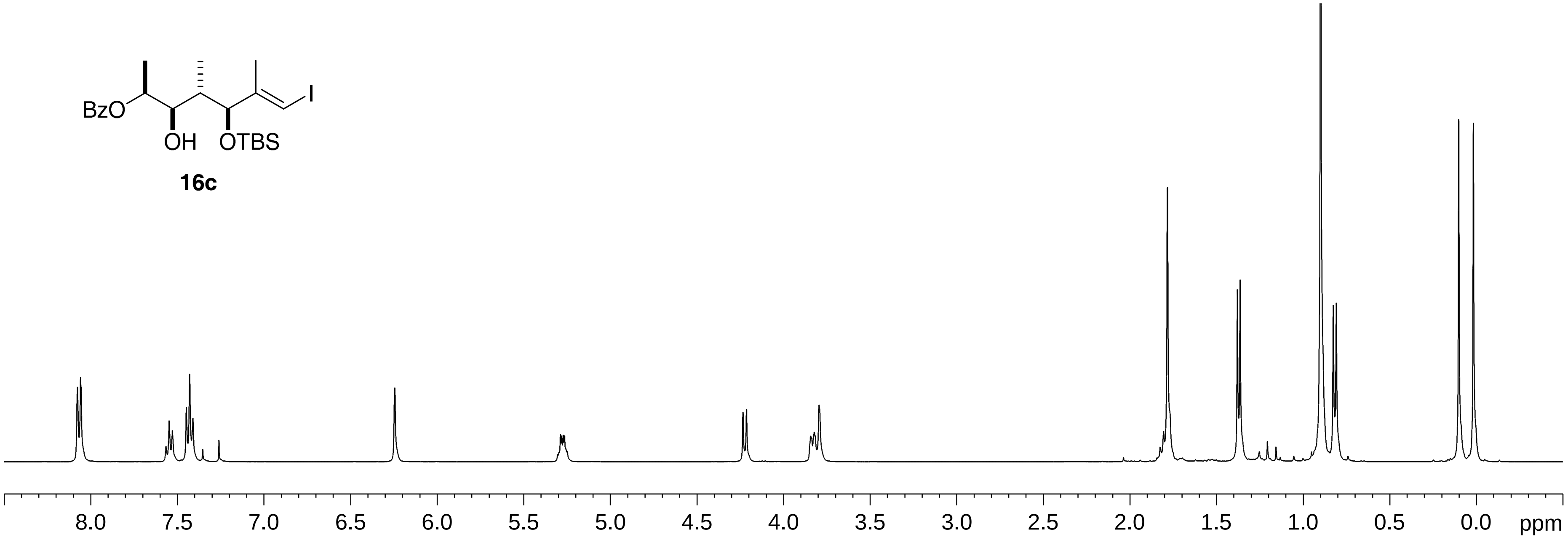
BzO
OH
OTBS
I
16c
8.0 7.5 7.0 6.5 6.0 5.5 5.0 4.5 4.0 3.5 3.0 2.5 2.0 1.5 1.0 0.5 0.0 ppm

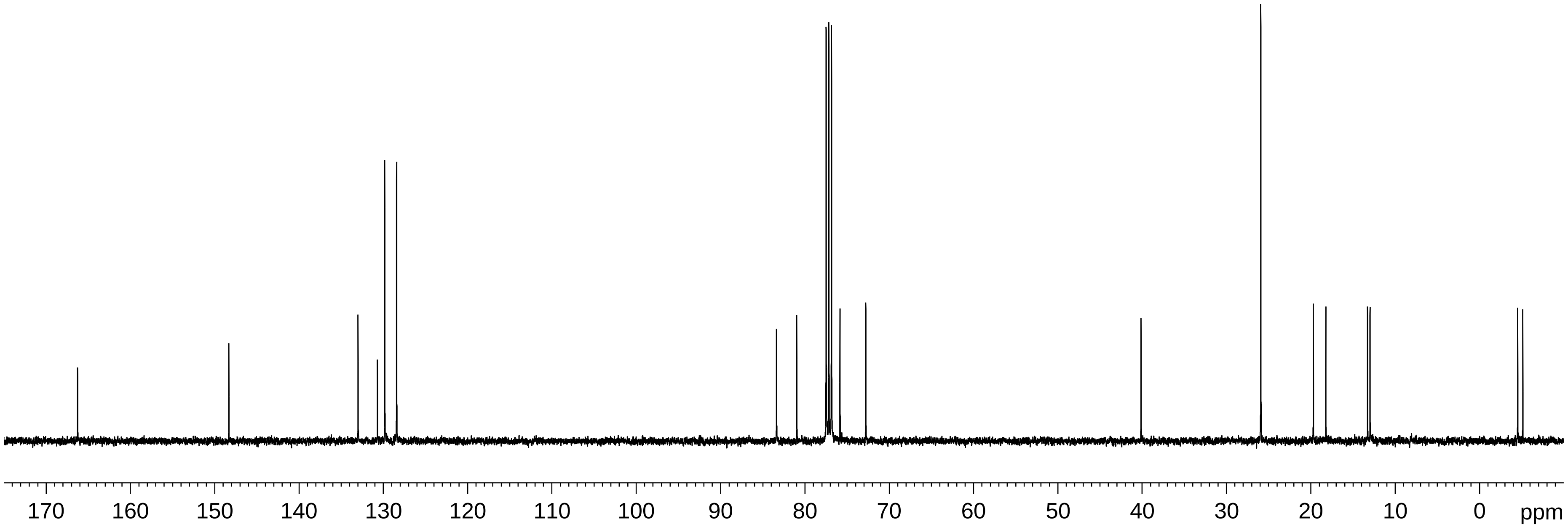
170 160 150 140 130 120 110 100 90 80 70 60 50 40 30 20 10 0 ppm

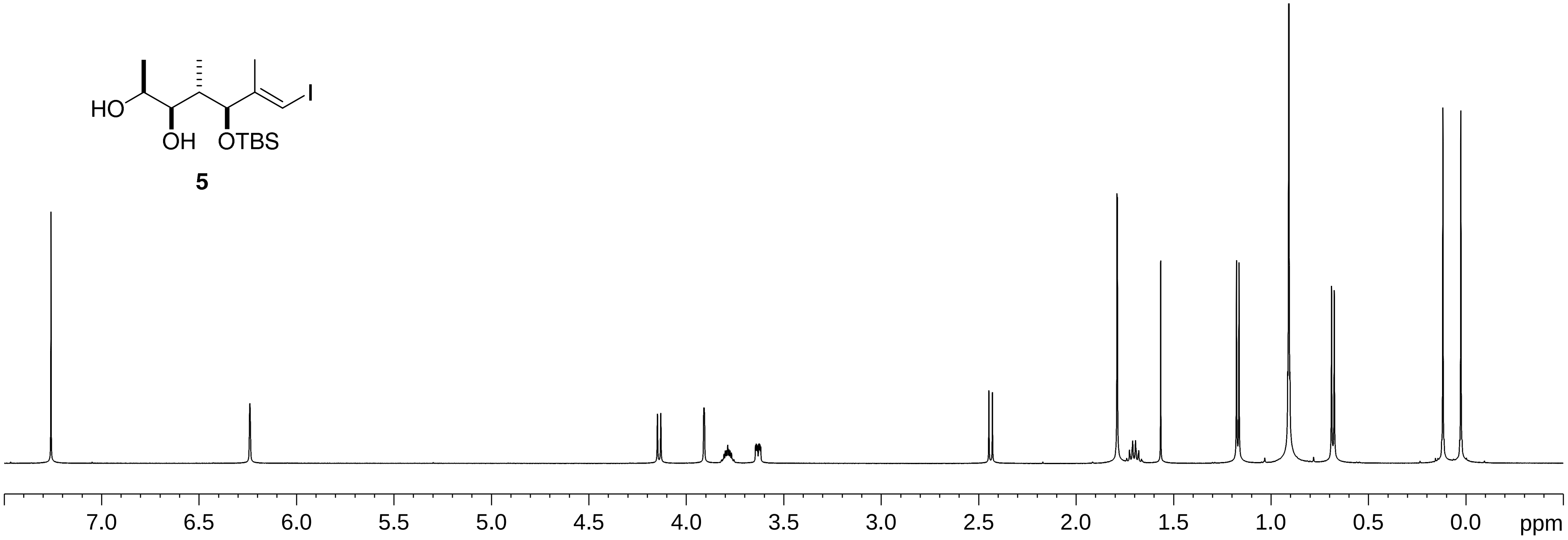
HO
OH
OTBS
I
5
7.0 6.5 6.0 5.5 5.0 4.5 4.0 3.5 3.0 2.5 2.0 1.5 1.0 0.5 0.0 ppm

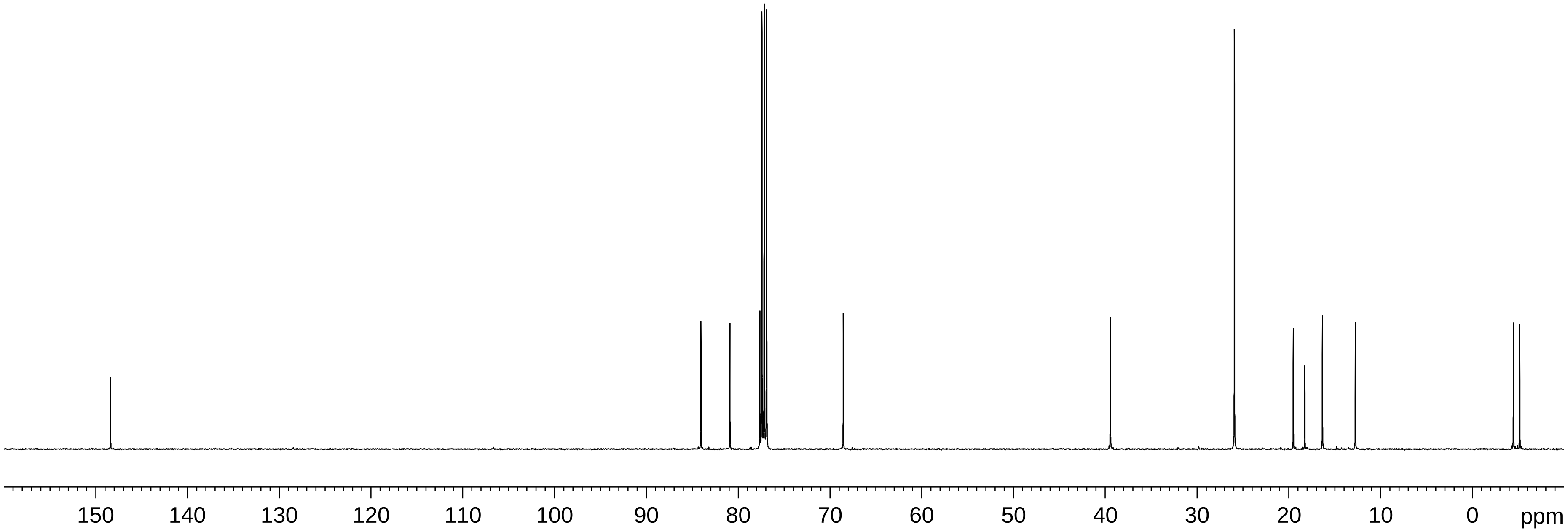
150 140 130 120 110 100 90 80 70 60 50 40 30 20 10 0 ppm

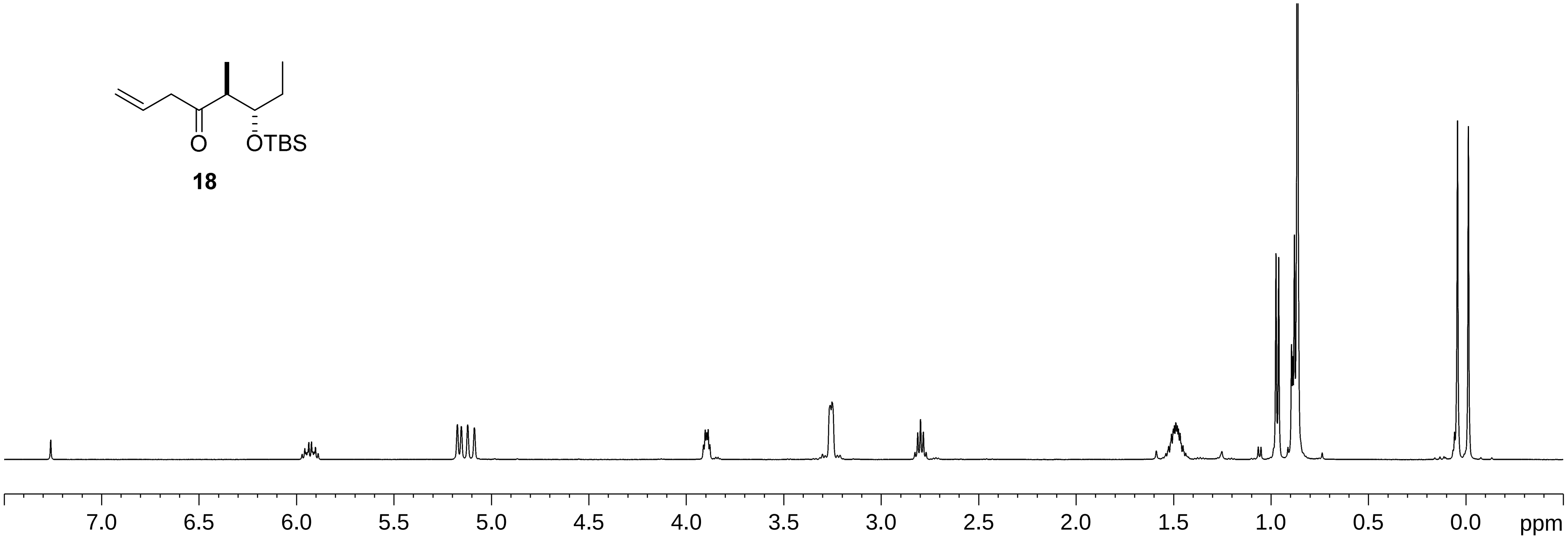
O
OTBS
18
7.0 6.5 6.0 5.5 5.0 4.5 4.0 3.5 3.0 2.5 2.0 1.5 1.0 0.5 0.0 ppm

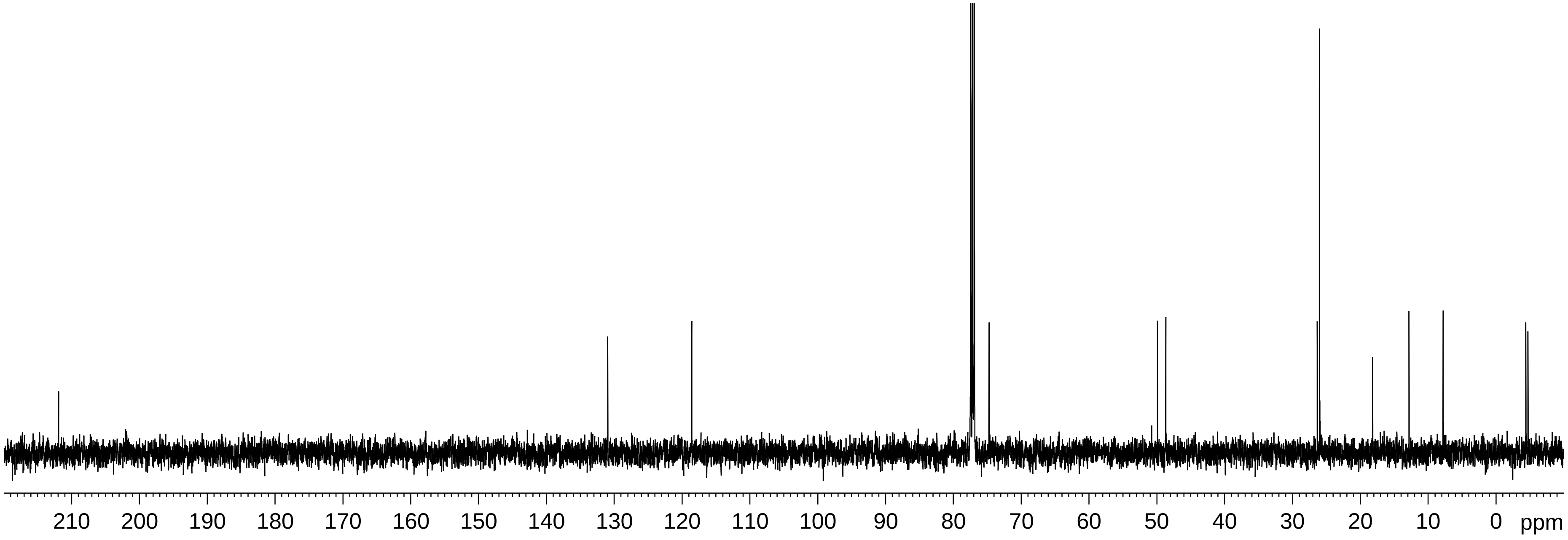
210 200 190 180 170 160 150 140 130 120 110 100 90 80 70 60 50 40 30 20 10 0 ppm

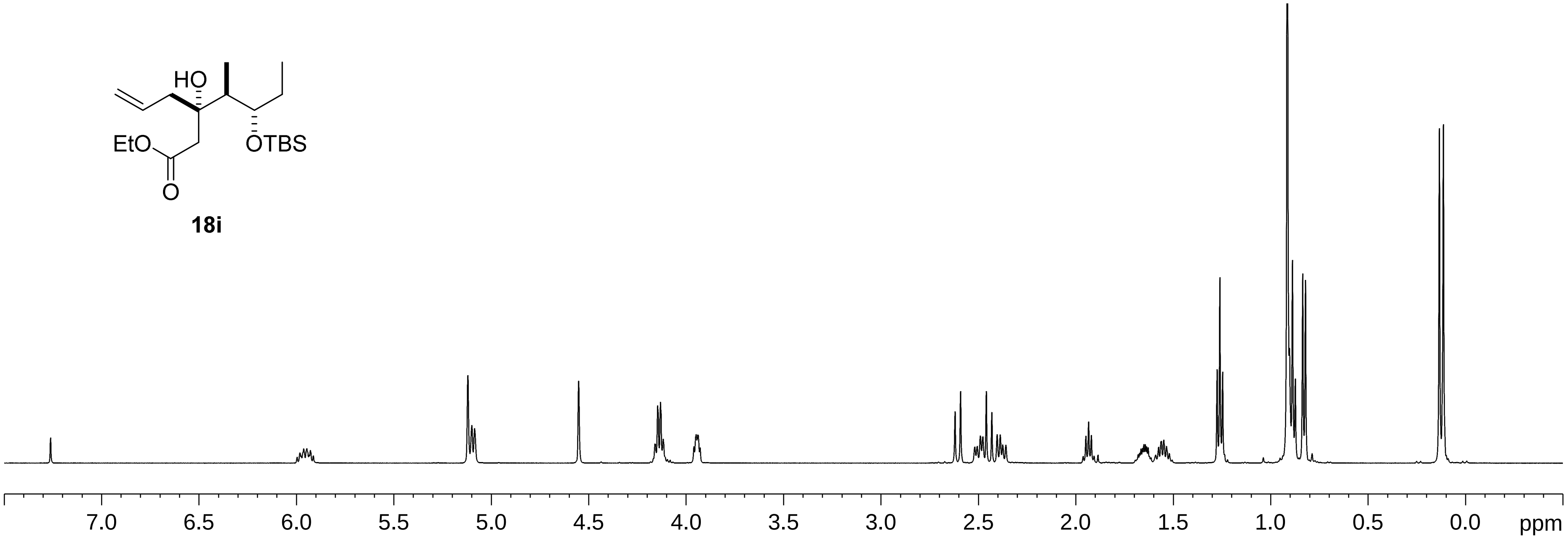
HO
EtO
O
OTBS
7.0 6.5 6.0 5.5 5.0 4.5 4.0 3.5 3.0 2.5 2.0 1.5 1.0 0.5 0.0 ppm

18i

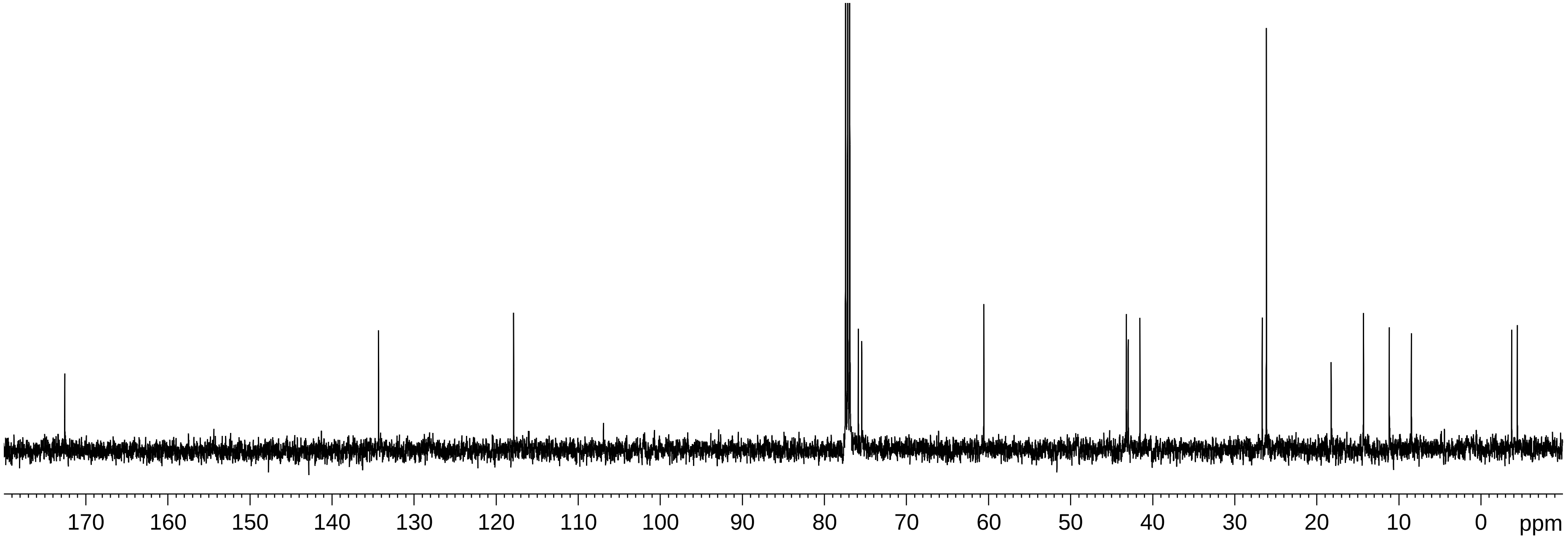
170 160 150 140 130 120 110 100 90 80 70 60 50 40 30 20 10 0 ppm

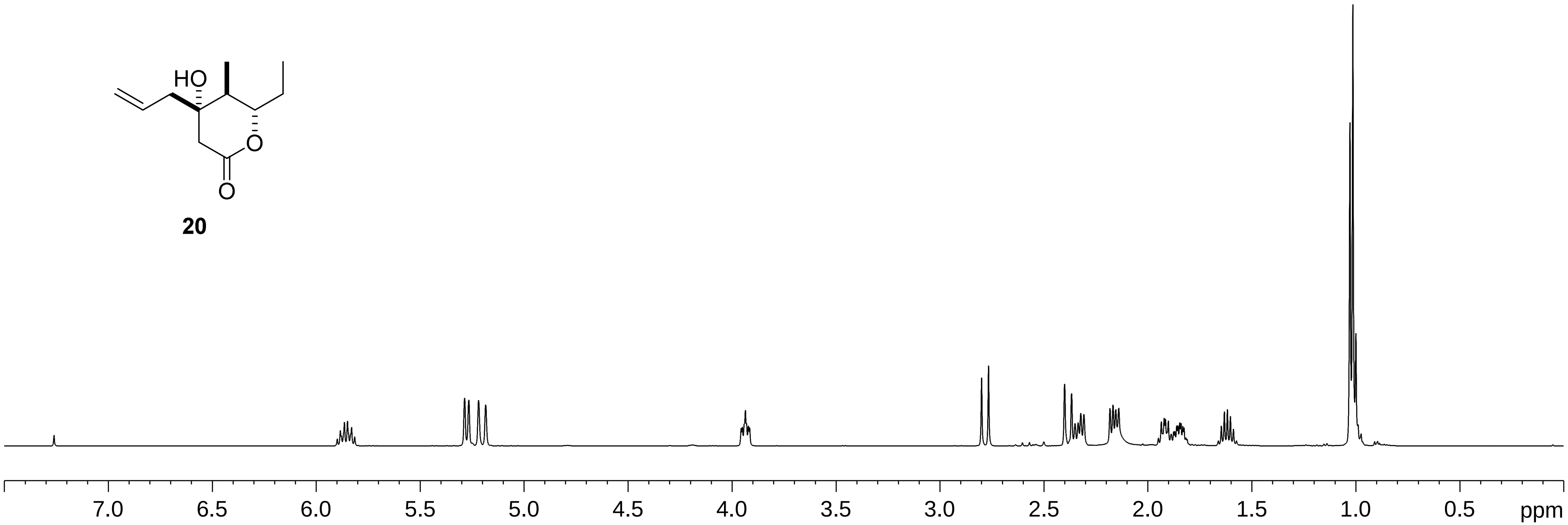
HO
O
O
20
7.0 6.5 6.0 5.5 5.0 4.5 4.0 3.5 3.0 2.5 2.0 1.5 1.0 0.5 ppm

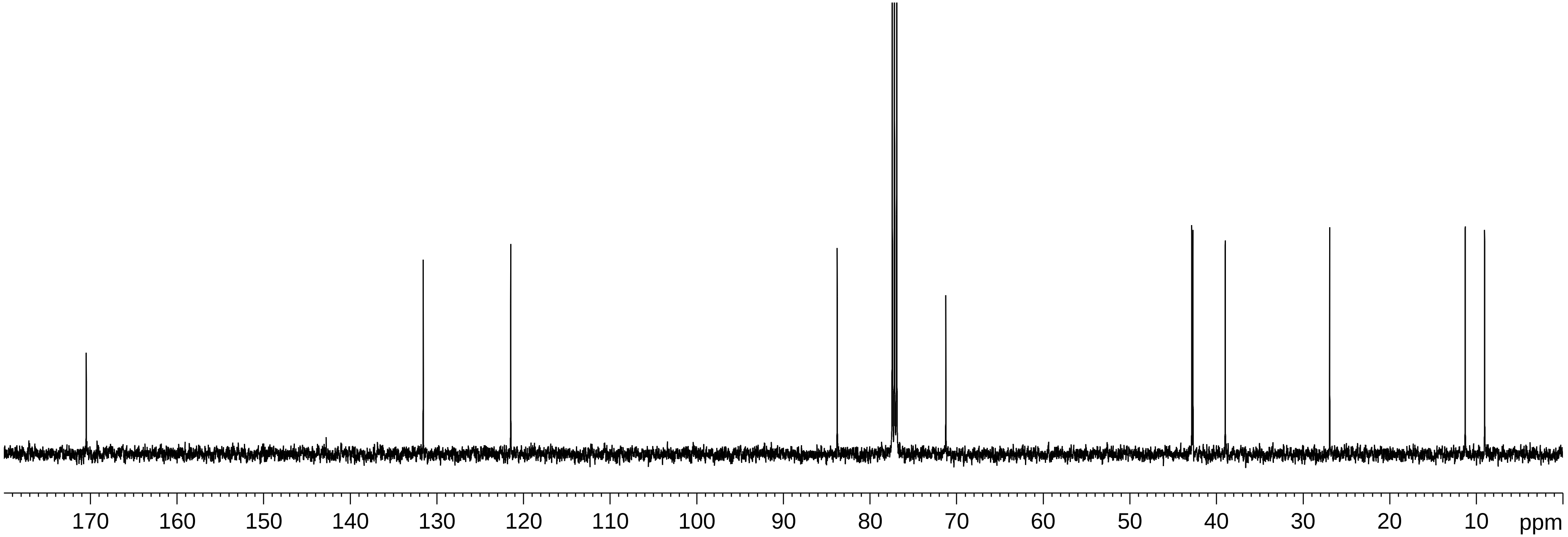
170 160 150 140 130 120 110 100 90 80 70 60 50 40 30 20 10 ppm

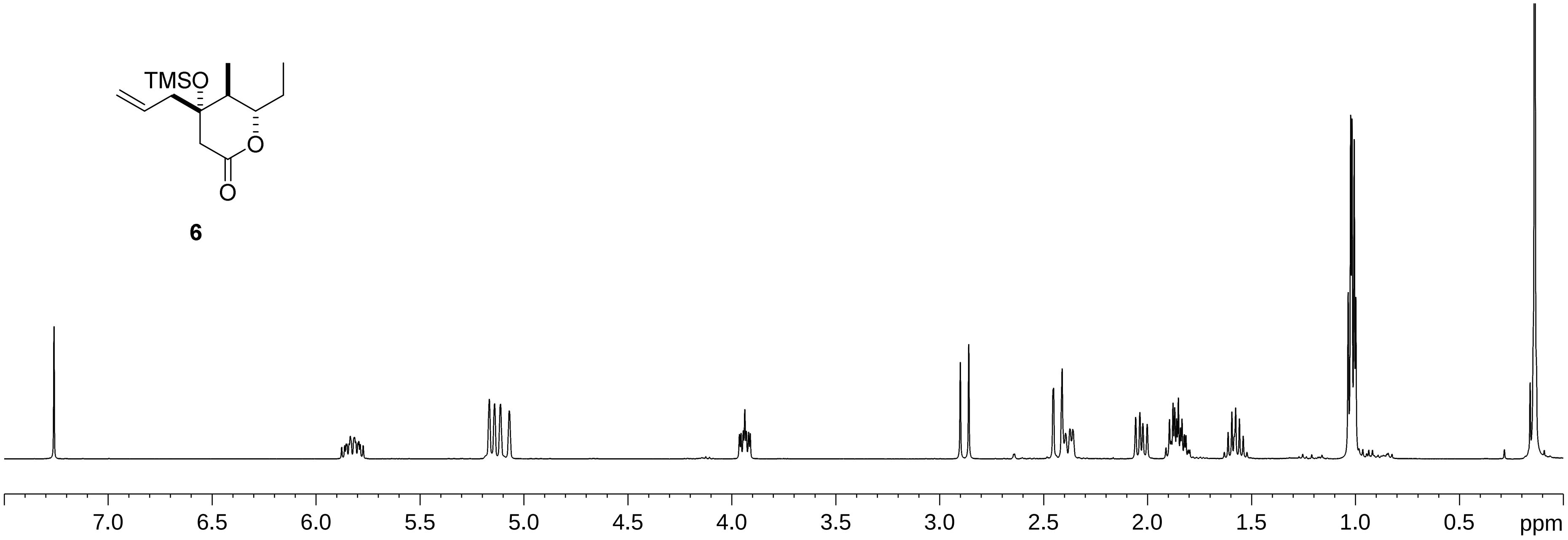
TMSO
O
O
6
7.0 6.5 6.0 5.5 5.0 4.5 4.0 3.5 3.0 2.5 2.0 1.5 1.0 0.5 ppm

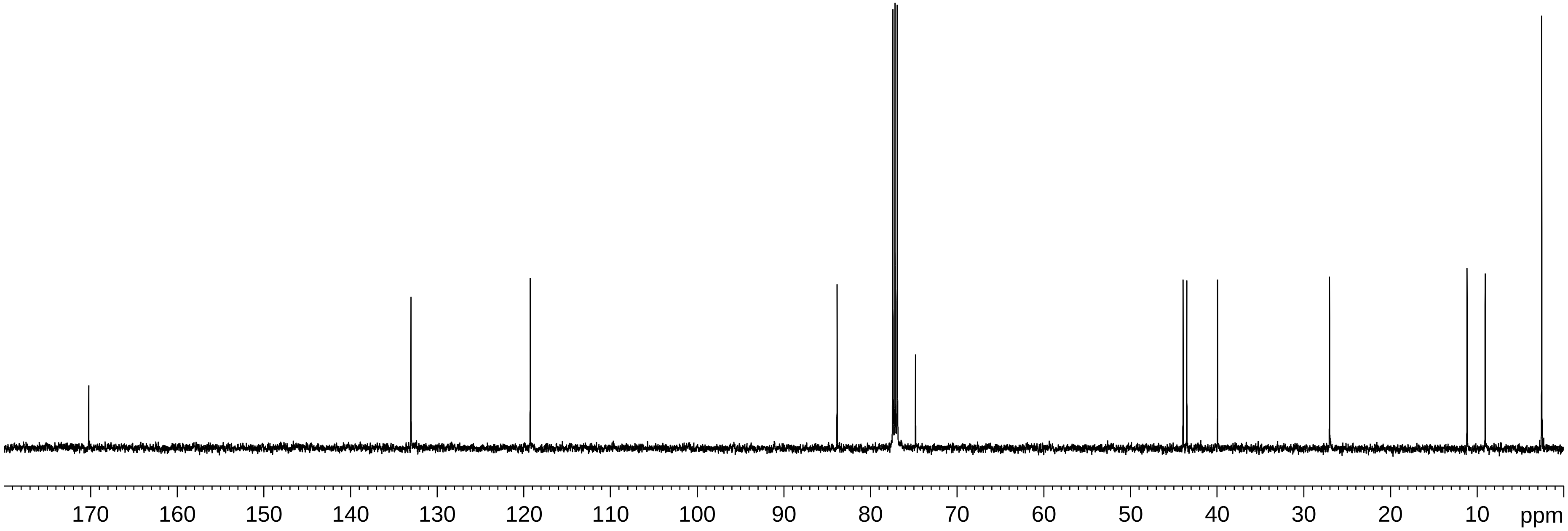
170 160 150 140 130 120 110 100 90 80 70 60 50 40 30 20 10 ppm

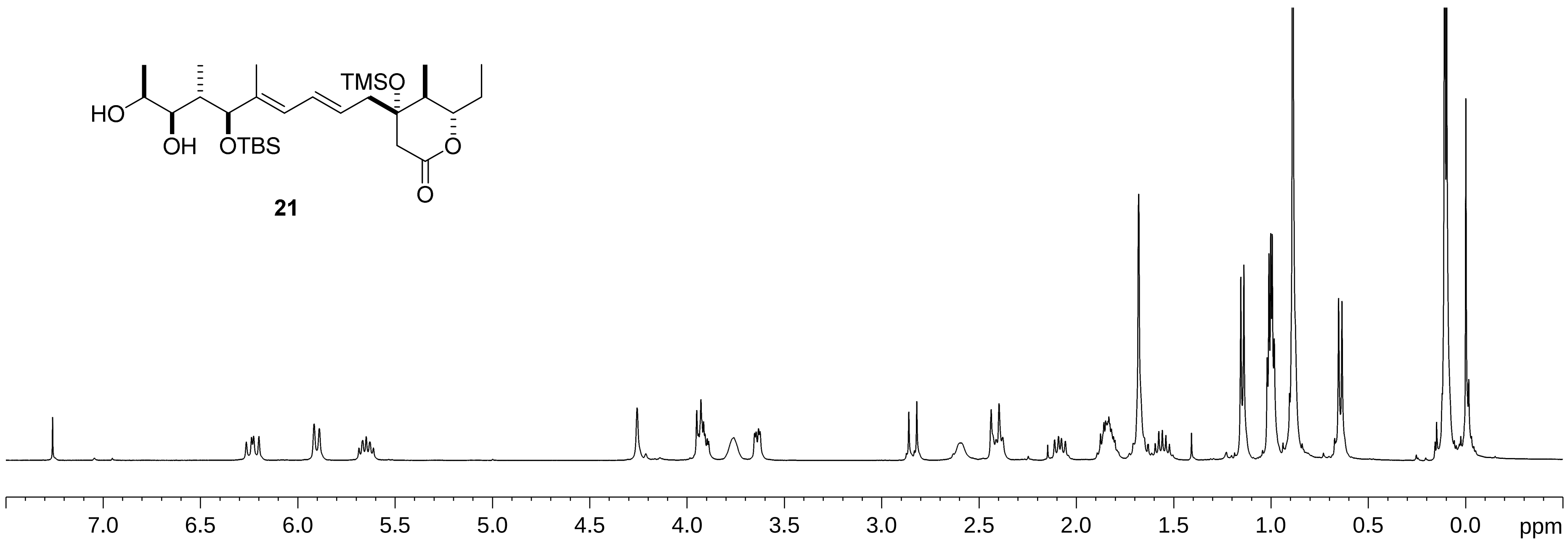
HO
OH
OTBS
TMSO
O
O
7.0 6.5 6.0 5.5 5.0 4.5 4.0 3.5 3.0 2.5 2.0 1.5 1.0 0.5 0.0 ppm

21

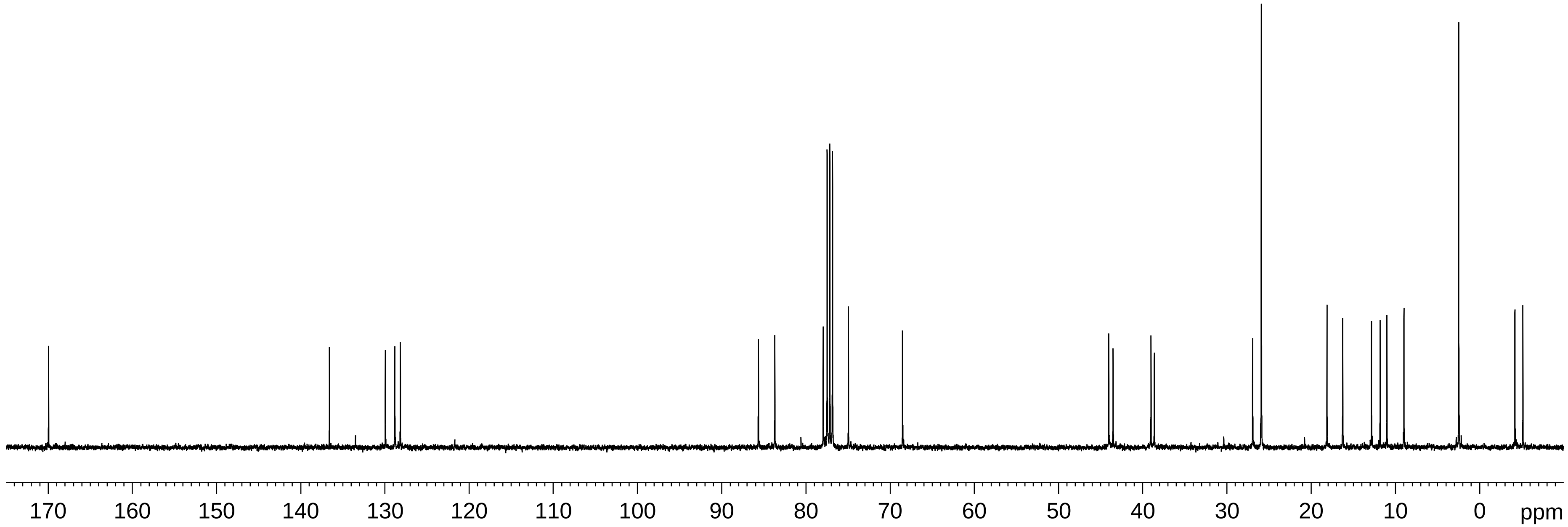
170 160 150 140 130 120 110 100 90 80 70 60 50 40 30 20 10 0 ppm

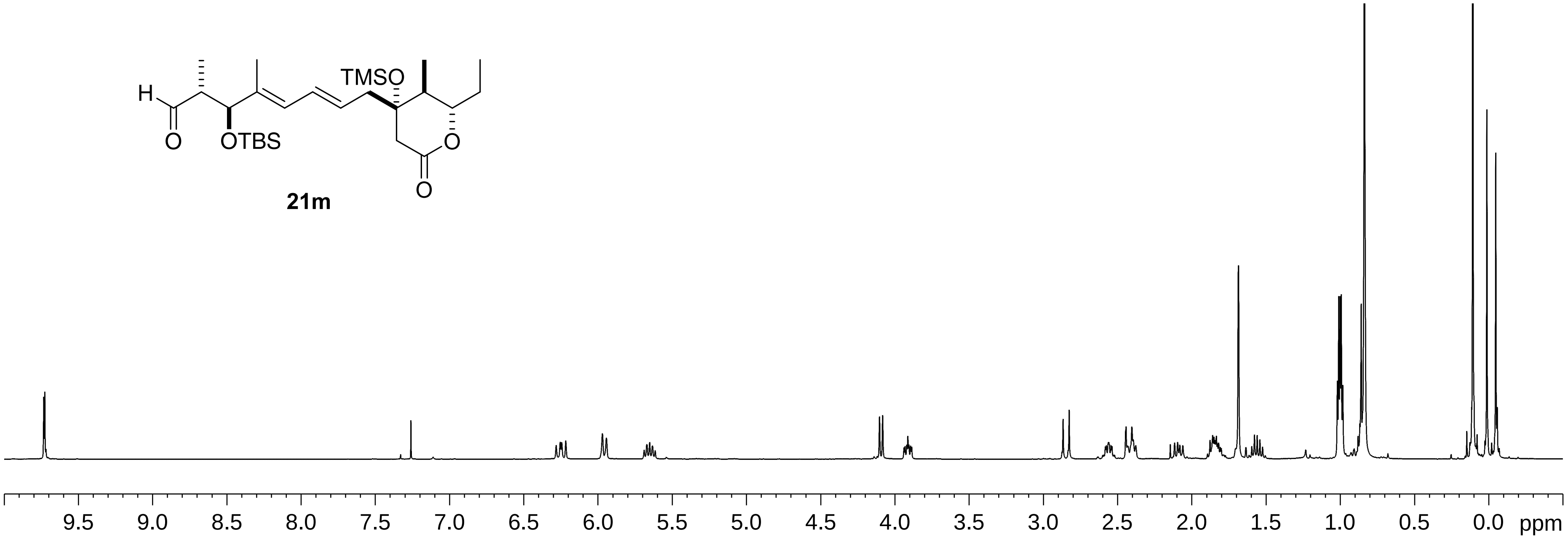
H
O
OTBS
TMSO
O
O
21m
9.5 9.0 8.5 8.0 7.5 7.0 6.5 6.0 5.5 5.0 4.5 4.0 3.5 3.0 2.5 2.0 1.5 1.0 0.5 0.0 ppm

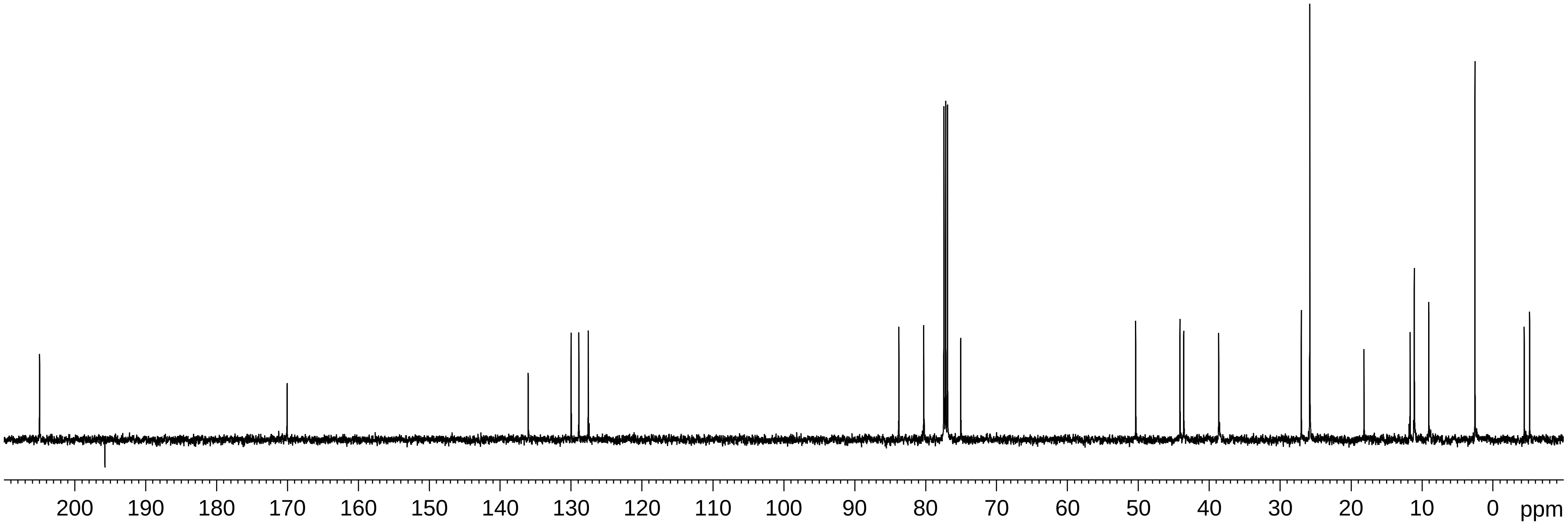
200 190 180 170 160 150 140 130 120 110 100 90 80 70 60 50 40 30 20 10 0 ppm

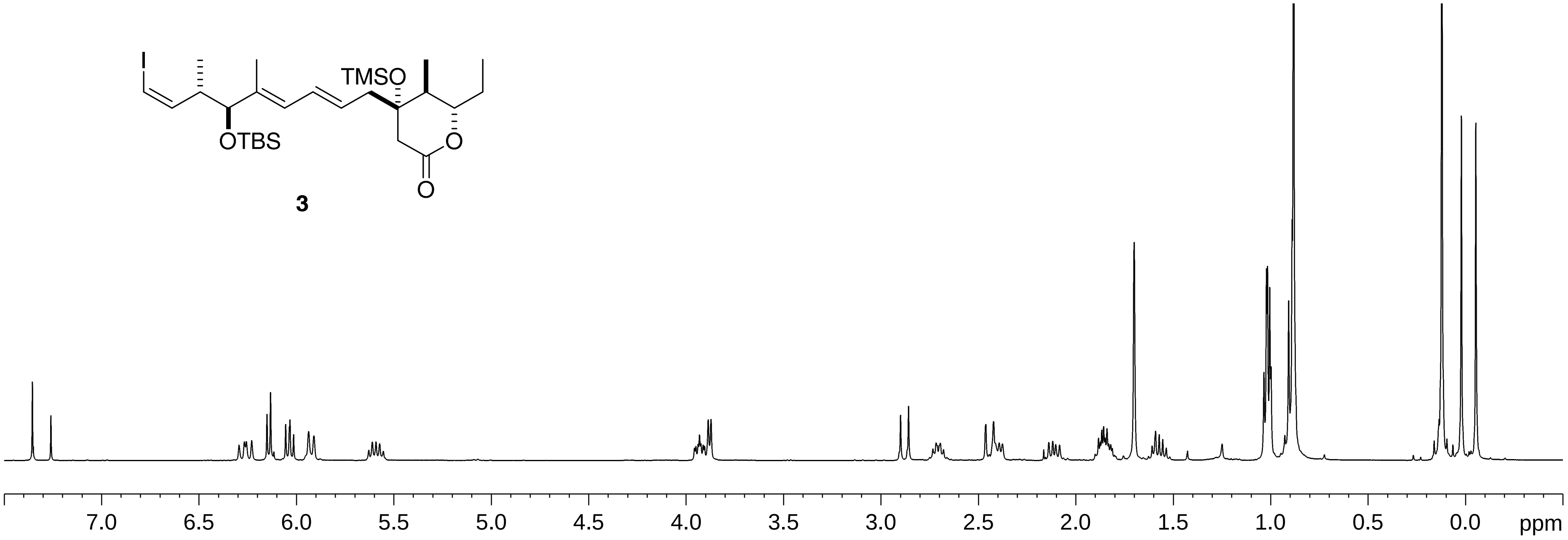
I
TMSO
OTBS
O
O
3
7.0
6.5
6.0
5.5
5.0
4.5
4.0
3.5
3.0
2.5
2.0
1.5
1.0
0.5
0.0
ppm

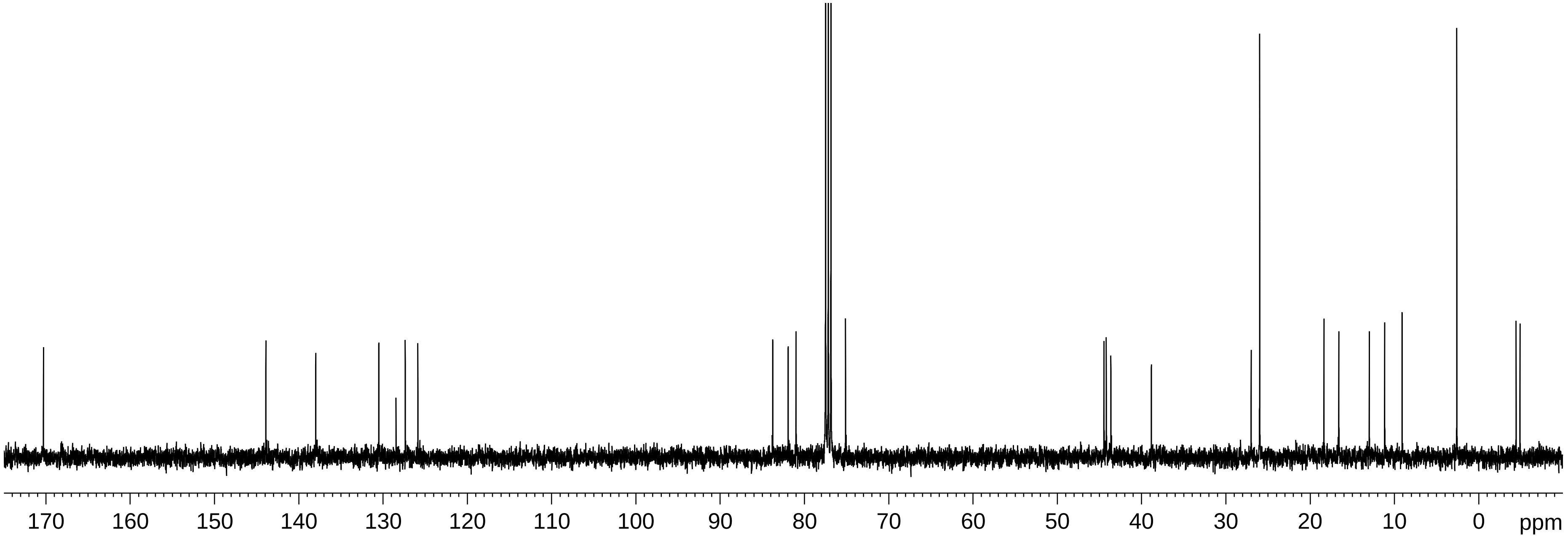
170
160
150
140
130
120
110
100
90
80
70
60
50
40
30
20
10
0
ppm

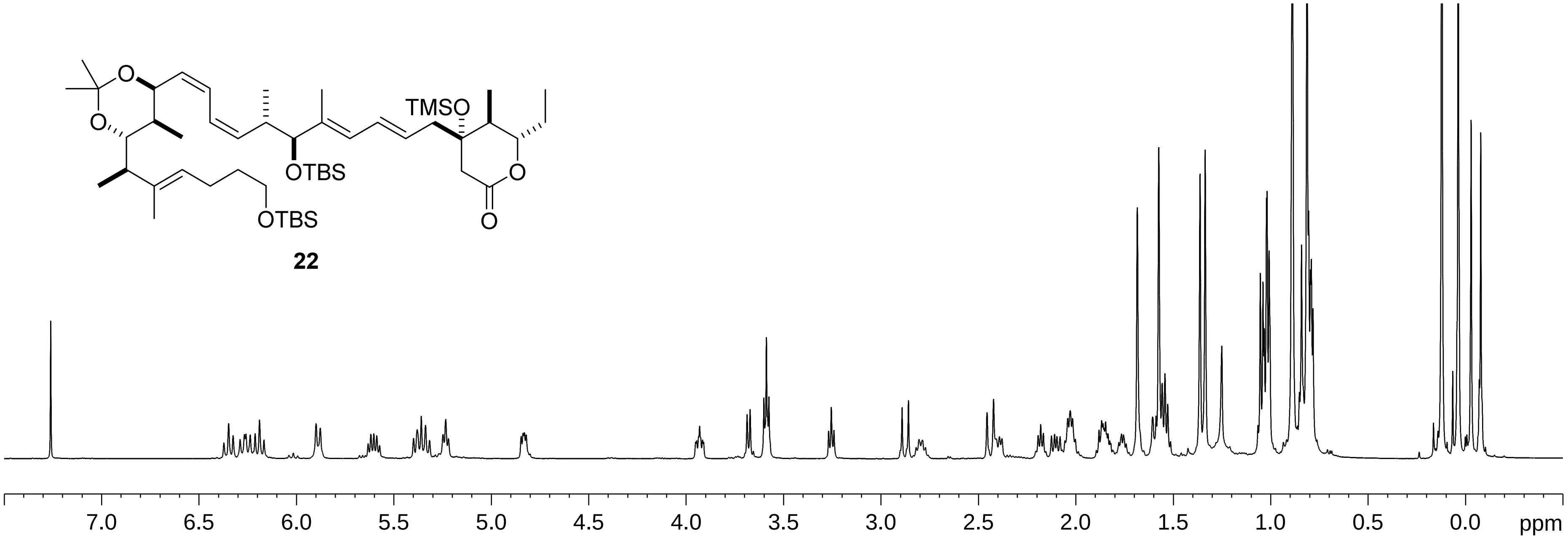
TMSO
OTBS
OTBS
7.0 6.5 6.0 5.5 5.0 4.5 4.0 3.5 3.0 2.5 2.0 1.5 1.0 0.5 0.0 ppm

22

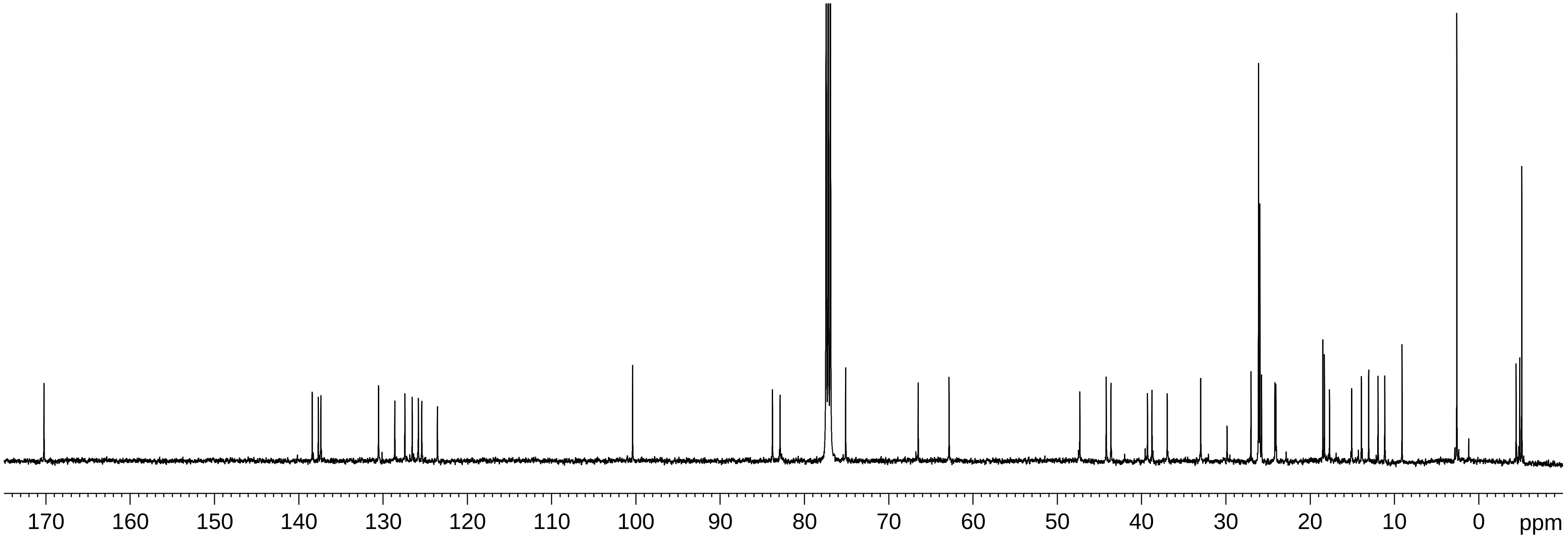
170 160 150 140 130 120 110 100 90 80 70 60 50 40 30 20 10 0 ppm

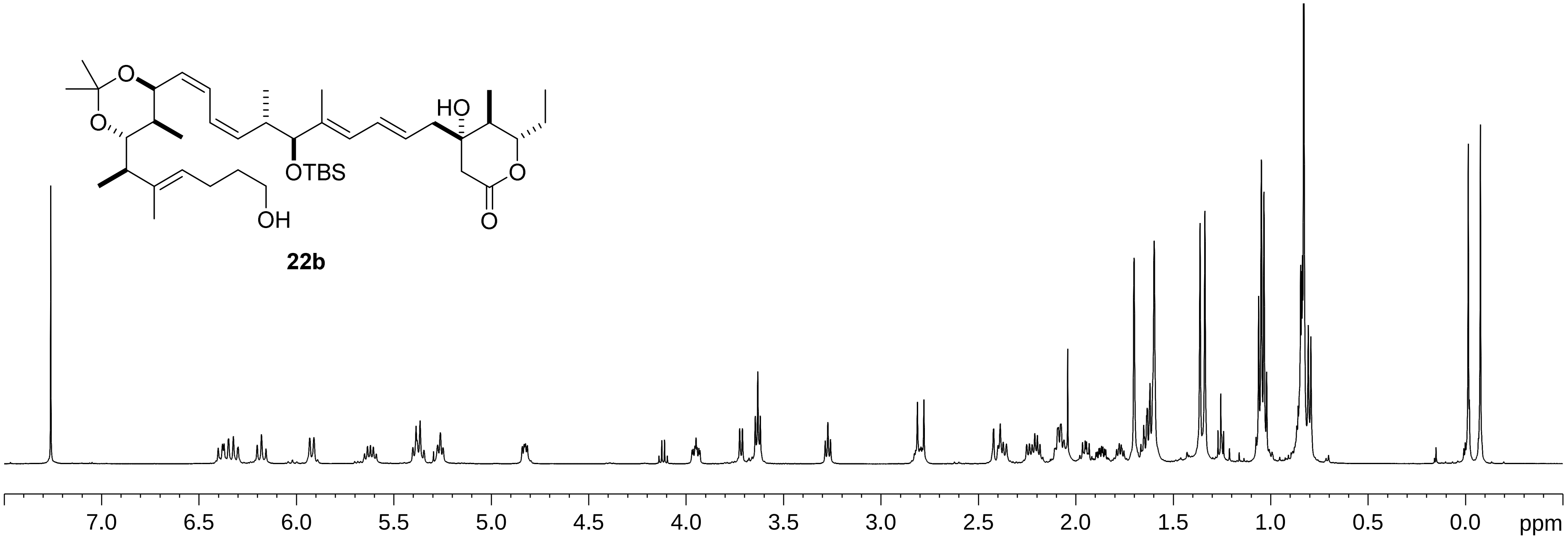
O
O
HO
OTBS
O
O
OH
7.0
6.5
6.0
5.5
5.0
4.5
4.0
3.5
3.0
2.5
2.0
1.5
1.0
0.5
0.0
ppm

22b

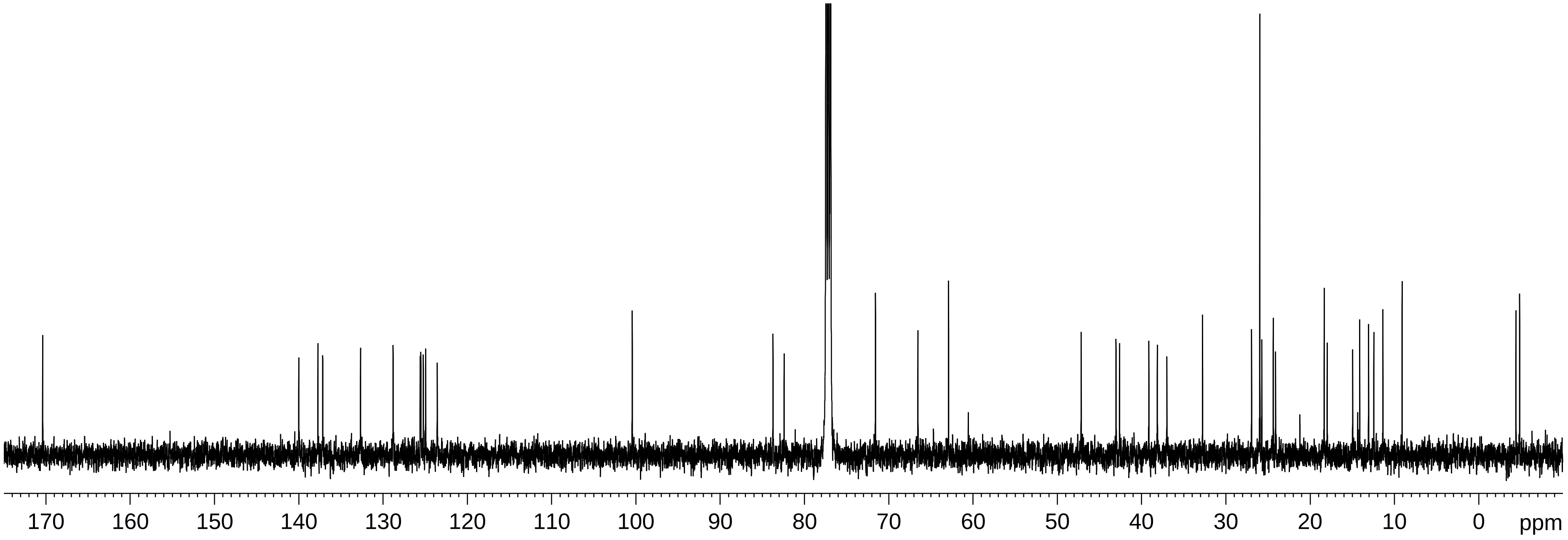
170
160
150
140
130
120
110
100
90
80
70
60
50
40
30
20
10
0
ppm

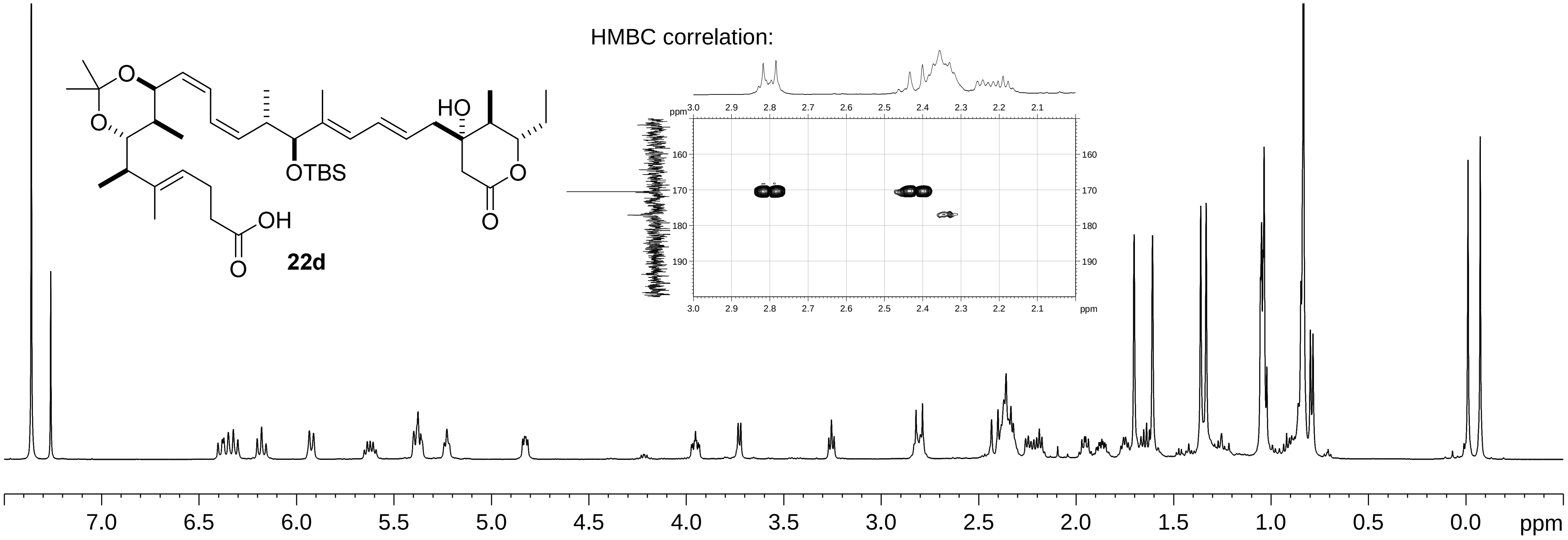
HMBC correlation:
HO
OTBS
OH
22d
ppm

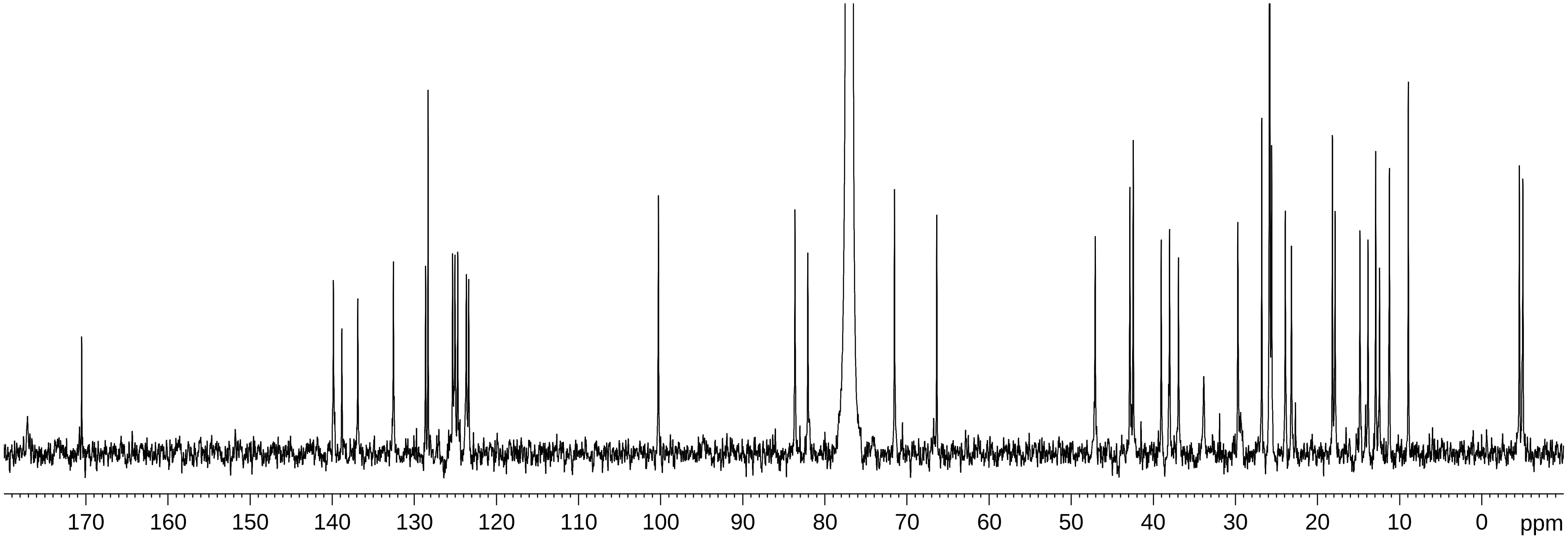
ppm

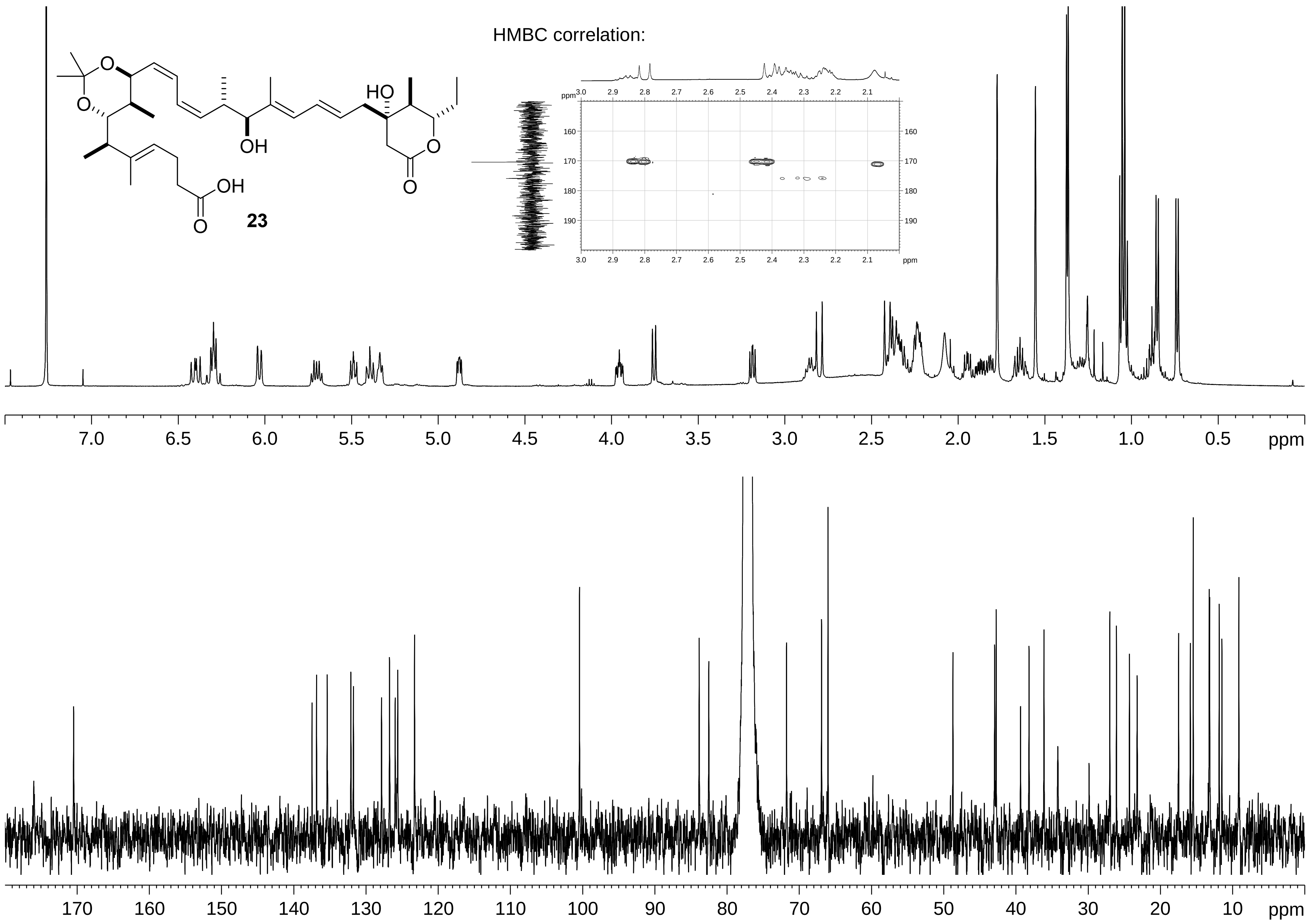
HMBC correlation:
HO
OH
O
OH
23
ppm

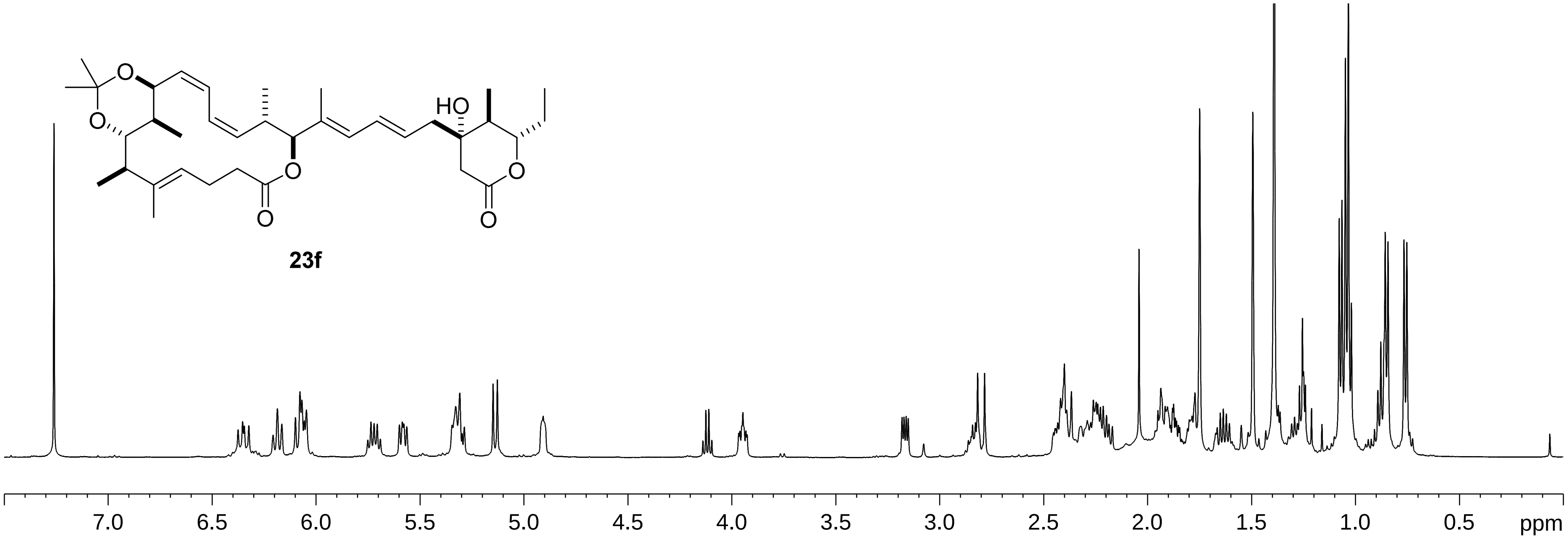
HO
23f
7.0 6.5 6.0 5.5 5.0 4.5 4.0 3.5 3.0 2.5 2.0 1.5 1.0 0.5 ppm

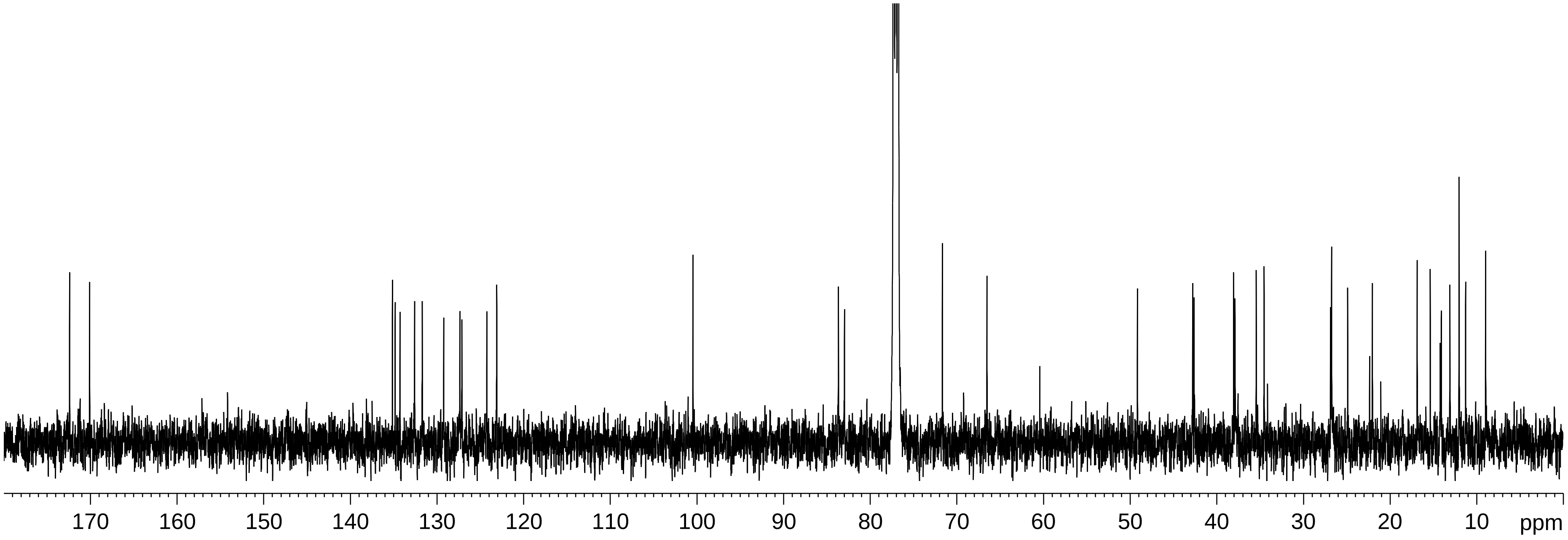
170 160 150 140 130 120 110 100 90 80 70 60 50 40 30 20 10 ppm

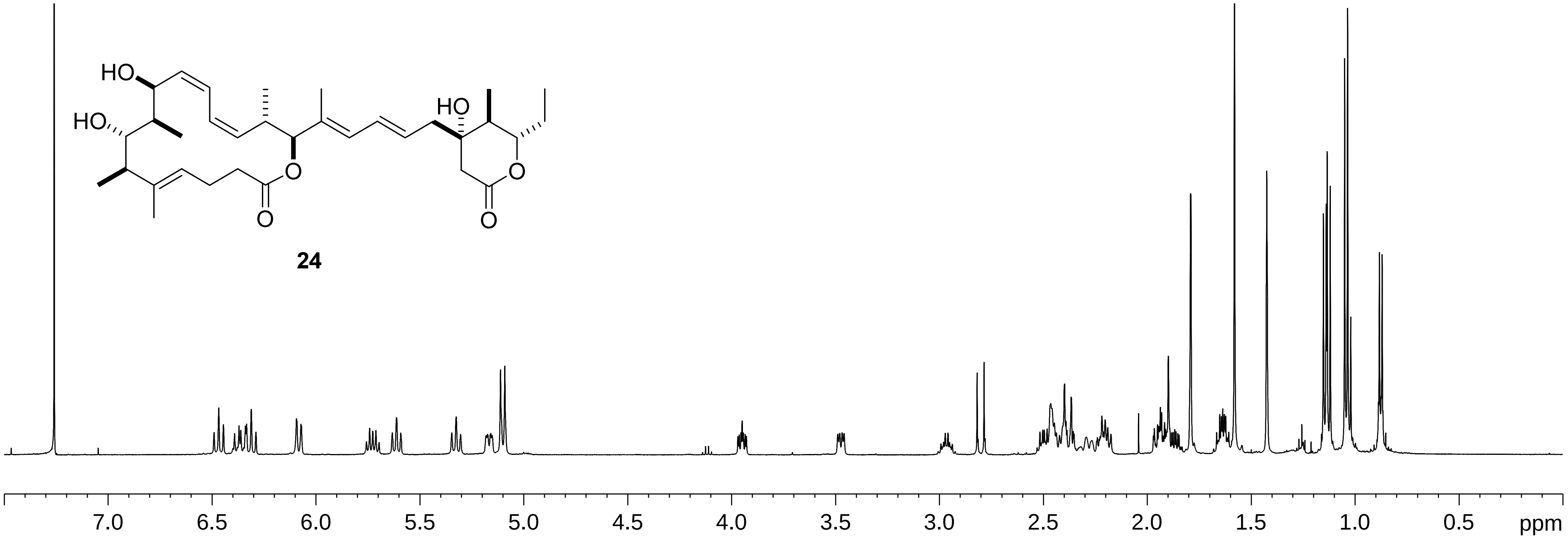
HO
HO
HO
O
O
O
O
24
7.0
6.5
6.0
5.5
5.0
4.5
4.0
3.5
3.0
2.5
2.0
1.5
1.0
0.5
ppm

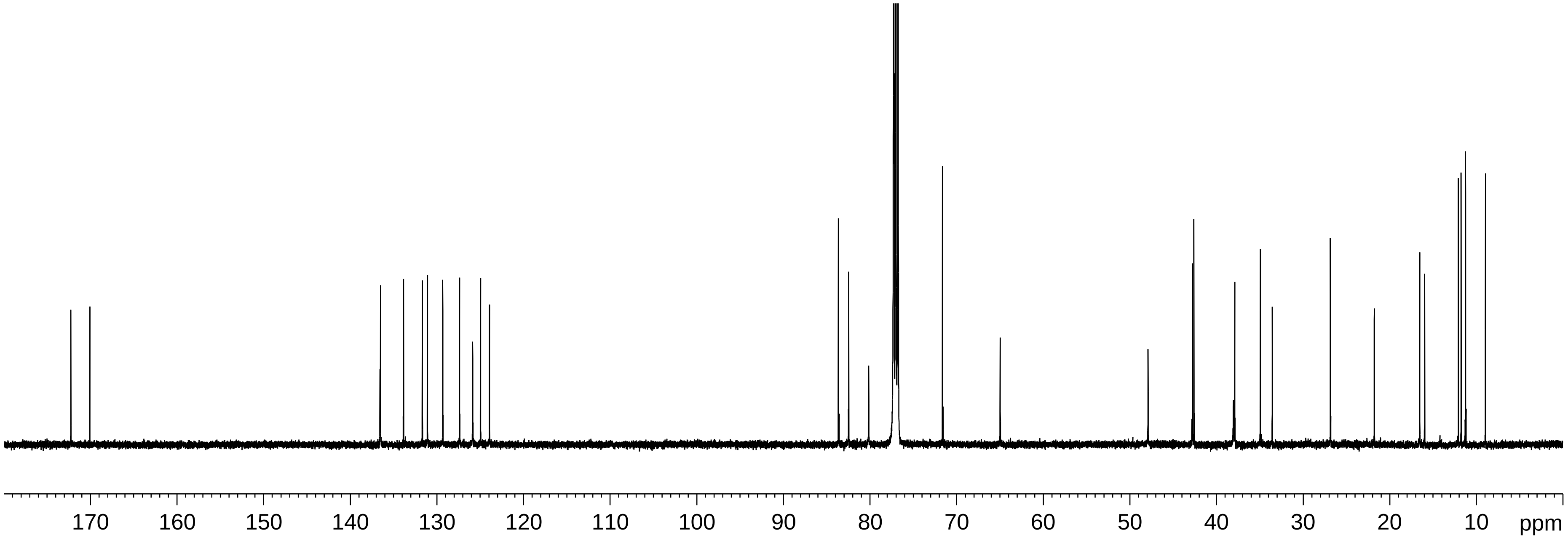
170
160
150
140
130
120
110
100
90
80
70
60
50
40
30
20
10
ppm

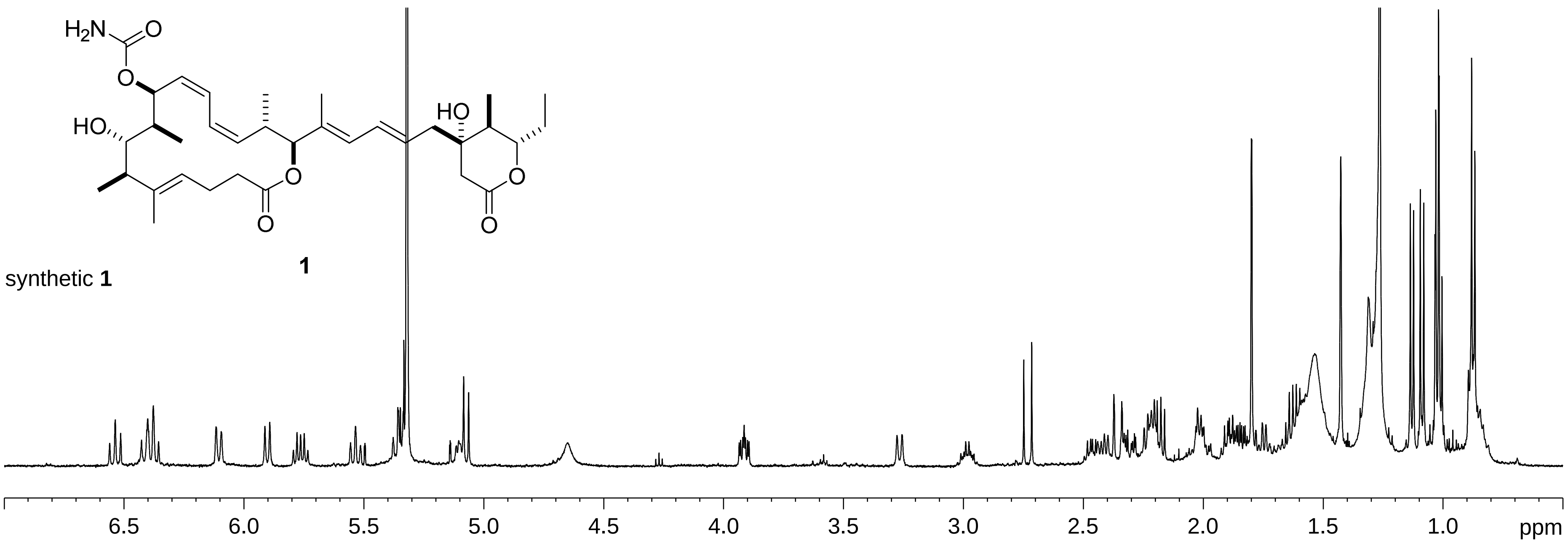
H2N
O
O
HO
HO
O
O
O
O
1
synthetic 1
6.5 6.0 5.5 5.0 4.5 4.0 3.5 3.0 2.5 2.0 1.5 1.0 ppm

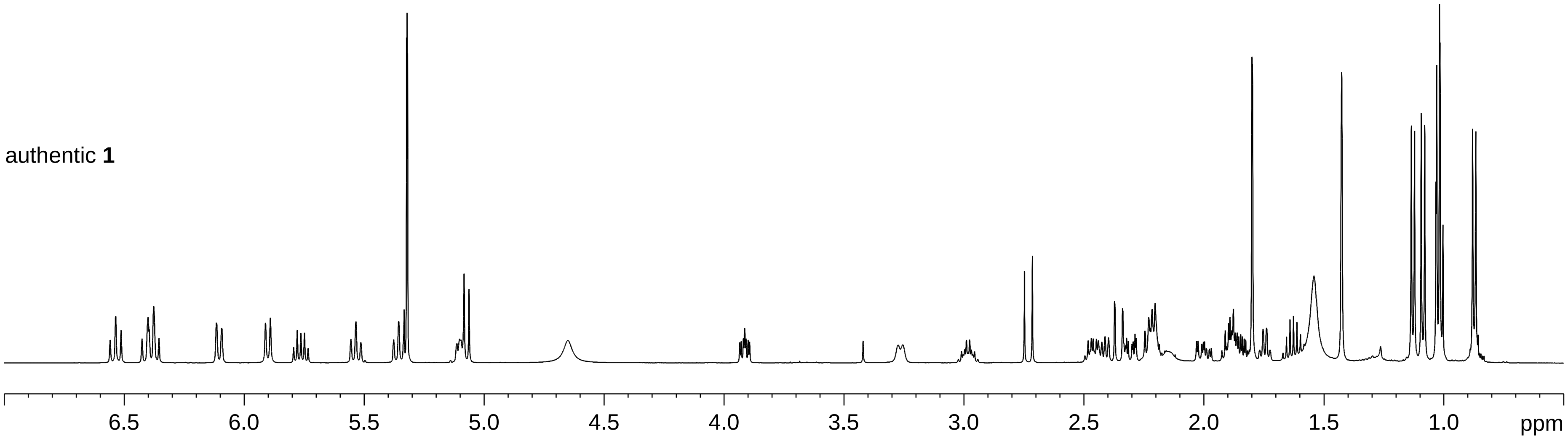
authentic 1
6.5 6.0 5.5 5.0 4.5 4.0 3.5 3.0 2.5 2.0 1.5 1.0 ppm

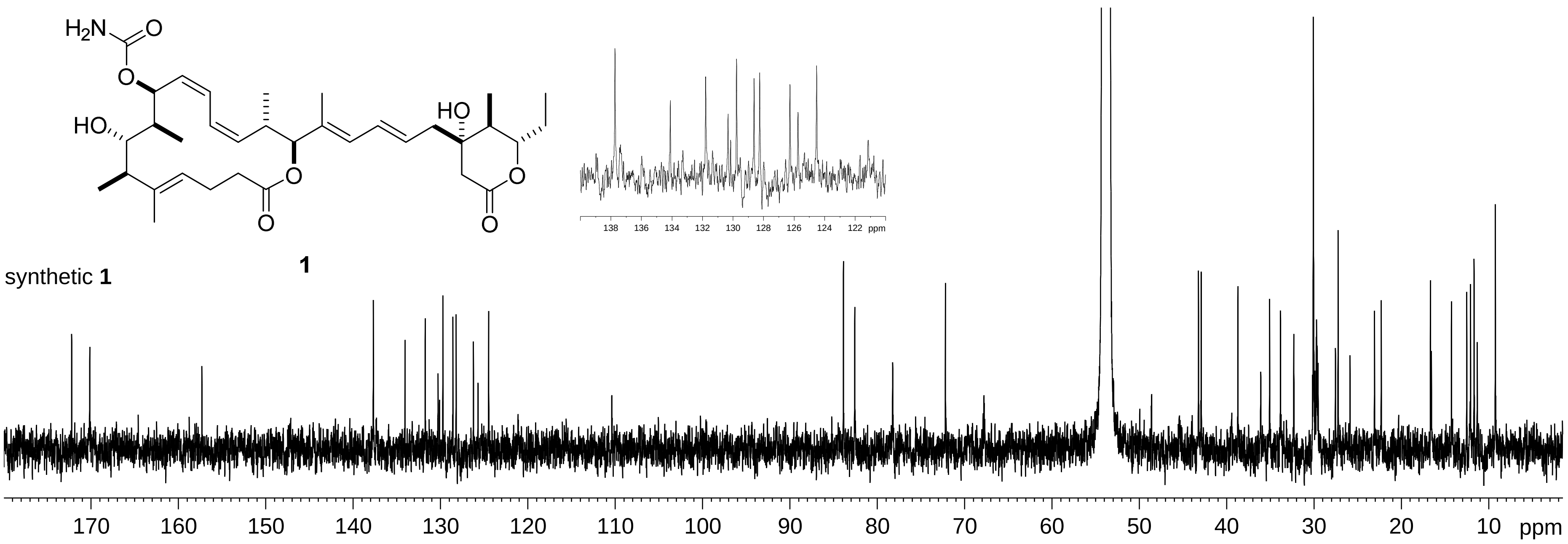

synthetic **1**

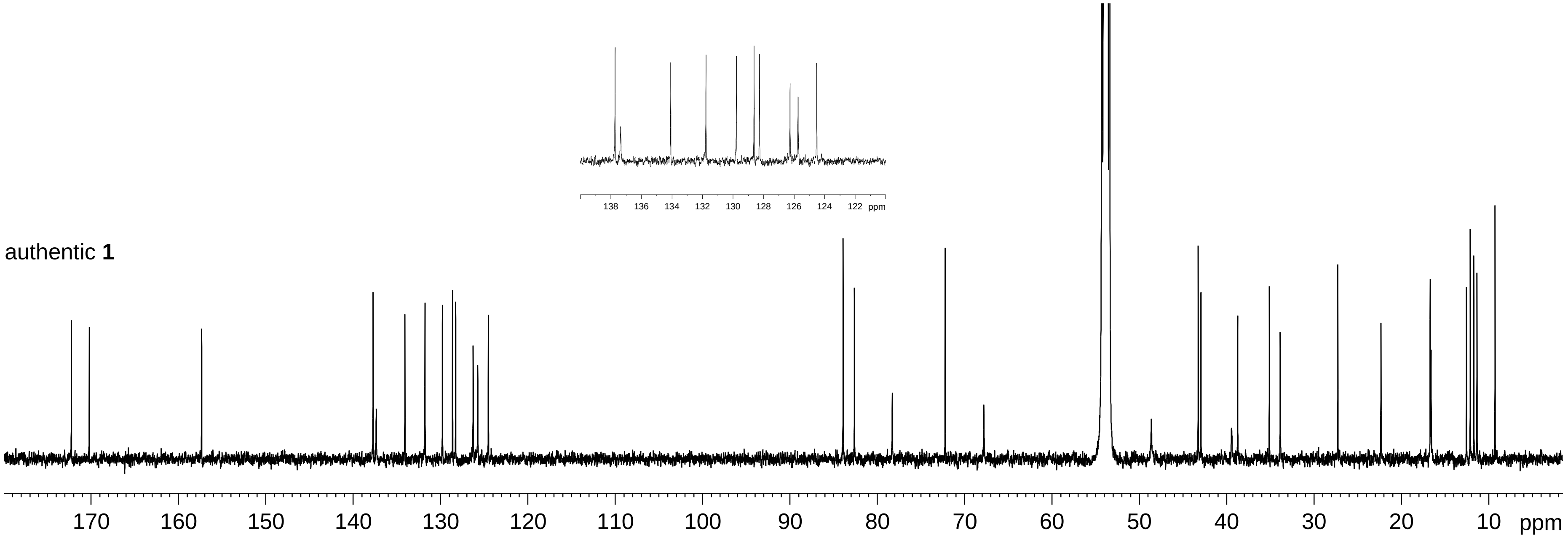

authentic **1**

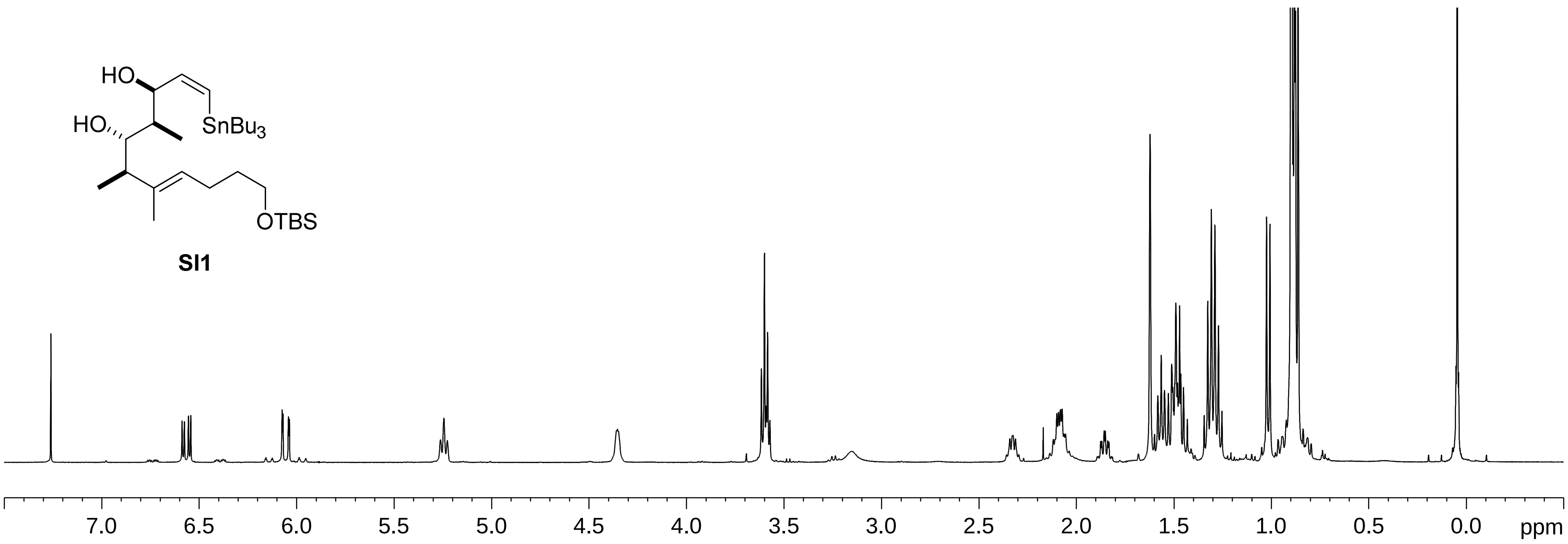
HO
HO
SnBu3
OTBS
SI1
7.0
6.5
6.0
5.5
5.0
4.5
4.0
3.5
3.0
2.5
2.0
1.5
1.0
0.5
0.0
ppm

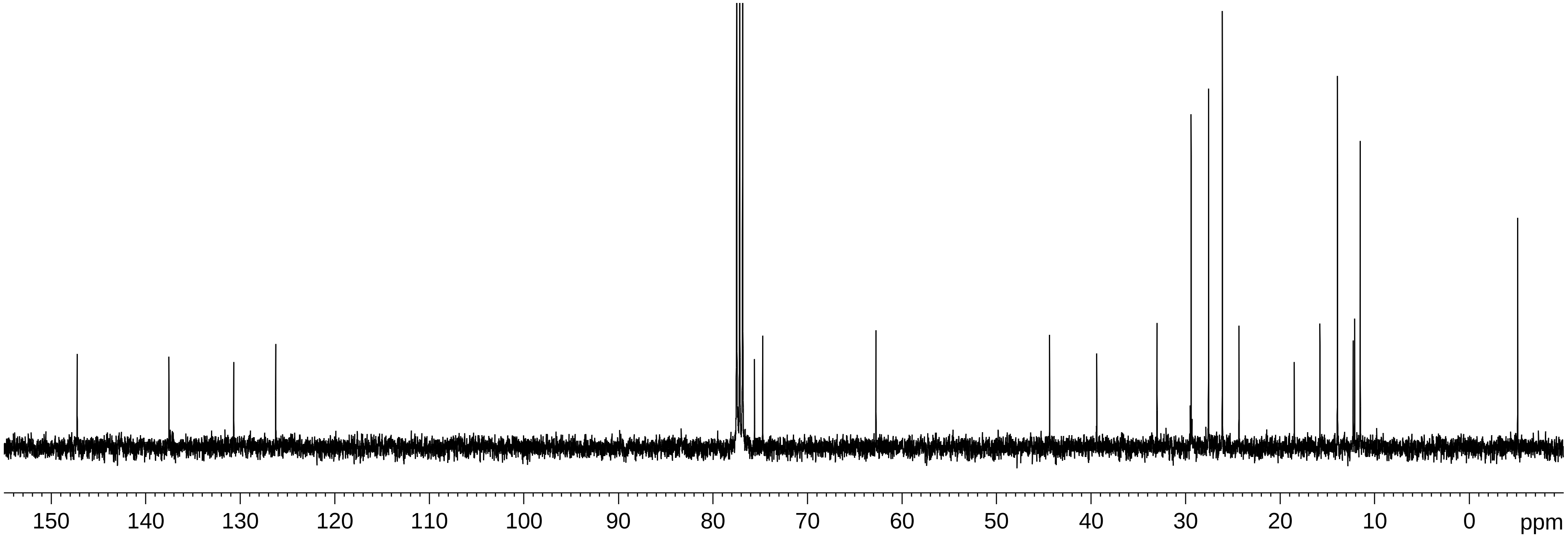
150
140
130
120
110
100
90
80
70
60
50
40
30
20
10
0
ppm

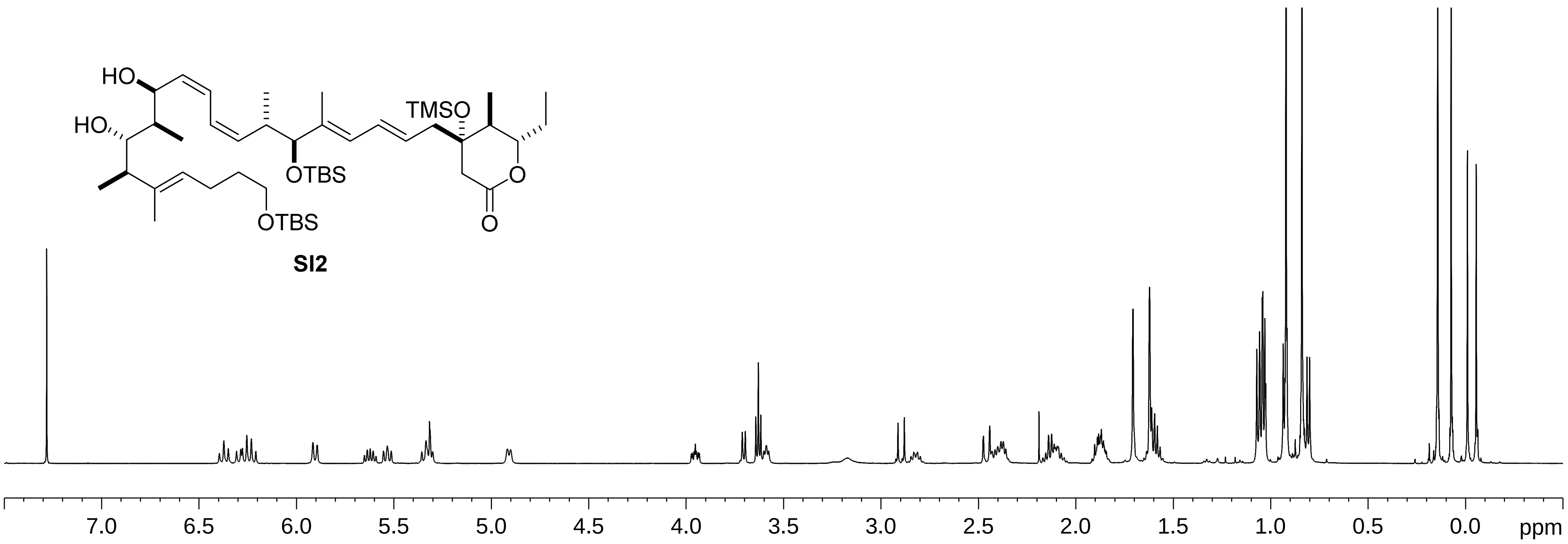
HO
HO
TMSO
OTBS
OTBS
O
O
SI2
7.0 6.5 6.0 5.5 5.0 4.5 4.0 3.5 3.0 2.5 2.0 1.5 1.0 0.5 0.0 ppm

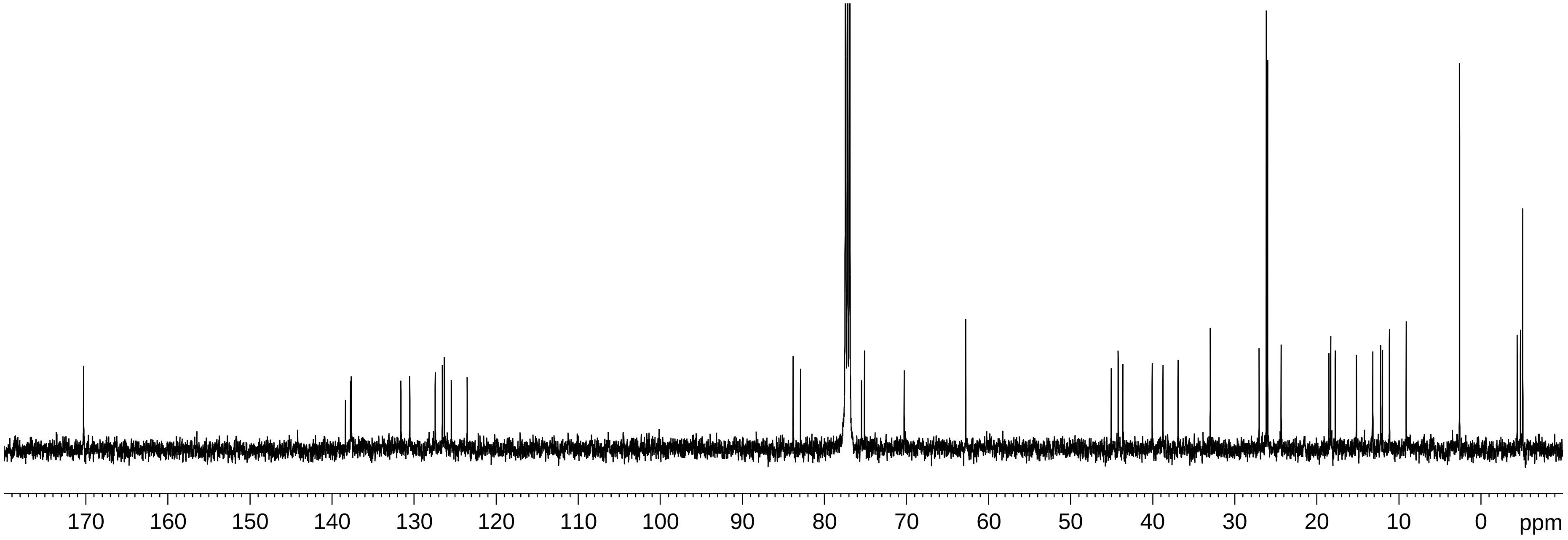
170 160 150 140 130 120 110 100 90 80 70 60 50 40 30 20 10 0 ppm

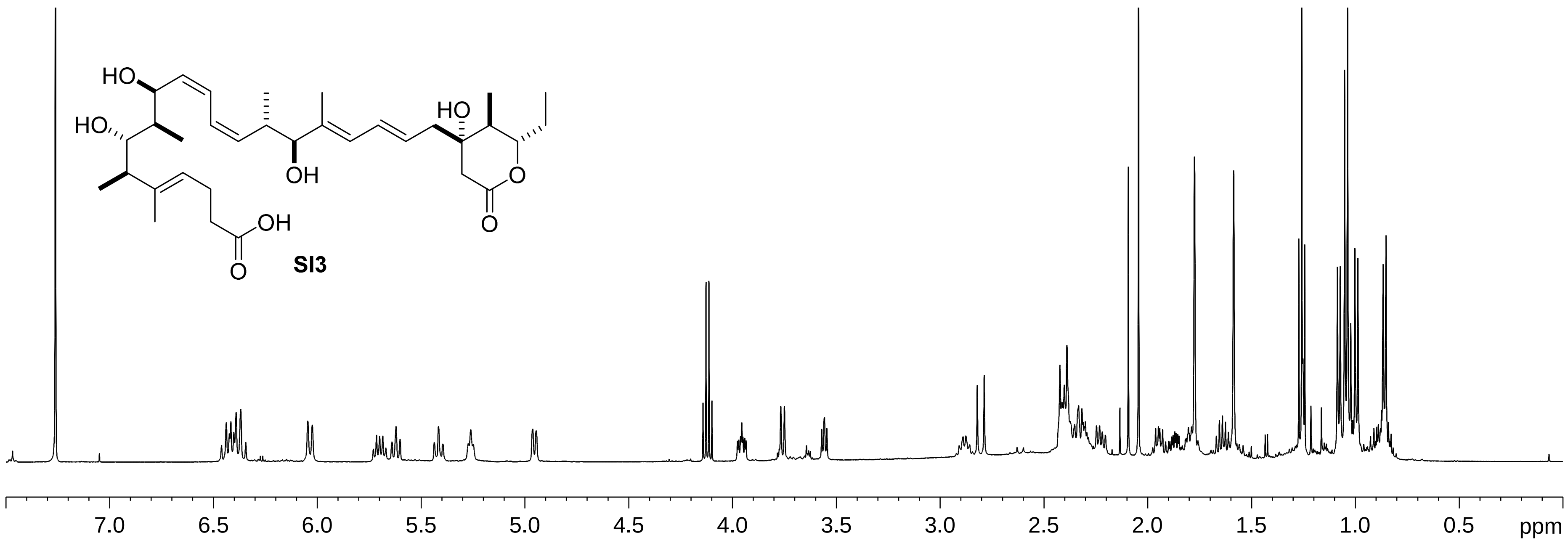
HO
HO
OH
HO
O
O
OH
O
SI3
7.0 6.5 6.0 5.5 5.0 4.5 4.0 3.5 3.0 2.5 2.0 1.5 1.0 0.5 ppm

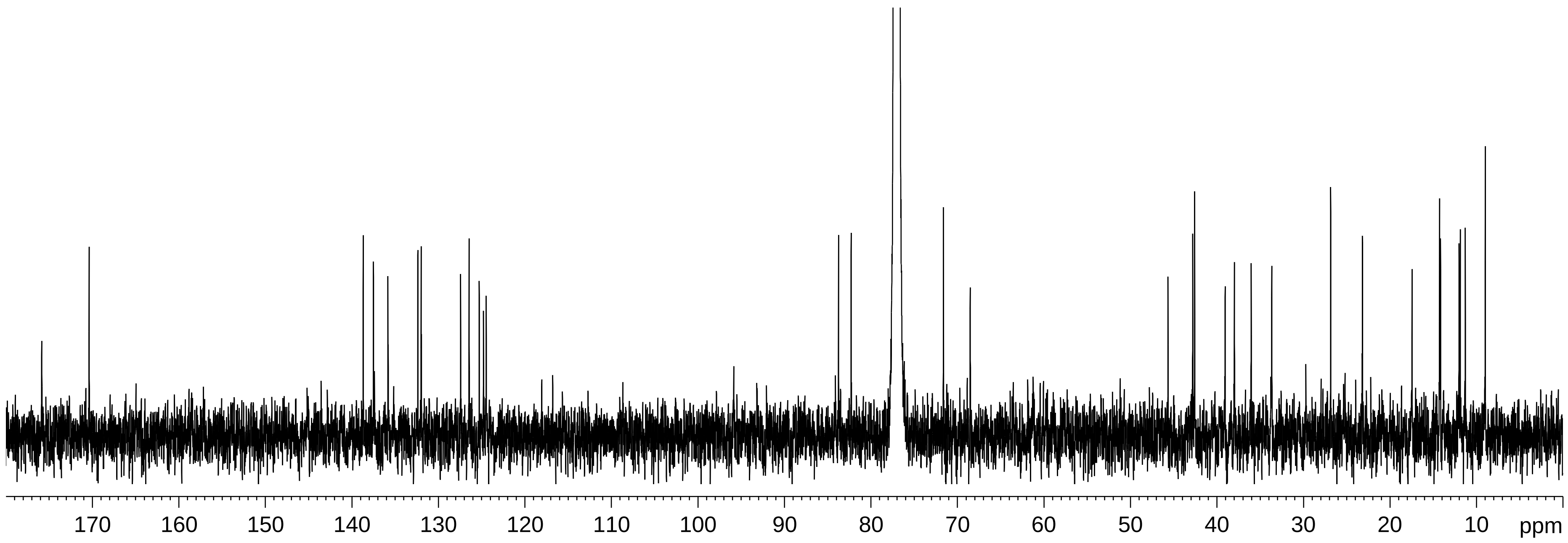
170 160 150 140 130 120 110 100 90 80 70 60 50 40 30 20 10 ppm

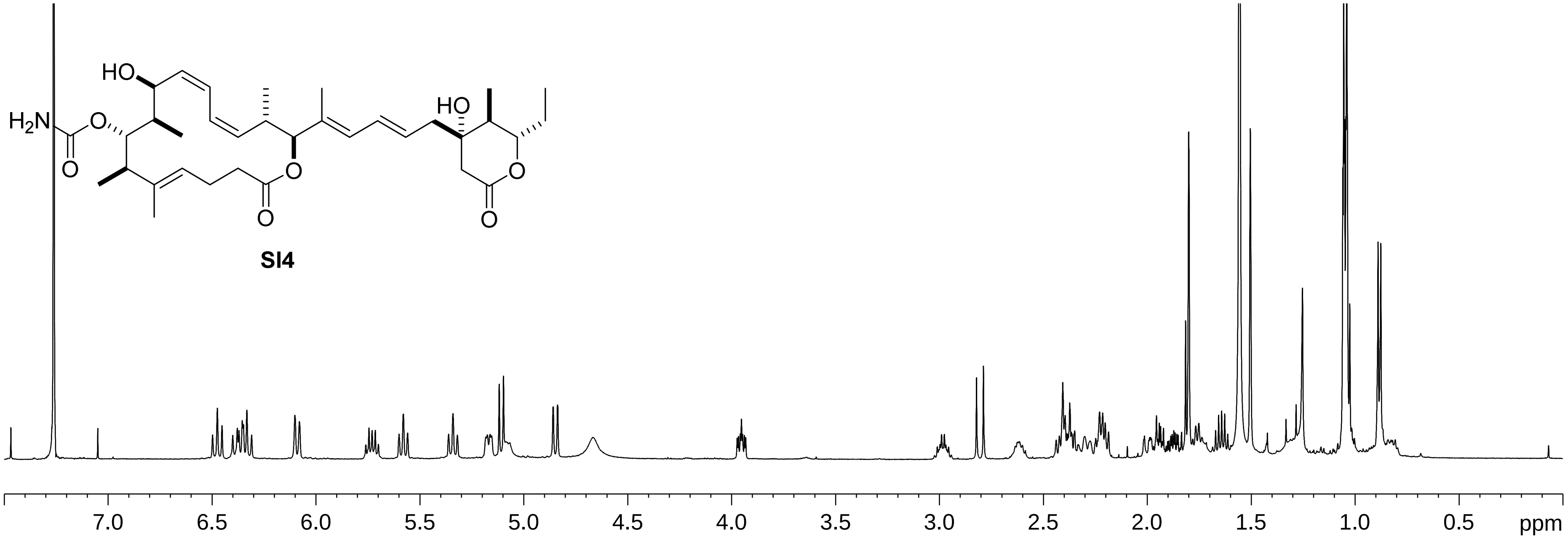

HO
H_2N
O
HO
O
O
O
O
O
SI4
7.0
6.5
6.0
5.5
5.0
4.5
4.0
3.5
3.0
2.5
2.0
1.5
1.0
0.5
ppm

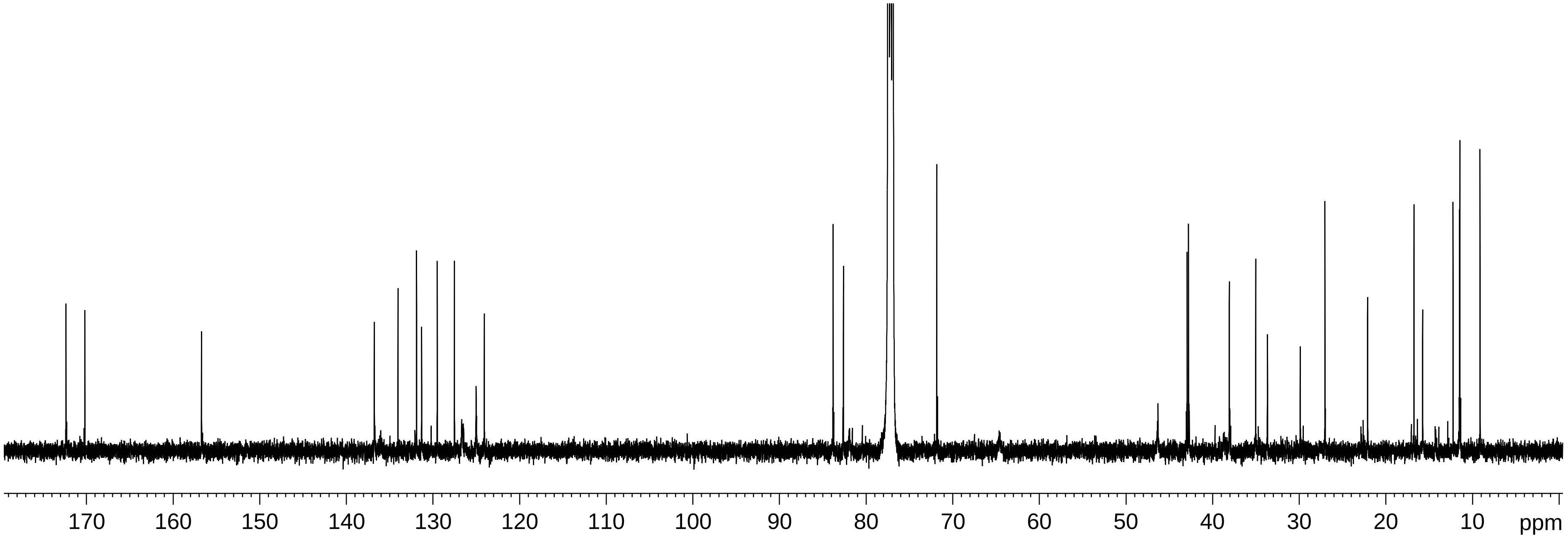

170
160
150
140
130
120
110
100
90
80
70
60
50
40
30
20
10
ppm
